# Electronic Synergistic Effects on the Stability and Oxygen Evolution Reaction Efficiency of the Mesoporous LiMn_2–*x*_M_*x*_O_4_ (M = Mn, Fe, Co, Ni, and Cu) Electrodes

**DOI:** 10.1021/acs.inorgchem.4c03885

**Published:** 2024-11-07

**Authors:** Irmak Karakaya Durukan, Ömer Dag

**Affiliations:** † Department of Chemistry, Bilkent University, 06800 Ankara, Turkey; ‡ UNAM—National Nanotechnology Research Center and Institute of Materials Science and Nanotechnology, Bilkent University, 06800 Ankara, Turkey

## Abstract

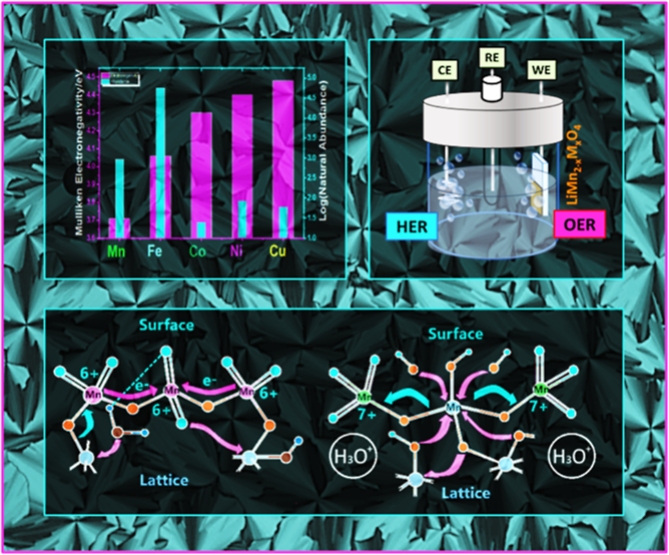

Stable porous manganese oxide-based electrodes are essential for clean energy generation and storage because of their high natural abundance and health safety. This investigation focuses on mesoporous LiMn_2–*x*_M_*x*_O_4_ (where M is Fe, Co, Ni, and Cu and *x* is 0, 0.1, 0.3, 0.5, and 0.67) electrodes and thin/thick films. The mesoporous electrodes and films are fabricated by coating clear and homogeneous ethanol solutions of the salts (LiNO_3_, [Mn(OH_2_)_4_](NO_3_)_2_, and [M(OH_2_)_*x*_](NO_3_)_2_) and surfactants (P123 and CTAB) and calcining at elevated temperature (denoted as F-LiMn_2–*x*_M_*x*_O_4_, G-LiMn_2–*x*_M_*x*_O_4_, and *meso*-LiMn_2–*x*_M_*x*_O_4_, respectively). The electrochemical properties, stability, and oxygen evolution reaction (OER) performance of the F/G-LiMn_2–*x*_M_*x*_O_4_ electrodes are investigated in alkaline media using a three electrode setup. The F-LiMn_1.33_M_0.67_O_4_ electrodes (where M is Mn, Fe, Co, and Ni) exhibit low Tafel slopes of 60, 43, 44, and 32 mV/dec, respectively. While all the Mn-rich and F-LiMn_2–*x*_Fe_*x*_O_4_ electrodes degrade via Mn(VI) disproportionation reaction, the 33% Co electrode shows high stability during the OER. The nickel-based electrodes are stable with as little as 15% Ni and display excellent OER performance over 25% Ni, albeit undergoing a transformation that accumulates Ni(OH)_2_ species on the electrode surface. Copper in the F-LiMn_2–*x*_Cu_*x*_O_4_ electrodes is homogeneous at low Cu percentages but forms a CuO phase above 15% Cu, undergoes degradation, and displays a weak OER performance. In short, Co and Ni stabilize the F-LiMn_1.33_Co_0.67_O_4_ and F-LiMn_1.7_Ni_0.3_O_4_ electrodes, which display excellent OER performance.

## Abstract 2

Highly abundant and less toxic manganese-based electrocatalysts can enhance clean energy production processes, such as the oxygen evolution reaction (OER). However, these electrocatalysts often exhibit instability due to disproportionation reactions (DRs). To develop stable electrodes for OER, it is essential to address the challenges posed by the Mn(VI)-induced disproportionation. The molten salt self-assembly method can yield stable manganese oxide-based electrodes by incorporating more electronegative transition metals, like cobalt and nickel.

## Introduction

Manganese (Mn) oxide-based stable electrodes can revolutionize electrochemical hydrogen production as a clean energy source.^1,2^ Because Mn is one of the most abundant transition metals on the earth’s crust and is more environmentally benign than the other transition metals.^3^ However, the Mn-based materials undergo disproportionation reactions (DRs) at +3 and +6 oxidation states under alkaline media.^4−10^ The Mn(III) species disproportionate into Mn(II) and Mn(IV) species, where the Mn(IV) is stable as MnO_2_, but Mn(II) may dissolve into an electrolyte solution. The Mn(VI) species also undergo DR to produce stable manganese dioxide (Mn(IV)) species and Mn(VII) (dissociate as [MnO_4_]^−^ ion from the electrode surface).^8−10^ Therefore, the starting electrode material slowly transforms to MnO_2_. Eventually, the MnO_*x*_ degrades in an alkaline media during an oxygen evolution reaction (OER) by forming permanganate species.^8−10^ Among the MnO_2_ phases, the λ-MnO_2_ phase is the most robust Mn oxide for OER^11^ and is produced by lithium–ion delithiation of spinel LiMn_2_O_4_ (LiMn_2_O_4_ → λ-MnO_2_ + Li^+^ + e^–^).^11−18^ However, no matter which phase, the Mn species gradually degrade under alkaline media via oxidization of the surface Mn species to manganate that undergoes a gradual DR. Delaying or preventing the Mn(VI)-DR is essential for developing Mn-based electrocatalysts for OER.

The Mn(VI)-DR occurs via the oxidation of two Mn(VI) to two Mn(VII) and the reduction of one Mn(VI) to Mn(IV) (3Mn(VI) → Mn(IV) + 2Mn(VII)).^11^ Therefore, three Mn(VI) sides are necessary for efficient DR on the electrode surface. We recently proposed a mechanism for the Mn(VI)-DR based on the electronegativity difference among these three Mn(VI) sides on the electrode surface.^11^ In the suggested model, three Mn(VI) sides, with the middle Mn(VI) having a local composition of Mn_3_O_8.66_ and a mean electronegativity of 6.28 eV (calculated from Sanderson’s electronegativity using the geometric mean of the electronegativity),^19^ and the other two Mn(VI) in each side are bonded to the MnO_2_ lattice with a local composition of Mn_3_O_5.83_ and a mean electronegativity of 5.92 eV.^11^ Therefore, for every 3Mn(VI) [lattice–Mn(O)_2_–O–Mn(=O)_2_–O–Mn(O)_2_–lattice], the middle Mn(VI) is oxygen-rich and more electronegative and energetically favors withdrawing electrons from the less electronegative Mn(VI) sides (attached to Mn(IV) lattice) to carry the DR.^11^ Replacing one of these Mn sides with a more electronegative transition metal ([lattice–Mn(VI)–M(III)–Mn(VI)_2_–lattice], such as M is Fe, Co, or Ni) may slow or even stop the Mn(VI)-DR. A stable Mn(VI) side can effectively carry the OER, where the Mn(VI)=O sites (oxo-bonds) are good electrophiles because of +6 oxidation state of Mn and react with OH^–^ ions through a nucleophilic redox reaction to form Mn–O–OH (–Mn(VI)=O + OH^–^ → –Mn(IV)–O–OH), where the oxygen–oxygen bond forms (peroxide formation, oxygens are −1 charged).^11,20−22^ The O–O bond formation is the rate-determining step in the reaction mechanism of water oxidation in alkaline media, as previously suggested in an earlier publication.^11^ Also note that the source of OH^–^ for the above reaction is the self-dissociation of water (2H_2_O ↔ OH^–^ + H_3_O^+^) that produces equal amounts of OH^–^ and H_3_O^+^ ions.^20^ The OH^–^ ion is consumed in the reaction described above (Mn–O–OH bond formation). Therefore, the H_3_O^+^ ion concentration increases on the electrode surface during the OER and plays a vital role in the stability of the electrodes. Since Mn is one of the abundant elements with multiple oxidation states, obviously there are many investigations in the literature to improve LiMn_2_O_4_ electrode performance and stability by incorporating transition metal ions into the structure.^23−27^ Introducing nickel or cobalt ions into LiMn_2_O_4_ spinel structure improves the stability and performance of the LiMn_2–*x*_M_*x*_O_4_ electrodes.^23−27^ This is due to a higher electronegativity of these metal ions compared to Mn; incorporation of Ni or Co to the Mn sides makes the remaining Mn sides oxidize at relatively higher potentials (electronic synergistic effect) and blocks the electron transfer between the Mn(VI) sides for the Mn(VI)-DR.

The λ-MnO_2_ is an essential phase for the stability of the Mn oxide-based electrodes;^11^ therefore, mesoporous LiMn_2_O_4_ or LiMn_2–*x*_M_*x*_O_4_ electrodes are produced and used to obtain the proper oxide phase, and as a transition metal ion, Fe^3+^, Co^2+^, Ni^2+^, and Cu^2+^ are chosen to test our hypothesis. The electrodes are produced by employing the molten salt-assisted self-assembly (MASA) method that is unique and applicable to obtain smooth thin films and electrodes of these materials; it involves preparation of a lyotropic liquid crystalline (LLC) mesophase of salts-surfactants as a thin coating over a conducting substrate and calcination above 300 °C.^11,20−22,28−30^ This method has already been successfully employed to produce mesoporous thin films of single, two, and three metal oxides (namely, NiO,^28^ MCo_2_O_4_ (M is Mn, Ni, and Zn),^21^ and LiMn_2–*x*_Co_*x*_O_4_,^20^ respectively), and it is also flexible to introduce more metals to these oxides. Here, we employed the MASA method to fabricate mesoporous LiMn_2–*x*_M_*x*_O_4_ electrodes over fluorine-doped tin oxide (FTO) or graphite rods. Their electrochemical properties, stability, and OER performance are investigated in a three-electrode cell in alkaline media.

## Experimental Section

### Preparation Salt-Surfactant Solutions and LLC Mesophases

The solutions of salts and surfactants are prepared using salts (LiNO_3_, [Mn(OH_2_)_4_](NO_3_)_2_, and [M(OH_2_)x](NO_3_)_2_ (M = Fe, Co, Ni, and Cu)), surfactants (Pluronic P123 as a nonionic surfactant and cetyltrimethylammonium bromide (CTAB) as a charged surfactant), concentrated HNO_3_ (65%), and absolute ethanol (99.9%).

First, 0.125 mmol (725.0 mg) of P123 is wholly dissolved in 5 g of ethanol by stirring, and then 0.125 mmol (45.56 mg) of CTAB is added to the solution. To this clear solution, first, 2.5 mmol (172.4 mg) LiNO_3_ salt is added at once, and after 5 min, concentrated HNO_3_ (500.0 mg) is added dropwise and stirred to obtain a homogeneous clear solution. The transition metal salts (total 5 mmol) are added to the clear solution sequentially at 5 min intervals. Then, the vial is sealed and stirred for a day to obtain a homogeneous solution. The mole ratios and quantities of each ingredient for all solutions are given in Table S1.

The solutions are coated by either drop-casting or spin-coating methods. The thick films are prepared on microscope slides using eight drops of the homogeneous solutions and aged for gelation for 1 h to form thick LLC films. A spin coating method is employed to obtain LLC thin films by using a few drops of the solution that is put on a glass substrate and spun at 2000 rpm for 10 s (denoted as LLC-LiMn_2–*x*_M_*x*_, where M is Fe, Co, Ni, and Cu and *x* is 0, 0.1, 0.3, 0.5, and 0.67). The thin films are used for small- and wide-angle X-ray diffraction (XRD) analysis. Schematic representations of the drop-casting and spin-coating methods are shown in Scheme S1.

### Synthesis of Mesoporous LiMn_2–*x*_M_*x*_O_4_ Powders, Films, and Electrodes

The drop-casted and spin-coated LLC-LiMn_2–*x*_M_*x*_ films are calcined directly at 300 °C for 3 and 2 h, respectively, denoted as *meso*-LiMn_2–*x*_M_*x*_O_4_. Then, the thick samples are scraped from the glass surfaces as powders and used for N_2_ adsorption–desorption measurements. Thin films are used for the powder XRD technique.

FTO is used to fabricate the mesoporous electrodes (denoted as F-LiMn_2–*x*_M_*x*_O_4_). Part of a FTO glass is taped to create a 1 cm^2^ FTO surface available for coating, then inserted over a spin coater, and each solution of the corresponding compositions is dropped and spun at 5000 rpm for 10 s to obtain a FTO-coated thin LLC-LiMn_2–*x*_M_*x*_ film. After coating, the tape is removed, and the gel film is calcined at 300 °C for 1 h. X-ray photoelectron spectroscopy (XPS) analysis and scanning electron microscopy (SEM) imaging are directly conducted on the FTO surface. The fabrication method and photographs of a set of FTO-coated electrodes are shown in Scheme S2.

### Instrumentation

#### X-ray Diffraction

Small- and wide-angle XRD patterns of the liquid crystalline films on the glass substrates are recorded using a Rigaku Miniflex diffractometer, equipped with a Cu Kα (1.54056 Å) X-ray source, operated at 30 kV and 15 mA. The small-angle XRD patterns are recorded between 1 and 5°, 2θ, with a scan rate of 0.5°/min. The wide-angle XRD patterns of the films are recorded between 10 and 80°, 2θ, with a scan rate of 3°/min. The wide-angle XRD patterns of powders and mesoporous thin films are collected using a Panalytical multipurpose X-ray diffractometer equipped with a Cu Kα (1.54056 Å) X-ray source, operated at 45 kV and 40 mA. The powder materials are packed smoothly into a silicon holder. The XRD patterns are recorded between 10 and 80°, 2θ, with a 1°/min scan rate. PDF cards of the Joint Committee on Powder Diffraction Standards (JCPDS) are used to identify and index the diffraction patterns of the samples.

#### X-ray Photoelectron Spectroscopy

XPS spectra are obtained using a Thermo Scientific K-alpha photoelectron spectrometer equipped with an Al K_α_ monochromatic source (1486.68 eV) and a 400 μm spot size under ultrahigh vacuum conditions. The film spectra are directly recorded on the FTO surface. The FTO surface is contacted by the sample holder of the device with a metal ribbon to compensate for the charging of the material. All spectra are calibrated according to C 1s peak at 284.8 eV, and elemental percentages are analyzed using the XPS survey spectra.

#### Scanning Electron Microscopy

SEM images of the thin films are directly recorded using a (FEI) Quanta 200 F scanning electron microscope at 15 keV beam energy under high vacuum conditions. The FTO-coated films are placed on a conductive stub. Conductive parts of the films are contacted with stubs with the help of carbon tape to allow electron flow.

#### N_2_ (77.4 K) Adsorption–Desorption Isotherms

100 mg portion of a powder is obtained by scraping thick films. Before the measurement, the powder samples are dehydrated under vacuum conditions at 200 °C for 2 h. The isotherms are collected by using a TriStar 3000 (Micrometrics) in the relative pressure range of 0.01–0.99 atm. Surface areas of the materials are estimated using the Brunauer–Emmett–Teller (BET) analysis method using 5 points in the range of 0.05 to 0.3*P*/*P*_0_. Pore size distributions are also evaluated by using the Barrett–Joyner–Halenda (BJH) method.

#### Electrochemical Analysis

Electrochemical experiments are performed using Gamry Instruments (Potentiostats PC14G750 and IFC5000-07565). A polypropylene (PP) cell is used to prevent the aging effect of the electrolyte. Ag/AgCl (3.5 M KCl) as the reference electrode (RE), a platinum wire as a counter electrode (CE), and FTO-coated LiMn_2–*x*_M_*x*_O_4_ electrodes as a working electrode (WE) are used in a three-electrode cell. The potential is converted and reported with respect to the reversible hydrogen electrode (RHE). A schematic representation of the electrochemical cell is shown in Scheme S3.

The electrochemical experiments are carried out in an alkaline medium (pH ∼ 14, 1 M KOH solution). Before all electrochemical measurements, nitrogen gas is purged into the electrolyte solution for 15 min to get rid of any dissolved oxygen. Measurement is started with cyclic voltammetry (CV) to analyze the general behavior of the electrodes. Then, multistep chronoamperometry (m-CA) is done by increasing potential sequentially with 0.01 V increments, and Tafel slope data are extracted from the plot of log(*j*) vs overpotential. Finally, chronopotentiometry (CP) experiments are performed at constant current densities from 1 to 50 mA/cm^2^ to get information about overpotential values for OER at different current densities and to check the WE stability in alkaline media. Nyquist plots of the films are constructed by potentiostatic electrochemical impedance spectroscopy recording between 200 kHz and 10 mHz with a 10 mV rms AC voltage amplitude.

### Electrochemical Investigation of Active Metal Sides on the F-LiMn_2–*x*_M_*x*_O_4_ Electrodes

Analysis has been carried out in several aqueous electrolyte solutions and a three-electrode setup (F-LiMn_2–*x*_M_*x*_O_4_ as WE, Ag/AgCl (3.5 M KCl) as RE, and platinum wire as CE). For neutral electrolytes, 1 M LiNO_3_ aqueous solutions are prepared and used to identify metal species on the electrode surface. The electrolytes are prepared by dissolving 1 mol LiNO_3_ (68.95 g) salt in a liter of aqueous solution (1 M LiNO_3_).

The F-LiMn_2–*x*_M_*x*_O_4_ electrodes are used to record 300 CVs between 0 and 1 V in a 1 M KOH solution. Then, the used electrodes are washed by first keeping the used electrode in deionized water under sonication for 30 s. Then, deionized water is replaced with fresh one, and the above procedure is repeated for the second and third time.

## Results and Discussion

### Salt-Surfactant Mesophases (LLC-LiMn_2–*x*_M_*x*_-P123)

A series of mesoporous LiMn_2–*x*_M_*x*_O_4_ (where M is Fe, Co, Ni, and Cu) thin films are prepared by employing the MASA method. The total metal salt/surfactant (P123) mole ratio is kept at 60 (20 Li(I), 40 (Mn(II) + M(II))), and *x* is varied (*x* = 0, 0.10, 0.30, 0.50, and 0.67) to prepare initial clear ethanol solutions. Then, the clear homogeneous solutions are spin-coated over FTO-coated glass substrates to obtain thin films of the LLC phase (gel-phase). The gel films are calcined at 300 °C for 1 h to produce mesoporous electrodes (denoted as F-LiMn_2–*x*_M_*x*_O_4_, where F stands for FTO and M is Mn, Fe, Co, Ni, or Cu).

**Figure 1:**
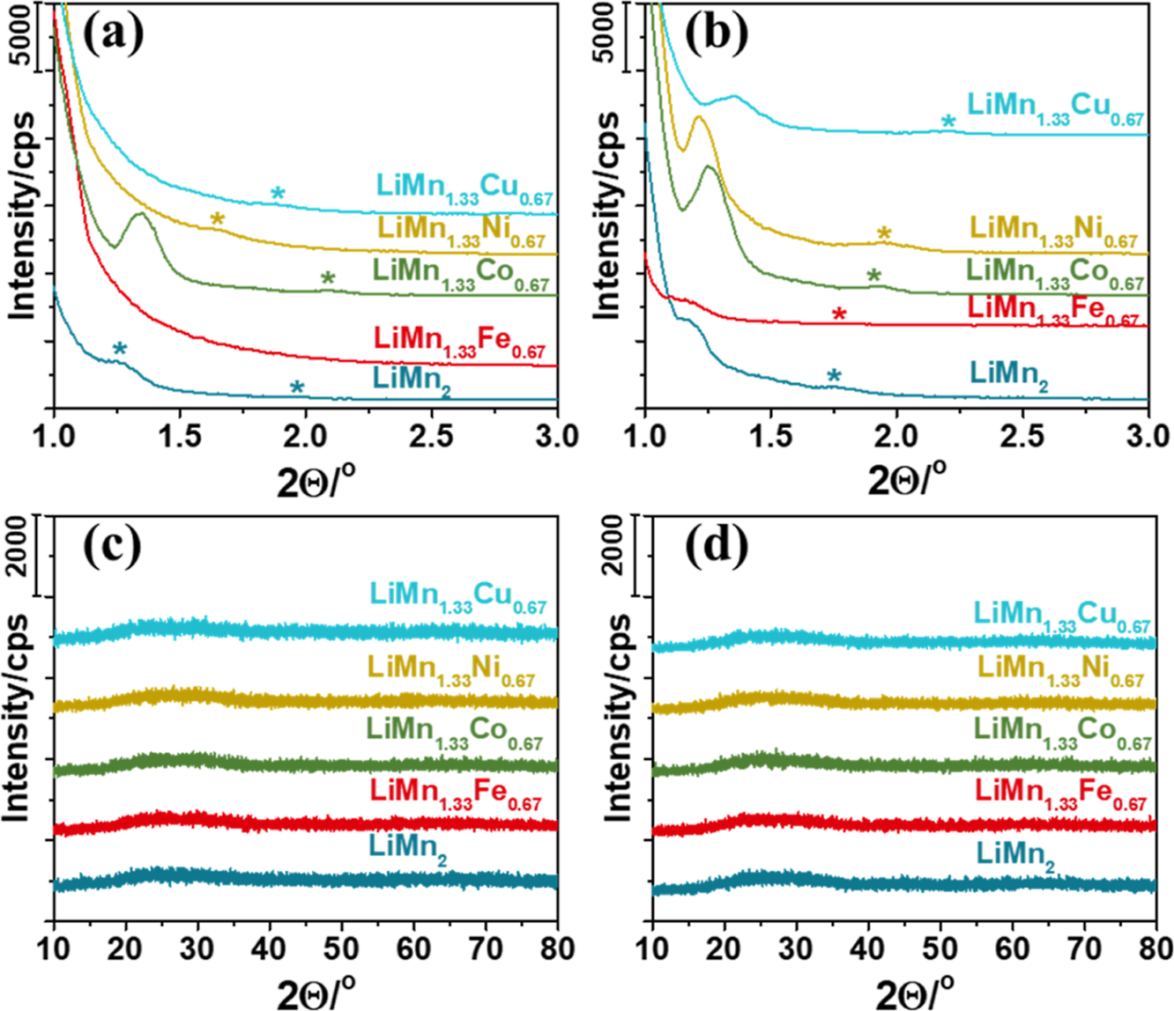
Small-angle XRD patterns of (a) fresh thin gel-mesophases and (b) an hour aged thick gel-mesophases (*line positions). Wide-angle XRD patterns of 3 h aged (c) thin and (d) thick gel-mesophases of the LLC-LiMn_2–*x*_M_*x*_ samples.

The gel films of all compositions (coated on glass slides or FTO) are analyzed by collecting their small- and wide-angle XRD patterns. The small-angle XRD patterns display diffraction line(s) between 1 and 5°, 2θ, indicating the formation of the LLC mesophase; see XRD patterns in Figures 1a,b and S1–S4. Note that the gel phase of the same composition, without the secondary metal salt, has been previously investigated in detail.^11^ The samples with a second metal salt also display similar diffraction patterns; the presence of a second metal salt in the LLC mesophase, if not improving the structure positively, has almost no effect on the structure in the mesoscale; see Figures S1–S4. Figure 1a,b displays the small-angle XRD patterns of a fresh and 1 h aged LLC mesophase of a LiNO_3_/[Mn(OH_2_)_4_](NO_3_)_2_/Fe(NO_3_)_2_·9H_2_O/CTAB/P123 (with a 20:26.7:13.3:1:1 mol ratio, respectively) composition (denoted as LLC-LiMn_1.3_Fe_0.67_), indicating the stability of the mesophase. The wide-angle XRD patterns display no diffraction line between 10 and 80°, 2θ, indicating no salt or surfactant crystallization from the mesophase and also indicating that the salt species are in their molten phase in the thin or thick LLC mesophases; see Figure 1c,d. Notice that the wide-angle patterns are collected from spin-coated (thin gel film) and drop-casted (thick gel) samples after 3 h of aging, also showing the high stability of the gel phases.

Notice that the drop-casted films also display more diffraction lines at small angles (around 1.29 to 1.55°, 2θ, range) with small shifts in the presence of different second salts, namely, Fe(III), Co(II), Ni(II), and Cu(II) nitrate salts. The second diffraction line is observed between 1.83 and 2.38°, 2θ, in these mesophases. Moreover, there is likely another strong line below 1°, 2θ, that cannot be measured by using our diffractometer. These lines correspond to (100), (200), and (300) planes, but it is still difficult to suggest a structure for the mesophases using these patterns.

Then, the gel films are calcined at 300 °C for 1–3 h, depending on the film thickness, and annealed at higher temperatures to obtain mesoporous thin films (denoted as *meso*-LiMn_2–*x*_M_*x*_O_4_). The *meso*-LiMn_2–*x*_M_*x*_O_4_ samples are collected from the glass slides and characterized by recoding their SEM and TEM images, XRD patterns, XPS spectra, and N_2_ adsorption–desorption isotherms.

### Characterization of the *meso*-LiMn_2–*x*_Fe_*x*_O_4_ Films

**Figure 2:**
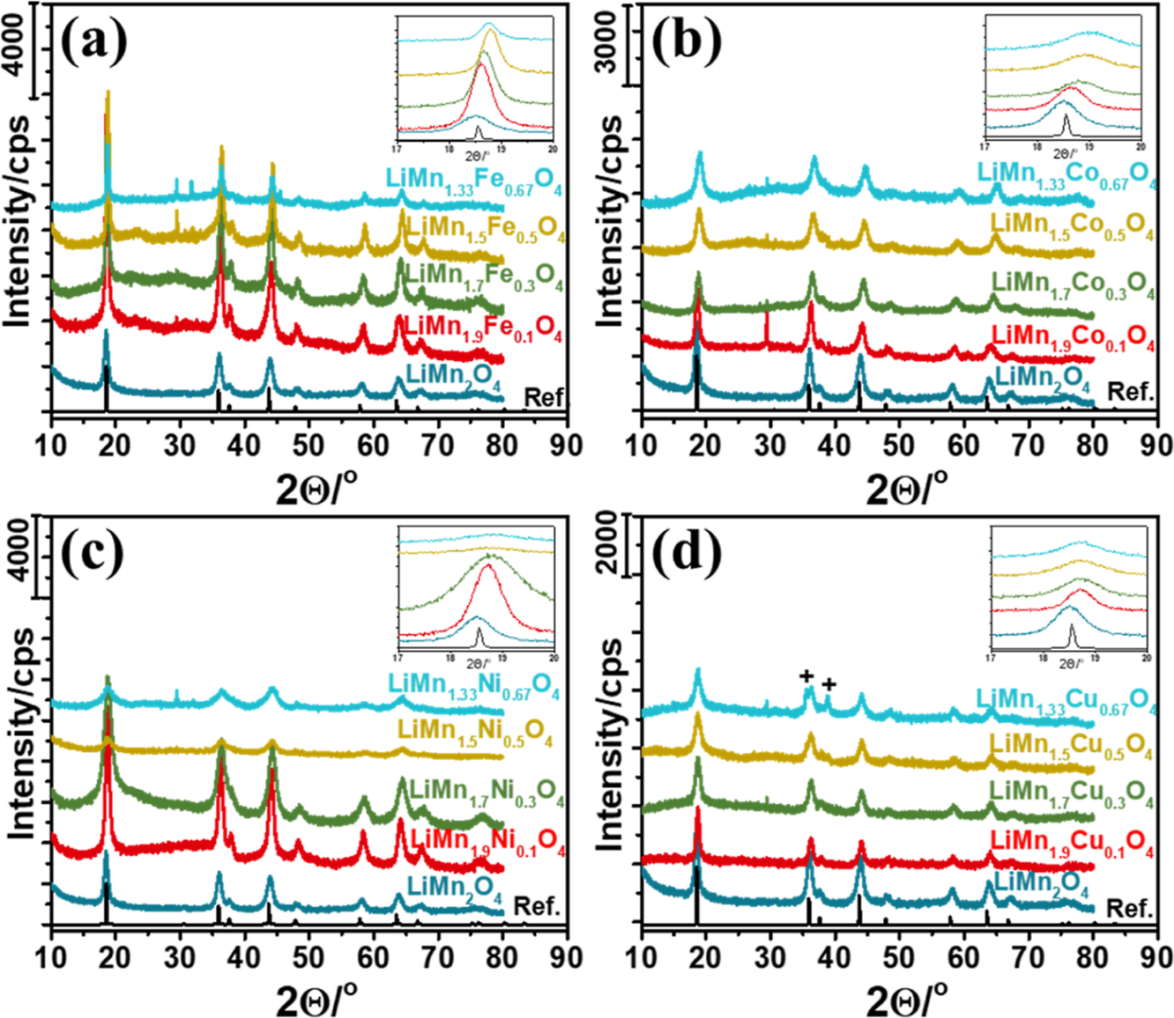
XRD patterns of the *meso*-LiMn_2–*x*_M_*x*_O_4_ thin films in the 10 to 80° and insets from 17 to 20°, 2θ (ref. is LiMn_2_O_4_-JCPDS no. 54-0252) and M is (a) Fe, (b) Co, (c) Ni, and (d) Cu (* CuO lines).

The wide-angle XRD patterns of *meso*-LiMn_2–*x*_Fe_*x*_O_4_ are shown in Figure 2a. The diffraction lines are indexed to cubic spinel LiMn_2_O_4_ structure using the reference data (JCPDS no. 54-0252). Additional weak-diffraction lines at 29.5 and 31.7°, 2θ, are observed in the patterns, corresponding to some impurities (like NaNO_3_ and FeCO_3_ crystals; see later). The NaNO_3_ crystals form by a reaction between the precursor mixture and glass substrate that contains a large amount (about 23%) of sodium. Also, the incorporation of iron into the LiMn_2_O_4_ structure is evident from the gradual shift of the main diffraction lines to higher angles due to the slight difference between the sizes of the Mn and Fe ions in the lattice.

XPS spectra of the *meso*-LiMn_2–*x*_Fe_*x*_O_4_ films are collected in the C 1s, O 1s, Mn 3s, 2p, and Fe 2p regions for surface analysis. The C 1s and the O 1s spectra are shown in Figure S5. Notice that an extra peak in the C 1s spectra appears and amplifies at 288.5 eV by increasing the iron content in the *meso*-LiMn_2–*x*_Fe_*x*_O_4_ films; see Figure S5a. This peak has been assigned to carbonate species and is also additional evidence for the formation of some carbonate species (FeCO_3_, as also predicted from the XRD patterns using JCPDS no. 29-0696) on the film surface. In the O 1s spectra, the lattice oxygen peak is observed at 529.8 eV with a shoulder at 531.4 eV due to hydroxides and carbonates. The intensity of the shoulder peak is enhanced with increasing iron amount in the structure and is attributed to iron carbonate species; see Figure S5b. The Mn 3s and 2p, as well as Fe 2p spectra, are also recorded and analyzed to elucidate the structural details of the surface species. Figure S6a–c shows the XPS spectra in the Mn 3s, Mn 2p, and Fe 2p regions, respectively. The Mn 3s spectrum (Figure S6a) of the LiMn_2_O_4_ sample displays two peaks with a peak-to-peak separation of 4.7 eV (splitting energy) due to 3s and valence unpaired electron coupling.^31^ A 4.7 eV splitting energy is observed from the Mn(III) and Mn(IV) species.^31^ The peak at 84.5 eV slightly shifts to a higher binding energy with increasing iron in the structure and causes a decrease in the splitting energy. The decrease in the splitting energy also means that the Mn oxidation state increases in the *meso*-LiMn_2–*x*_Fe_*x*_O_4_ films. It is likely that the Mn(III) sites are replaced by Fe(III) in the spinel structure, and the Mn is in a +4 oxidation state. This is also evident in the Mn 2p spectra (in the ^2^P_3/2_ spin–orbit state, in Figure S6b). The Mn 2p (^2^P_3/2_) spectrum of *meso*-LiMn_2_O_4_ displays a shoulder at 641 eV due to the Mn(II) sides. The intensity of the shoulder decreases with the integration of iron into the structure, together with the enhancement of the Mn(IV) region; compare the spectra in Figures S6b and S7; this is also consistent with the observed changes in the Mn 3s region. The Fe 2p spectra of the *meso*-LiMn_2–*x*_Fe_*x*_O_4_ samples are shown in Figure S6c. The peak at 710.8 eV due to Fe 2p (^2^P_3/2_) and a broad satellite peak at 719.3 eV are strong evidence of the presence of Fe(III) species in the structure. Notice also that the only change in the spectra with increasing iron is the enhancement of the peak intensities in the Fe 2p region. Thus, the oxidation state of iron is +3 in all *meso*-LiMn_2–*x*_Fe_*x*_O_4_ compositions.^31^

The top-view SEM images of the *meso*-LiMn_2–*x*_Fe_*x*_O_4_ films are collected directly using the FTO-coated films (denoted as F-LiMn_2–*x*_Fe_*x*_O_4_, where x is 0, 0.10, 0.30, 0.50, and 0.67); see Figure S8. The SEM image of the F-LiMn_2_O_4_ film displays uniform ultrasmall nanoparticles with a relatively smooth surface (Figure S8a). With the addition of 5% iron into the structure (F-LiMn_1.9_Fe_0.1_O_4_), the film becomes rougher but still shows porous features and ultrasmall particles; see Figure S8b. Notice that the other compositions of F-LiMn_2–*x*_Fe_*x*_O_4_ have similar views with some larger particles; see Figure S8c–e. However, the number of large particles is less in F-LiMn_1.33_Fe_0.67_O_4_ and does not correlate with the iron content in the structure.

### Characterization of the *meso*-LiMn_2–*x*_Co_*x*_O_4_ Films

The *meso*-LiMn_2–*x*_Co_*x*_O_4_ (where *x* = 0.10, 0.30, 0.50, and 0.67) samples are also characterized using the same techniques. Powder XRD patterns of the films, fabricated at 2000 rpm, are shown in Figure 2b. Like *meso*-LiMn_2–*x*_Fe_*x*_O_4_, all the *meso*-LiMn_2–*x*_Co_*x*_O_4_ samples have very similar diffraction patterns. The diffraction lines can be indexed to cubic spinel structure using the ICDD card (JCPDS no. 54-0252). The line at 29.5°, 2θ, is also observed in the powder patterns of the *meso*-LiMn_2–*x*_Co_*x*_O_4_ samples, indicating the formation of NaNO_3_ impurities. However, the diffraction line at 31.7°, 2θ (observed from the *meso*-LiMn_2–*x*_Fe_*x*_O_4_ samples), is absent in the *meso*-LiMn_2–*x*_Co_*x*_O_4_ samples. The diffraction lines shift to higher angles with increasing cobalt in the *meso*-LiMn_2–*x*_Co_*x*_O_4_ samples, also indicating that cobalt is successfully incorporated into the spinel structure without phase separation; see Figure 2b. Moreover, the diffraction lines become broader with increasing cobalt in the structure. The average crystallite size is evaluated using Scherrer’s equation and the diffraction line at around 18.6°, 2θ. The particles are 16.5, 16.8, 12.8, 9.4, and 8.6 nm in the *meso*-LiMn_2–*x*_Co_*x*_O_4_ samples with *x* values of 0, 0.10, 0.30, 0.50, and 0.67, respectively.

XPS spectra of the F-LiMn_2–*x*_Co_*x*_O_4_ thin films (fabricated at 5000 rpm on FTO) are collected in the C 1s, O 1s, Mn 3s and 2p, and Co 2p regions. The C 1s spectra differ from those of F-LiMn_2–*x*_Fe_*x*_O_4_ films in the carbonate region. The carbonate signal is absent in the spectra, as confirmed by the XRD. Figure S9 displays the spectra of the O 1s, Mn 3s, Mn 2p (^2^P_3/2_), and Co 2p (^2^P_3/2_) spectra. The lattice oxygen peak at 529.8 eV is used to normalize the O 1s spectra of the F-LiMn_2–*x*_Co_*x*_O_4_ films to compare the amount of other oxygen species on the electrode surfaces. Due to the hydroxyl feature, the shoulder peak at 531.2 eV is quite weak in all compositions with a similar intensity; see Figure S9a. Figure S9b shows the Mn 3s spectra of the F-LiMn_2–*x*_Co_*x*_O_4_ films. The splitting energy in the Mn 3s region decreases from 4.7 to 4.4 eV with increasing cobalt in the films, also indicating the increase in the Mn(IV) species in the samples. The change in the Mn 3s spectra may also be attributed to the homogeneous mixing of Co and Mn in the structure. The Mn 2p (^2^P_3/2_) spectra also correlate with the above proposal (Figures S9c and S10). The shoulder peak at 641 eV (due to the Mn(II) species) gradually loses its intensity with increasing cobalt in the structure. Also, the Co satellite peak at 790.3 eV is observed in the spectra (Figure S9d) and originates from the Co(III) species on the electrode surface.^31^

**Figure 3:**
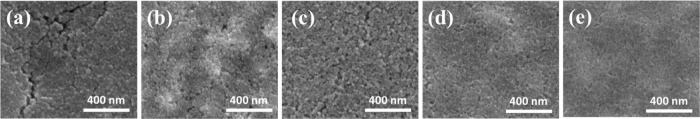
Top-view SEM images of the F-LiMn_2–*x*_Co_*x*_O_4_ films, where *x* is (a) 0.00, (b) 0.10, (c) 0.30, (d) 0.50, and (e) 0.67.

Film morphology of the F-LiMn_2–*x*_Co_*x*_O_4_ is also investigated by collecting a series of top-view SEM images; see Figure 3. Notice that the film quality, at all compositions, is better than that of the F-LiMn_2–*x*_Fe_*x*_O_4_ films; compare the images in Figures 3 and S8. Also, the particle size decreases with increasing cobalt in the composition, as already evidenced by broadening of the XRD line widths.

### Characterization of the *meso*-LiMn_2–*x*_Ni_*x*_O_4_ Films

The *meso*-LiMn_2–*x*_Ni_*x*_O_4_ thin films are characterized using the same techniques. Figure 2c displays a series of XRD patterns of the *meso*-LiMn_2–*x*_Ni_*x*_O_4_ powder samples collected from many films coated on microscope slides. The patterns are indexed to a cubic spinel LiMn_2_O_4_ structure (ICDD database, JCPDS no. 54-0252). The diffraction lines shift to higher angles and become broader with increasing nickel in the structure; see Figure 2c. The crystallite size also decreases with increasing nickel in the *meso*-LiMn_2–*x*_Ni_*x*_O_4_ samples, from 15.6 nm in the LiMn_1.9_Ni_0.1_O_4_ to 5.5 nm in the LiMn_1.33_Ni_0.67_O_4_ sample.

**Figure 4:**
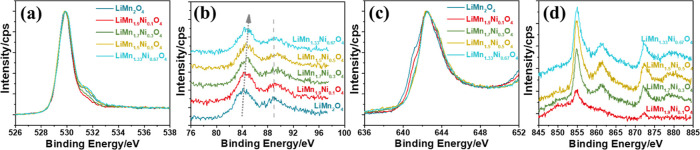
XPS spectra of *meso*-LiMn_2–*x*_Ni_*x*_O_4_ films in the (a) O 1s, (b) Mn 3s, (c) Mn 2p (^2^P_3/2_), and (d) Ni 2p regions.

XPS spectra of the *meso*-LiMn_2–*x*_Ni_*x*_O_4_ samples are also collected in the O 1s, Mn 3s, Mn 2p (^2^P_3/2_), and Ni 2p regions. Figure 4a shows the O 1s spectra of the *meso*-LiMn_2–*x*_Ni_*x*_O_4_ samples. According to the spectra, the shoulder (due to the hydroxyl groups) at 531.2 eV is weakly observed in the nickel samples. The splitting energy in the Mn 3s spectra decreases from 4.7 to 4.4 eV with increasing nickel, also indicating an increase of Mn(IV) species in the samples; see Figure 4b. Similarly, the Mn 2p (^2^P_3/2_) shoulder at 641 eV (due to Mn(II)) gradually decreases with increasing nickel amount in the *meso*-LiMn_2–*x*_Ni_*x*_O_4_ samples; see Figures 4c and S11. The Ni 2p spectrum is more complicated^32^ because the satellite peaks and their binding energies are very similar in both Ni(II) and Ni(III) in their oxide and hydroxide derivatives; see Figure 4d. Therefore, Ni 2p can be assigned based on the interpretation of the Mn 3s and 2p peaks, and the peak at 854.9 and 872.4 eV are due to ^2^P_3/2_ and ^2^P_1/2_ spin–orbit states, respectively, and corresponds to Ni(II)/Ni(III) species (see later in the electrochemistry section).

Figure S12 displays the SEM images of the F-LiMn_2–*x*_Ni_*x*_O_4_ films (top-view). The morphology and particle size are very similar in the SEM images of the F-LiMn_2_O_4_ and F-LiMn_1.9_Ni_0.1_O_4_ films; see Figure S12a,b. The particle size decreases with increasing nickel in the films; see Figure S12c–e, as also correlated in the XRD line-widths; the fwhm of the diffraction lines increase with increasing nickel content, indicating a decrease in the particle size; see Figure 2c.

### Characterization of the *meso*-LiMn_2–*x*_Cu_*x*_O_4_ Films

The *meso*-LiMn_2–*x*_Cu_*x*_O_4_ samples are also characterized using XRD, XPS, and SEM techniques. Figure 2d shows the XRD patterns. The XRD patterns of the *meso*-LiMn_1.9_Cu_0.1_O_4_ and *meso*-LiMn_1.7_Cu_0.3_O_4_ samples are indexed to the cubic spinel LiMn_2_O_4_ structure. However, further increasing copper results in phase separation, and new diffraction lines appear in the XRD pattern of the LiMn_1.33_Cu_0.67_O_4_ sample at 35.58, 38.84, and 61.53°, 2θ (originating from the CuO phase). The new lines can be indexed using the ICDD card, JCPDS no. 45-093. The phase separation is not surprising because the highest stable oxidation state of copper is +2; therefore, even if all of the Mn species are in a +4 oxidation state, the charge balance in the lattice of the spinel LiMn_1.33_Cu_0.67_O_4_ structure cannot be satisfied. Therefore, the LiMn_1.33_Cu_0.67_O_4_ sample cannot be pure; it is rather a mixture of LiMn_2–*x*_Cu_*x*_O_4_ and CuO.

XPS spectra are also collected for further characterization of the F-LiMn_2–*x*_Cu_*x*_O_4_ films. Figure S13a shows the O 1s spectra of the F-LiMn_2_O_4_, F-LiMn_1.7_Cu_0.3_O_4_, and F-LiMn_1.33_Cu_0.67_O_4_ films. The shoulder at 531.2 eV gains intensity with increasing copper in the F-LiMn_2–*x*_Cu_*x*_O_4_ films and originates from the hydroxyl sides on the film surface. Figures S13b and S14 show the same samples’ Mn 2p (^2^P_3/2_) spectra. Like the other *meso*-LiMn_2–*x*_M_*x*_O_4_ films, the shoulder at 641 eV (from the Mn(II) species) loses its intensity with increasing copper. Therefore, the Mn(IV) content in the structure increases with increasing copper in the samples, as also evidenced by the Mn 2p spectrum; notice that the spectrum is very similar to the spectrum of MnO_2_ in the LiMn_1.33_Cu_0.67_O_4_ sample. Figure S13c shows the Cu 2p spectra of the F-LiMn_1.7_Cu_0.3_O_4_ and F-LiMn_1.33_Cu_0.67_O_4_ films. The satellite peaks at 941 and 943.5 eV are characteristic of CuO,^33^ also evidenced by its formation in the XRD patterns. Additionally, the Cu 2p region peaks overlap with the Mn L_2_M_23_M_23_ and L_3_M_23_M_23_ peaks. The weak peaks at 931 and 946 eV (marked with asterisks) are due to the L_2_M_23_M_23_ and L_3_M_23_M_23_ features, respectively.^34^

For the morphological characterization, the SEM images of the F-LiMn_2–*x*_Cu_*x*_O_4_ films are also recorded and shown in Figure S15. The SEM images are similar to those of the F-LiMn_2–*x*_Fe_*x*_O_4_ samples. The large particles observed in the images are likely due to the formation of the CuO particles, also indicating a phase separation to LiMn_2–*x*_Cu_*x*_O_4_ and CuO.

### Comparison of the *meso*-LiMn_1.33_M_0.67_O_4_ Films

The LiMn_1.33_M_0.67_O_4_ composition is unique for the electrochemical properties and electrocatalysis of the OER. Therefore, these samples are analyzed in more detail in terms of phase separation and impurities. XRD patterns of the *meso*-LiMn_1.33_M_0.67_O_4_ samples are compared in Figure S16a to clarify the origin of the diffraction line at 29.4°, 2θ. Notice that this line appears at the same angle in all samples, independent of the type and amount of the second metal in the structure. Thus, it cannot be related to a second metal oxide. More importantly, an XRD database search gives a good match with the XRD pattern of NaNO_3_ crystals. These samples are calcined at 300 °C over the glass microscope slides, containing about 23% Na(I). It is likely that the nitrate ions of the precursors and Na(I) of the glass react to form NaNO_3_ crystals. Moreover, the NaNO_3_ crystals are stable at 300 °C and decompose over 350 °C.^35^ Therefore, it is reasonable to assign the line at 29.4°, 2θ, to NaNO_3_. Another sharp diffraction line at 31.7°, 2θ,^36^ in the LiMn_1.33_Fe_0.67_O_4_ composition may originate from the FeCO_3_ particles in the iron samples, as evidenced by the XPS data. Notice also that the diffraction lines of the *meso*-LiMn_1.33_Ni_0.67_O_4_ sample are very broad, likely due to either small crystallite size or overlapping of these lines with the lines NiO and Mn_3_O_4_ nanoparticles, and may indicate a phase separation. The diffraction line at 31.9°, 2θ, has been attributed to nanocrystalline Mn_3_O_4_ in the powder. The type of impurities in the *meso*-LiMn_2–*x*_M_*x*_O_4_ samples is previously discussed in the X-ray Photoelectron Spectroscopy section for each metal system. To further clarify these findings and conclude this section, the XPS survey spectra of the LiMn_1.5_Co_0.5_O_4_ sample prepared on glass microscope slides and FTO surfaces are compared in Figure S16b. The survey spectrum of the sample (prepared over a glass slide) displays Na 1s and N 1s peaks. However, the same sample over the FTO surface has no Na and N signals in the survey spectrum; see Figure S16b. The FTO layer on a glass prevents the diffusion of sodium ions into the films. Therefore, these findings and XRD analysis undoubtedly prove the formation of NaNO_3_ impurities.

**Figure 5:**
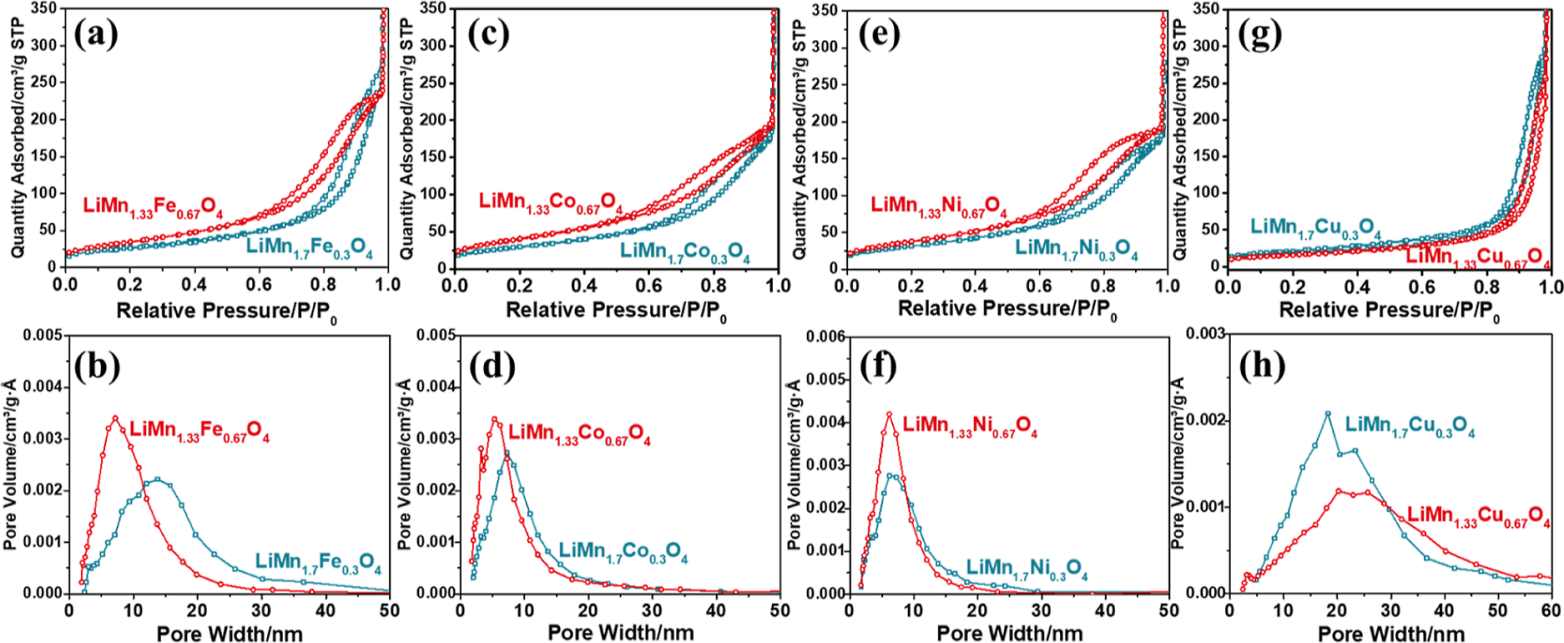
N_2_ (77 K) adsorption–desorption isotherms and pore-size distribution plots of the (a,b) LiMn_1.7_Fe_0.3_O_4_ and LiMn_1.33_Fe_0.67_O_4_, (c,d) LiMn_1.7_Co_0.3_O_4_ and LiMn_1.33_Co_0.67_O_4_, (e,f) LiMn_1.7_Ni_0.3_O_4_ and LiMn_1.33_Ni_0.67_O_4_, and (g,h) LiMn_1.7_Cu_0.3_O_4_ and LiMn_1.33_Cu_0.67_O_4_ samples, respectively.

N_2_ adsorption–desorption isotherms are also collected from a selected group of drop-casted samples, namely, LiMn_2_O_4_, LiMn_1.7_M_0.3_O_4_, and LiMn_1.33_M_0.67_O_4_ and displayed in Figure 5. Table 1 tabulates the N_2_ adsorption–desorption related data. The isotherms display type IV hysteresis, characteristic to the mesoporous materials. Increasing M in all samples, except Cu, increases the surface area and decreases the pore size in the films. The pore-size variation from LiMn_1.7_M_0.3_O_4_ to LiMn_1.33_M_0.67_O_4_ (where M is Fe, Co, and Ni) samples follows a M-dependent order (Ni < Co < Fe, compare Figure 5b,d,f), which is the opposite of the water content of their mesophases. It seems like the number of waters in the LLC-LiMn_2–*x*_M_*x*_-P123 mesophase has a vital role in the pore-size and pore-size distribution, as well as the film thickness (for the role of water, see the electrochemistry sections). Moreover, the highest surface area is recorded from the LiMn_1.33_Co_0.67_O_4_ and LiMn_1.33_Ni_0.67_O_4_ samples; these samples also perform well in the OER (see later). However, increasing the copper content in the LiMn_2–*x*_Cu_*x*_O_4_ samples has an opposite effect (Figure 5h); the pores become more disordered. Increasing copper also causes a phase separation and CuO particles form in the samples (as evidenced by XRD, SEM, and electrochemical measurements; see later).

**Table 1:** N_2_ Adsorption–Desorption Data of the *meso*-LiMn_2–*x*_M_*x*_O_4_ Thick Films

LiMn_2–*x*_M_*x*_O_4_	BET SA/m^2^/g	BJH PS/nm	PV/cm^3^/g
LiMn_2_O_4_	98	11.5	0.26
LiMn_1.7_Fe_0.3_O_4_	99	12.9	0.62
LiMn_1.33_Fe_0.67_O_4_	126	7.2	0.42
LiMn_1.7_Co_0.3_O_4_	116	7.2	0.42
LiMn_1.33_Co_0.67_O_4_	146	5.3	0.35
LiMn_1.7_Ni_0.3_O_4_	112	7.2	0.29
LiMn_1.33_Ni_0.67_O_4_	136	6.1	0.37
LiMn_1.7_Cu_0.3_O_4_	75	19.6	0.56
LiMn_1.33_Cu_0.67_O_4_	57	22.9	0.62

### Electrochemical Properties of the F-LiMn_2–*x*_M_*x*_O_4_ Electrodes

The *meso*-LiMn_2–*x*_M_*x*_O_4_ films are coated over a 1 cm^2^ FTO substrate by spin coating the mother liquors at 5000 rpm and calcined at 300 °C for an hour (denoted as F-LiMn_2–*x*_M_*x*_O_4_). These electrodes have the best quality regarding their surface roughness and stability in electrochemical tests and are the best-performing electrodes in OER. Therefore, these electrodes are used in all electrochemical investigations, namely, for scan rate-dependent CVs (from 2 to 20 mV/s) in 1 M LiNO_3_ electrolyte solution and 300 CVs (0 to 1 V), CA experiments for Tafel slope analysis, and CP experiments at various current densities (from 1 to 50 mA/cm^2^) in 1 M KOH electrolyte solution.

### Electrochemical Characterization of the F-LiMn_2–*x*_Fe_*x*_O_4_ Electrodes

The electrochemical properties of the F-LiMn_2–*x*_Fe_*x*_O_4_ electrodes are investigated by first collecting CVs in a 1 M LiNO_3_ electrolyte to show lithium–ion intercalation and deintercalation processes. The CVs are recorded from 0.3 to 1.3 V (vs NHE) with a scan rate of 2 to 20 mV/s with 2 mV/s increments using a three-electrode cell (FTO-coated electrode as the WE, Pt wire as the CE, and Ag/AgCl (3.5 M KCl) as the RE). Figure S17 displays the scan rate-dependent CVs of the F-LiMn_2–*x*_Fe_*x*_O_4_ (*x* = 0, 0.10, 0.30, 0.50, and 0.67) electrodes. The F-LiMn_2_O_4_ electrode displays the highest charge capacity under the lithium–ion intercalation and deintercalation reaction curves (Figure S17a) in the 0.3 to 1.3 V (vs NHE) potential window. The charge capacity gradually decreases with increasing iron in the F-LiMn_2–*x*_Fe_*x*_O_4_ electrodes (Figures S17b–d and 6a), such that the F-LiMn_1.33_Fe_0.67_O_4_ electrode has the weakest oxidation/reduction peaks due to the lithium–ion deintercalation and intercalation processes. Figure S17f compares the CVs of all compositions at a scan rate of 20 mV/s. Note that the lithium–ion deintercalation and intercalation processes occur in two steps. Half of the lithium ion in the F-LiMn_2_O_4_ electrode is deintercalated to F–Li_0.5_Mn_2_O_4_ at 0.9 V, and the other half at 1.1 V to form λ-MnO_2_ phase.^11^ The number of Mn(III) sides decreases with increasing iron in the F-LiMn_2–*x*_Fe_*x*_O_4_ electrodes and causes a decrease in the lithium–ion deintercalation. The Fe(III) oxidation likely occurs at more positive potentials to fully deintercalate the lithium ions from the *meso*-LiMn_2–*x*_Fe_*x*_O_4_ structure. Note also that the charge capacity under the first peak at 0.9 V is higher than the second one at 1.1 V, but the rate of decay in the second peak is more compared to the first one with increasing iron in the F-LiMn_2–*x*_Fe_*x*_O_4_ electrodes. Note that the lithium–ion deintercalation due to the oxidation of the Fe(III) sides requires more positive potentials. Additionally, the total charge capacity under these peaks decreases with the addition of a second M(III) ion in the *meso*-LiMn_2–*x*_M_*x*_O_4_ structure (where M is Fe, Co, Ni, and Cu). Because the oxidation of Fe(III), Co(III), and Ni(III) to their +4 oxidation states occurs at relatively more positive potentials due to their higher electronegativities.^37,38^

**Figure 6:**
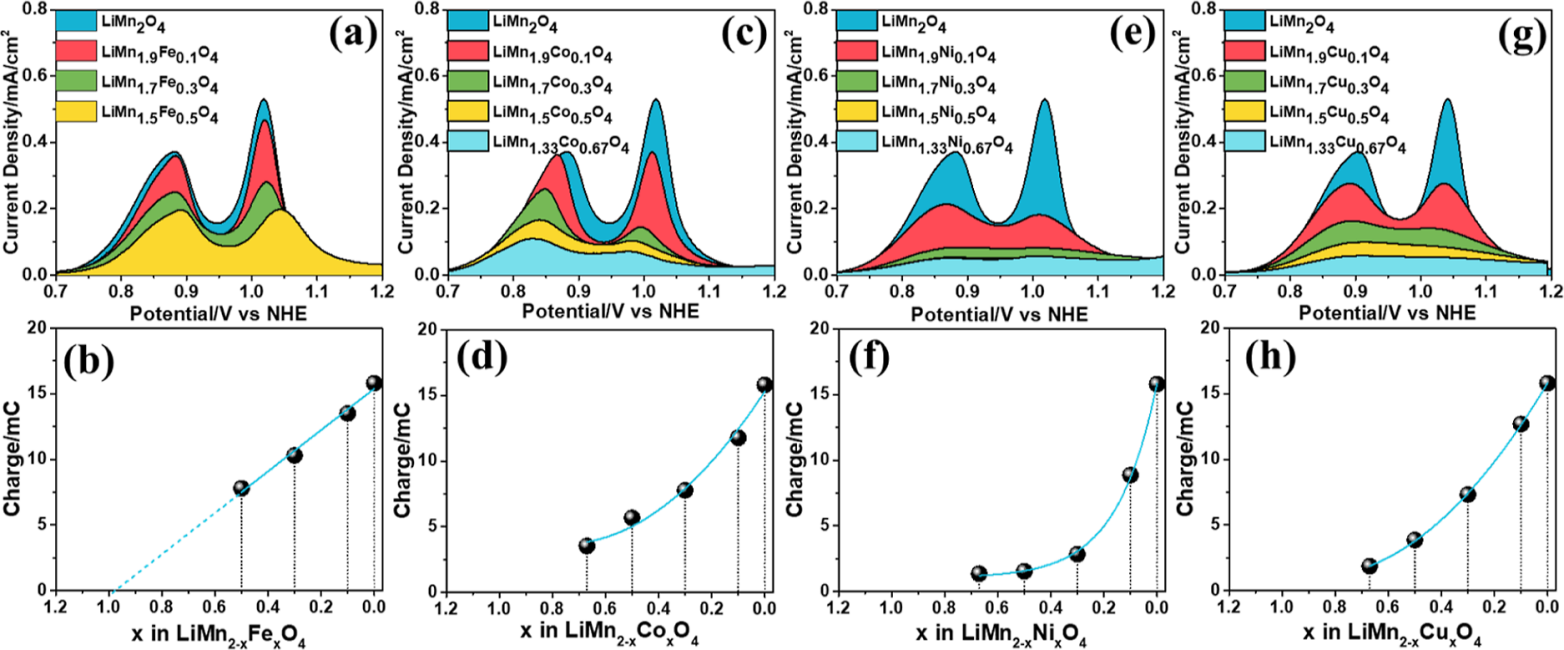
Area under the *I*–*V* curves of LiMn_2–*x*_M_*x*_O_4_ electrodes (where M is (a) Fe, (c) Co, (e) Ni, and (g) Cu) used for the calculation of charge capacity and plot of charge capacity vs Mn % in the LiMn_2–*x*_M_*x*_O_4_ electrodes (where M is (b) Fe, (d) Co, (f) Ni, and (h) Cu).

The LSVs between 0.6 and 1.2 V are recorded at a 5 mV/s scan rate to evaluate the charge-capacity of each electrode using the area under the oxidation peaks; see Figure 6a. Then, the charge-capacity versus Mn percentage is plotted (Figure 6b). As expected, the plot fits a linear equation with an intercept at 50% Mn. It does not mean it is “possible” to prepare an F-LiMnFeO_4_ electrode, but it means that while the iron is in a +3 oxidation state, the Mn in a +4 oxidation state to charge-balance the spinel structure, and Fe(III) is homogeneously distributed into the structure in the investigated composition range.

The F-LiMn_2–*x*_Fe_*x*_O_4_ electrodes are further used to investigate the Mn(VI)-DR (see eq 1) and electrode stability to elucidate the role of iron. The reaction in eq 1 has been extensively investigated using a F-LiMn_2_O_4_ electrode.^11^ Briefly, Mn(II) oxidizes to a +6 oxidation state during the OER. Three Mn(VI) sides (linear or broken cube) are electrochemically not equivalent, unstable, and undergo to a Mn(VI)-DR, as depicted in eq 1. These Mn(VI) species have different numbers of oxygen and oxygen connectivity that makes one (in the middle) of the Mn(VI) sides more electronegative, as calculated from Sanderson’s geometric mean of the electronegativity.^19,37,38^ The more electronegative Mn(VI) gets reduced to Mn(IV) (and deposits as MnO_2_), and the less electronegative Mn(VI) sides get oxidized to Mn(VII) and leach out from the electrode surface (as permanganate ions to the electrolyte solution, see ref (11) for details).

First, 300 CVs are collected between 0 and 1 V at a 50 mV/s scan rate in 1 M KOH solution. The trend in the CVs shows that the F-LiMn_2–*x*_Fe_*x*_O_4_ electrodes are also unstable, similar to the F-LiMn_2_O_4_ electrode. The current density gradually decays in the OER region with increasing CV cycling; see Figure S18a–e. The decay in the current density with cycling is attributed to a Mn(VI)-DR (eq 1) and occurs in all compositions. Figure S18f displays the plots of the current density at 1 V versus CV cycle number and provides information about the Mn(VI)-DR behavior; the plot revials that iron cannot stabilize the F-LiMn_2–*x*_Fe_*x*_O_4_ electrodes. Moreover, iron also undergoes its own DR and may not be a good candidate to stabilize Mn-based electrodes for effective OER electrocatalysis. The Fe(V) species either undergo a DR (from Fe(V) to Fe(VI), namely, [FeO_4_]^2–^ and Fe(III), namely, FeOOH, see eq 2) or further oxidation to produce soluble [FeO_4_]^2–^ species^39^ (see eq 3) and also cause electrode degradation
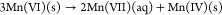
1
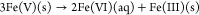
2
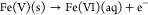
3

The used F-LiMn_2–*x*_Fe_*x*_O_4_ electrodes (after 300 CV measurements) are further analyzed using the XPS technique to shine some light on the above processes. The O 1s spectra of the cycled electrodes are shown in Figure S19a. Notice that the shoulder at 531.2 eV (assigned to carbonate species) disappears after 300 CVs in a 1 M KOH solution. Moreover, the peak at 288.8 eV (assigned to carbonates) in the C 1s spectra of the F-LiMn_2–*x*_Fe_*x*_O_4_ electrodes also completely disappears after cycling. This is not surprising because the electrodes are used for water oxidation in the cycling process. In the suggested OER mechanism, the hydroxide source that attacks the metal oxo bond to form M–O–OH is the water self-dissociation and produces both OH^–^ and H_3_O^+^ ions (2H_2_O ↔ OH^–^ + H_3_O^+^). Electrocatalytic water oxidation uses the hydroxide ion, leaving a H_3_O^+^-rich electrode surface that causes iron carbonate dissociation. This observation is very valuable and supports the water oxidation mechanism suggested in our previous studies.^20−22^

XPS spectra of the F-LiMn_2–*x*_Fe_*x*_O_4_ electrodes in the K 2p region are also recorded after collecting 300 CVs; see Figure S19b. Cycling in 1 M KOH may cause a potassium-ion intercalation into the structure. Notice also that the intensity of the K 2p peak decreases with increasing iron in the LiMn_2–*x*_Fe_*x*_O_4_ electrodes and disappears in the spectrum of the F-LiMn_1.33_Fe_0.67_O_4_ electrode. A control experiment is also designed to ensure the effectiveness of the washing and drying procedure of the electrodes after cycling experiments. The total time for the 300 CV cycles is about 4 h; therefore, a freshly prepared F-LiMn_2_O_4_ electrode is also kept for 4 h in 1 M KOH solution and then removed from the electrolyte solution, washed, and dried like the used electrode, and its K 2p spectrum is recorded. The K 2p spectrum displays no signal; see Figure S19c. This means that the washing method is successful in eliminating all electrolytic residues. Thus, the detected K species in the used electrode must be the intercalated potassium ions in the electrodes/electrode surface.

The LiMn_2–*x*_Fe_*x*_O_4_ electrodes are further characterized by recording their XPS spectra in the Mn 3s, Mn 2p, and Fe 2p regions. Figure S20a displays the Mn 3s spectra. The splitting energy between the two peaks in the Mn 3s region is 4.4 eV for all compositions. This means that the Mn species are in a +4 oxidation state on the electrode surface after 300 CV cycles. This is also confirmed by the Mn 2p spectra, which display a typical peak shape of MnO_2_; see Figure S20b. Moreover, the Fe 2p spectra display predominately Fe(III) signals, as evidenced by the position of the satellite peak at 719.3 eV (Figure S20c) and consistent with the Fe(V)-DR product (eq 2). There are two paths to oxidize iron to higher oxidation states. The first path is an electrochemical oxidation of surface iron species that react with water and are quickly reduced back to Fe(III), and the second one is the oxidation of Fe(III) to Fe(V) that undergoes a Fe(V)-DR to produce stable Fe(III) oxides and soluble [FeO_4_]^2–^ ionic species and dissociates into the electrolyte solution; see eq 2.

The extent of Fe(V)-DR has been evaluated by analyzing the Mn content from the XPS survey spectra of the used and unused electrodes. The Mn percentage of the coating solutions versus the surface Mn percentage (obtained from the XPS survey analysis) are plotted for both used and unused electrodes; see Figure S21. The plots show that the Mn amount decreases on the electrode surface with increasing Fe in the electrodes. The drop is slightly more pronounced in the used iron-rich electrodes, indicating that the Mn(VI)-DR occurs more than that of the Fe(V)-DR in the iron-rich electrodes. However, the other electrodes (before and after 300 CV measurements) follow a linear trend regarding Mn percentage. It means that both [MnO_4_]^−^ and [FeO_4_]^2–^ are dispersed into electrolytes through their DRs to a similar extent, and the electrode composition is preserved in terms of iron and Mn amounts.

### Electrochemical Characterization of the F-LiMn_2–*x*_Co_*x*_O_4_ Electrodes

**Figure 7:**
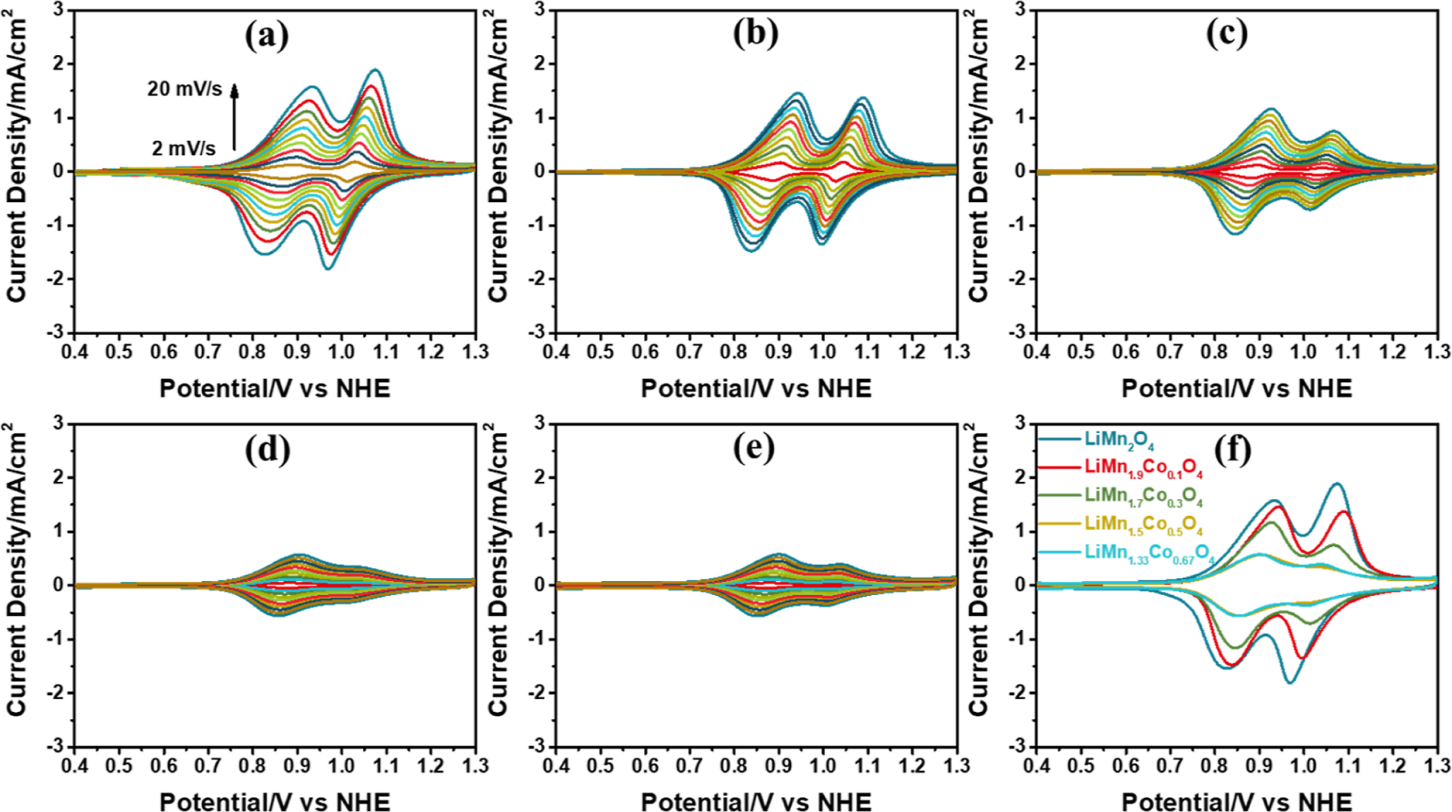
Scan rate-dependent (2 to 20 mV/s with an increment of 2 mV/s) CV curves of F-LiMn_2–*x*_Co_*x*_O_4_ electrodes in 1 M LiNO_3_ electrolyte solution, where *x* is (a) 0, (b) 0.1, (c) 0.3, (d) 0.5, (e) 0.67, and (f) CV curves of the F-LiMn_2–*x*_Co_*x*_O_4_ electrode at 20 mV/s scan rate.

Four F-LiMn_2–*x*_Co_*x*_O_4_ electrodes (*x* = 0.1, 0.3, 0.5, and 0.67) are prepared on FTO and characterized by electrochemical methods. First, the electrodes are used to collect a series of scan rate-dependent CVs between 0.3 and 1.3 V from 2 to 20 mV/s with an increment of 2 mV/s in 1 M LiNO_3_ electrolyte solution; see Figure 7a–e. The CVs show two distinct Mn(III) sites that oxidize to Mn(IV) at slightly different potentials, namely, at 0.9 and 1.1 V. Both redox peak current densities decay with increasing Co amount in the samples, but the decay is more pronounced in the second peak at 1.1 V, showing that the Co(III) is preferentially occupying those sides.

The redox peaks are due to Mn oxidation/reduction reactions. The cobalt redox must occur at relatively more positive potentials. The CVs (collected at a 20 mV/s scan rate) of all compositions are also shown in Figure 7f to compare the electrodes. Relative current densities at 0.9 and 1.1 V show the same trend as those of the F-LiMn_2–*x*_Fe_*x*_O_4_ electrodes. Notice also that the current densities under the lithium deintercalation/intercalation curves of the F-LiMn_1.5_Co_0.5_O_4_ and F-LiMn_1.33_Co_0.67_O_4_ electrodes are very similar. The total charge capacity under each curve is calculated from the LSVs (collected between 0.6 and 1.2 V with a 5 mV/s scan rate) to shine some light on the above behaviors; see Figure 6c. The area under the Mn(III) oxidation peaks (charge capacity) versus theoretical Mn % is plotted (Figure 6d). The charge capacity decays exponentially with increasing Co % in the LiMn_2–*x*_Co_*x*_O_4_ electrodes, slightly different from the case for the F-LiMn_2–*x*_Fe_*x*_O_4_ electrodes. This means the surface composition of the LiMn_2–*x*_Co_*x*_O_4_ electrodes does not alter over 25% Co and excess cobalt ions must diffuse into the interior of the particles in the LiMn_1.33_Co_0.67_O_4_ electrode.

**Figure 8:**
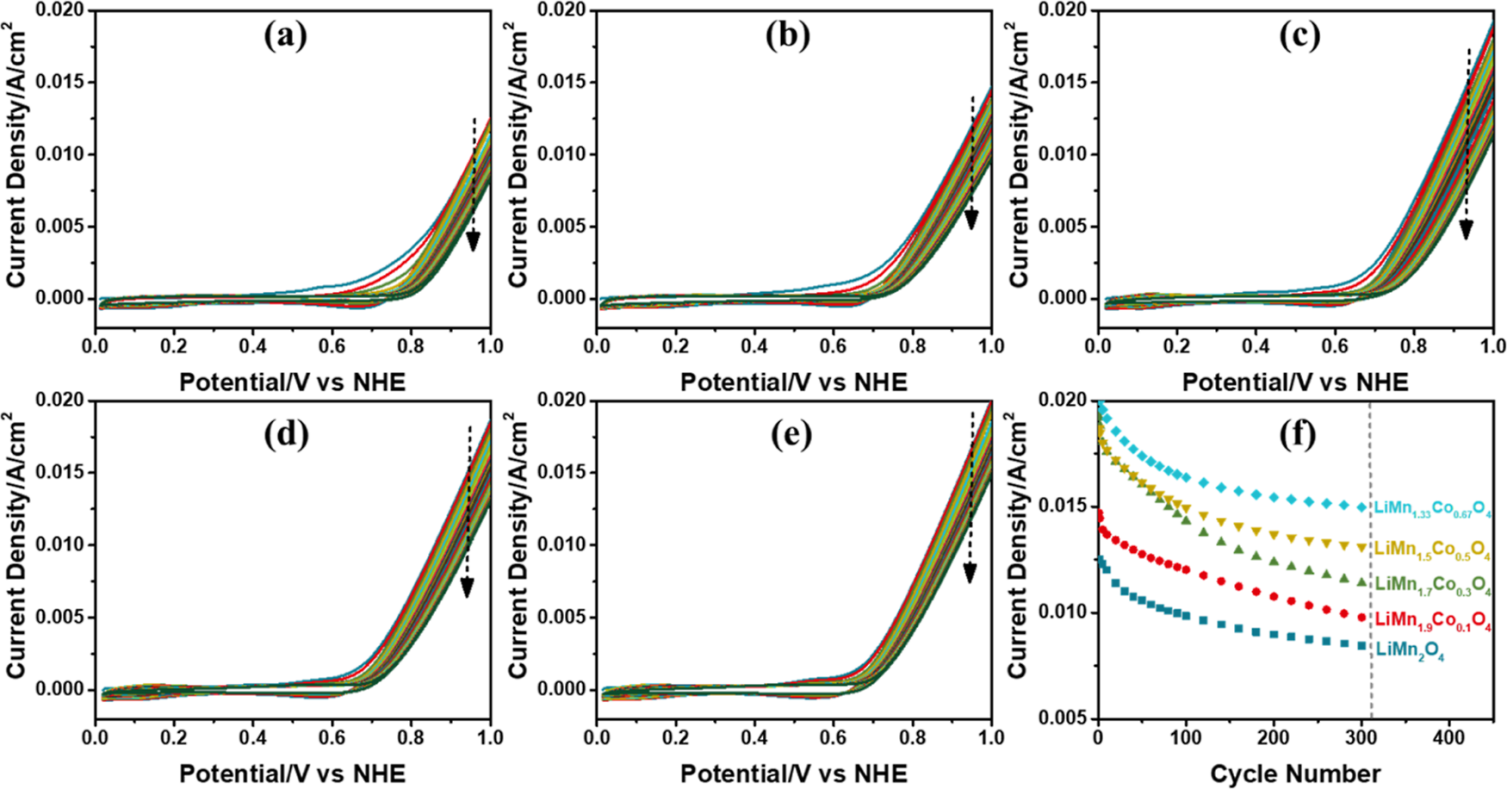
300 CV curves of the F-LiMn_2–*x*_Co_*x*_O_4_ electrodes in 1 M KOH solution with 50 mV/s sweep rate, where *x* is (a) 0, (b) 0.1, (c) 0.3, (d) 0.5, (e) 0.67, and (f) CV cycle number vs current density (at 1 V) plot.

The LiMn_2–*x*_Co_*x*_O_4_ electrodes are also used to collect 300 CVs between 0 and 1 V (vs NHE) at 50 mV/s scan rate in 1 M KOH solution to investigate the stability of the electrodes, see Figure 8a–e. Figure 8f shows the plots of the current density at 1 V (vs NHE) versus cycle numbers; the current density increases with an increase in Co in the electrodes. The OER efficiency is low in the F-LiMn_2_O_4_ and F-LiMn_1.9_Co_0.1_O_4_ electrodes, but the other electrodes (containing more than 15% cobalt) display a fast decay in the current density in the first 100 cycles. This behavior is attributed to electrochemical surface alteration due to Mn(VI)-DR. However, the electrode becomes more stable against Mn(VI)-DR in cobalt-rich electrodes after 200 CVs. In particular, the LiMn_1.33_Co_0.67_O_4_ composition shows better stability during the cycling process.

Similarly, the electrodes used are also analyzed by recording their XPS spectra. Figure S22a shows the O 1s spectra of all F-LiMn_2–*x*_Co_*x*_O_4_ electrodes. The shoulder at 531.2 eV due to surface hydroxyl groups disappears after 300 CVs. Because the electrode surface is converted to Mn_(1–*x*/2)_Co_*x*/2_O_2_ at 1 V, the potassium-ion contamination in the used LiMn_2–*x*_Co_*x*_O_4_ electrodes is also analyzed by recording the K 2p spectra; see Figure S22b. All used electrodes contain some potassium after 300 CV cycles, even if the cycling is terminated at more positive potentials. Thus, the intercalated potassium ions may be only partially removed at the end of the CV cycles.

The Mn 3s, Mn 2p, and Co 2p spectra are also recorded to identify the oxidation states of the surface species. The splitting energy is 4.4 eV in the Mn 3s spectra (Figure S23a) in all compositions and originates from the Mn(IV) species on the electrode surface. This is also supported by the Mn 2p (^2^P_3/2_) spectra; the Mn ^2^P_3/2_ peak shape is very similar to that of MnO_2_, as seen in Figure S23b. The satellite peak at 790 eV in the Co 2p spectra has been assigned to Co(III) species^31^ that occupy the Mn(III) sites in the spinel structure, see Figure S23c, and is consistent with the Mn 3s and 2p spectra.

XPS survey spectra are also recorded to analyze the atomic percentages of Mn and Co before and after 300 CVs, and the Mn % is marked by blue squares and red dots, respectively, in Figure S24. The surface Mn % of the electrodes is higher and may be attributed to an accumulation of Mn species on electrode surfaces and is also consistent with the decay in current density in first 100 CVs. The surface Mn percentage is reduced after 300 CVs in 1 M KOH; see red dots in Figure S24, likely due to a Mn(VI)-DR and the dissociation of some Mn species as permanganate ions during the CV measurements. 300 CVs also ensure an optimized Mn/Co ratio on the electrode surface and produce an efficient OER electrocatalyst.

### Electrochemical Characterization of the Mesoporous F-LiMn_2–*x*_Ni_*x*_O_4_ Electrodes

Mesoporous F-LiMn_2–*x*_Ni_*x*_O_4_ electrodes are also used to collect scan rate-dependent CVs in 1 M LiNO_3_ electrolyte solution to show deintercalation/intercalation redox behaviors; see Figure S25. As shown in the CVs, the lithium–ion deintercalation and intercalation peak currents decrease by increasing nickel in the F-LiMn_2–*x*_Ni_*x*_O_4_ electrodes. The oxidation/reduction peaks are visible in the CVs of the F-LiMn_1.9_Ni_0.1_O_4_ electrode; see Figure S25b. However, the electrodes with a higher nickel amount (above 15%) display very similar CVs, such as CVs of the F-LiMn_1.7_Ni_0.3_O_4_ electrode, which display a highly diminished lithium–ion deintercalation peak between 0.8 and 1.1 V; see Figure S25c. The electrodes with 25% or higher nickel compositions have almost the same voltammograms; compare CVs in Figure S25.

The F-LiMn_2–*x*_Ni_*x*_O_4_ electrodes are also swept from 0.6 to 1.2 V with a scan rate of 5 mV/s, and the charge capacities under the LSV curves are calculated and plotted against Mn percentages; see Figure 6e,f, respectively. Notice that when 5% nickel is incorporated into the spinel structure, the lithium deintercalation peak current is decreased more than in the other metal systems. As explained before, the electronegativities of these metals have significant effects on the oxidation of Mn(III) species and indirectly on the lithium deintercalation process. The decay of lithium–ion deintercalation is much sharper in the nickel case (Figure 6f). However, it almost stops when nickel reaches 25% in the F-LiMn_1.5_Ni_0.5_O_4_ electrode. The origin of this behavior will be discussed later.

The F-LiMn_2–*x*_Ni_*x*_O_4_ electrodes are further investigated to elucidate the effect of nickel on the Mn(VI)-DR. The 300 CVs are recorded at a 50 mV/s scan rate in 1 M KOH electrolyte solution and found that the catalytic performance of the F-LiMn_2–*x*_Ni_*x*_O_4_ electrodes increases with increasing nickel in the electrodes, observed as a current density increase in the water oxidation potentials; see Figure S26. The F-LiMn_1.33_Ni_0.67_O_4_ electrode displays the highest catalytic performance among all of the electrodes. Specifically, the F-LiMn_1.5_Ni_0.5_O_4_ and F-LiMn_1.33_Ni_0.67_O_4_ electrodes display another redox couple at around 0.45 V (vs NHE). The new redox peak currents gradually increase with increasing CV cycling. The new peaks are attributed to the nickel species formed on the electrode surface. Note that NiO transforms to Ni(OH)_2_ during CV cycling through oxidation to NiOOH, which has a layered structure that cannot be converted back to rock-salt NiO in the reverse cycle.^28^ Oxidation of NiO to NiOOH and its reduction to Ni(OH)_2_ is the origin of the layered Ni(OH)_2_ accumulation on the electrode surface during CV cycling.^28^ Therefore, the new redox peaks at high nickel concentrations may be attributed to NiO or nickel-rich LiMn_2–*x*_Ni_*x*_O_4_ formation and their conversion to active Ni(OH)_2_ on the electrode surface. This behavior has also been observed in many mixed metal–nickel oxides.^21,40,41^

**Figure 9:**
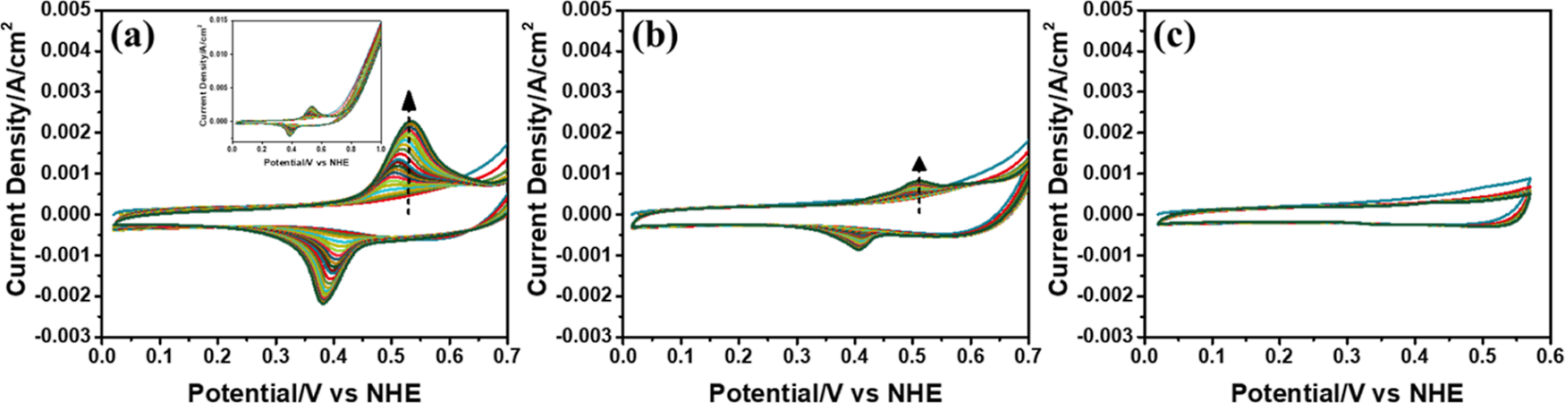
CV curves of the F-LiMn_1.33_Ni_0.67_O_4_ electrode in 1 M KOH electrolyte solution at 50 mV/s scan rate, cycled between (a) 0 and 1, (b) 0 and 0.7, and (c) 0 and 0.55 V (vs NHE).

The NiO/Ni(OH)_2_ transformation enhances if the electrodes are used at more positive potentials, at which OER occurs extensively (compare the CVs in Figure 9). An increase in the new redox peak current correlates with the H_3_O^+^ ion concentration on the electrode surface. Remember that the water oxidation reaction uses OH^–^ ions that are produced by water self-dissociation, and increases the H_3_O^+^ concentration on the electrode surface. Thus, the pH at the electrode–electrolyte interface significantly decreases. According to the Pourbaix diagram of nickel,^42^ the soluble Ni(II) species form acidic media. Sweeping the electrode in the reverse cycle to a negative potential causes the redeposition of Ni(II) ions to the electrode surface as Ni(OH)_2_. When the potential range for the CV measurement is set between 0 and 0.75 V (limited OER) and between 0 and 0.55 V (no OER), the enhancement on the redox peak currents at 0.45 V (vs NHE) is diminished and disappears, respectively, during 300 CV cycles; see Figure 9. This experiment proves that the OER enhances the Ni(OH)_2_ accumulation over the electrode surface, means that the increased H_3_O^+^ concentration on the electrode surface triggers the formation of Ni(OH)_2_ and its accumulation by cleaning the electrode surface for a fresh NiO or nickel-rich LiMn_2–*x*_Ni_*x*_O_4_ surface for the next cycle. This process is repeated in each CV cycle and results in an accumulation of more Ni(OH)_2_ via acid-assisted refurnishing of the electrode surface.

Figure S26f displays the current density variation at 1 V (vs NHE) in the CVs of the F-LiMn_2–*x*_Ni_*x*_O_4_ electrodes. Notice that the current density of all of the F-LiMn_2–*x*_Ni_*x*_O_4_ electrodes decreases in the first 100 cycles. Notice that the current density drop is more in the LiMn_1.9_Ni_0.1_O_4_ composition but becomes small when the nickel is increased over 15%. The drop in the current density may be attributed to the Mn(VI)-DR that still takes place more extensively in lower nickel contents. The electrode with a significantly high nickel content, such as the F-LiMn_1.33_Ni_0.67_O_4_ electrode, is highly stable after 200 cycles; the surface composition after 200 cycles is likely unique for a stable electrode.

The electrodes used (after 300 CVs) are further characterized by collecting their XPS spectra in the O 1s, K 2p, Mn 3s and 2p, and Ni 2p regions. Figure S27a,b shows the spectra of the O 1s and K 2p spectra, respectively. The O 1s spectra display a weak shoulder at 531.2 eV, similar to the other LiMn_2–*x*_M_*x*_O_4_ samples due to surface hydroxides with an intense peak at 529.4 eV due to lattice oxygens; see Figure S27a. K 2p XPS spectra display two peaks, likely due to intercalated K^+^ ions into the electrodes; see Figure S27b. Like the other electrodes, the potassium-ion intercalation diminishes with an increase in nickel in the LiMn_2–*x*_Ni_*x*_O_4_ structure. The splitting energy of the Mn 3s peaks is 4.4 eV for all compositions (Figure S28a). It means that the Mn(IV) species are dominant on the electrode surface. Figure S28b shows Mn 2p (^2^P_3/2_) spectra with a typical MnO_2_ peak shape and correlates with the Mn 3s region. The Ni 2p (^2^P_3/2_) region shows no difference in the spectra in terms of peak shape and satellite binding energies after the 300 CV cycling experiment; see Figure S28c, indicating the nickels are still in +2/+3 oxidation states on the electrode surface.

Figure S29 shows the plots of the percent Mn in the initial coating solutions versus the experimental surface Mn percent (evaluated from the XPS survey spectra). Notice that the composition from LiMn_2_O_4_ to LiMn_1.5_Ni_0.5_O_4_ displays almost a linear drop, similar to that of the LiMn_2–*x*_Co_*x*_O_4_ electrodes up to 25% Ni. It means that the nickel is homogeneous in the electrodes up to *x* = 0.5, but the electrode surface of F-LiMn_1.33_Ni_0.67_O_4_ is rich with Mn (Figure S29). The F-LiMn_1.33_Ni_0.67_O_4_ electrode loses some Mn through its disproportion reaction during 300 CV cycles, but it is still higher than the initial amount in the mother liquor. The CV cycling in a 1 M KOH solution generally reduces the Mn content in all compositions, as indicated by the red dots in Figure S29. The excess surface Mn undergoes a DR, resulting in a nickel-rich new surface. After each CV cycle, the new surface produces more Ni(OH)_2_ as discussed above; see Figure 9a. Therefore, the Ni(OH)_2_ accumulation, evident from increasing redox peaks at 0.45 V with cycling, is also a result of Mn(VI)-DR and overrules the formation of NiO and nickel-rich LiMn_2–*x*_Ni_*x*_O_4_ formation on the electrode surface during the calcination process. It is rather the Mn-rich surface that is the cause of Ni(OH)_2_ accumulation through Mn(VI)-DR during CV cycling. It has been well-established that at more positive potentials (OER potentials),^11,20−22,28−30^ the Mn(VI)-DR as well as the H_3_O^+^ ion formation is significantly enhanced that collectively modify the electrode surface to a more stable and efficient one.

### Electrochemical Characterization of the LiMn_2–*x*_Cu_*x*_O_4_ Electrodes

Similarly, a series of LiMn_2–*x*_Cu_*x*_O_4_ electrodes are prepared and characterized electrochemically by first recording CVs in 1 M LiNO_3_ with a 5 mV/s scan rate, Figure 6g. Figure 6h displays the plot of charge capacity under the Mn(III) oxidation peaks versus the Mn % in the original preparation solution. The decay in the charge capacity is sharper and exponential with decreasing Mn %.

The scan rate-dependent CVs of the LiMn_2–*x*_Cu_*x*_O_4_ electrodes are also similar to those of the other metal systems. This indicates that copper is also homogeneously incorporated into the LiMn_2_O_4_ spinel structure up to a certain copper percentage. The electrodes are further tested for the OER stability in 1 M KOH electrolyte by recording 300 CVs between 0 and 1 V (vs NHE); see Figure S30a–e. The CVs of the F-LiMn_2_O_4_ and F-LiMn_1.9_Cu_0.1_O_4_ electrodes are very similar, but when the copper content reaches to 15% and higher, the decay in current density becomes more significant; see Figure S30c–e. This might be attributed to phase separation of the electrode composition into CuO and LiMn_2–*x*_Cu_*x*_O_4_, as previously evaluated in the X-ray Photoelectron Spectroscopy section. The plot of current density at 1 V versus cycle number revials that the F-LiMn_2–*x*_Cu_*x*_O_4_ electrodes are not stable at OER potentials; see Figure S30f. Notice that the F-LiMn_2_O_4_ and F-LiMn_1.9_Cu_0.1_O_4_ electrodes display high current density at the first cycles. Still, the other electrodes, namely, F-LiMn_1.7_Cu_0.3_O_4_, F-LiMn_1.5_Cu_0.5_O_4_, and F-LiMn_1.33_Cu_0.67_O_4_, display current densities consistently below 10 mA/cm^2^ at 1 V and decay with increasing CV cycles. This behavior is also related to the lower electrocatalytic activity of CuO-based materials in the OER because the Cu(II) species cannot be oxidized to a higher oxidation state, which is necessary for an efficient OER. Formation of H_3_O^+^ during the OER on the electrode surface not only dissociates the Mn(VI)-DR product but also dissolves the CuO from the electrode surface. Since the CV cycling is carried out in 1 M KOH, the dissolution of CuO and formation of Cu(OH)_2_ are in competition to convert the CuO sides to Cu(OH)_2_ during CV measurement
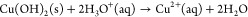
4

XPS spectra of the F-LiMn_2–*x*_Cu_*x*_O_4_ electrodes used are recorded to further characterize the electrode surface upon CV cycling. Figures S31a,b display the O 1s and K 2p spectra of all of the F-LiMn_2–*x*_Cu_*x*_O_4_ electrodes, respectively. The O 1s spectrum displays a strong hydroxyl feature at 531.2 eV that is attributed to surface Cu(OH)_2_ species; see Figure S31a. If the origin of K 2p peaks is a potassium-ion intercalation into the structure, it is favored in the LiMn_1.7_Cu_0.3_O_4_ composition but significantly diminished in the LiMn_1.33_Cu_0.67_O_4_ composition; see Figure S31b. Moreover, the splitting energy of the Mn 3s peaks slightly increases with increasing copper; see Figure S31c; it is also attributed to a phase separation of the CuO species. The Mn 2p (^2^P_3/2_) region resembles the MnO_2_ peak shape in all three compositions (Figures S31d and S14) and correlates with the Mn 3s spectra. The Cu(II) species are dominant in the Cu 2p spectra even after the CV cycling experiment on the surface of both F-LiMn_1.7_Cu_0.3_O_4_ and F-LiMn_1.33_Cu_0.67_O_4_ electrodes (Figure S31e), also showing the stability of the Cu(OH)_2_. Furthermore, the XPS survey spectra of the F-LiMn_2–*x*_Cu_*x*_O_4_ electrodes used show an accumulation of Mn on the electrode surface with increasing copper composition (Figure S31f). Moreover, the Mn amount further increases in the LiMn_1.33_Cu_0.67_O_4_ electrode after 300 CVs (Figure S31f). Increased Mn must be related to a faster dissociation of Cu(OH)_2_ during OER, likely due to a reaction with H_3_O^+^ ions produced from OER on the electrode–electrolyte interface; see eq 4.

### OER Performance of the F-LiMn_2–*x*_M_*x*_O_4_ Electrodes

Mass activities of the electrodes are calculated to compare the catalytic activities of the electrodes with each other and are reported in Table 2. The weights of the electrodes are determined from the charge capacity, evaluated from the 1 M LiNO_3_ CV cycling experiments; see eq 5. However, calculation of the F-LiMn_2–*x*_M_*x*_O_4_ electrode weight (EW) is more complex than that for the F-LiMn_2_O_4_ electrode. Notice that one Mn is +3 and the other is +4 in the F-LiMn_2_O_4_ electrode. Thus, the charge capacity in the CV curve is due to oxidation of Mn(III) to Mn(IV) and directly correlates with the amount of LiMn_2_O_4_. However, with the addition of another metal, the Mn(III) content decreases because the other metals are in either +3 or +2 oxidation states (because of their high electronegativity, the Mulliken electronegativities of Mn, Fe, Co, Ni, and Cu are 3.71, 4.06, 4.30, 4.40, and 4.48 eV, respectively). Therefore, the compositions are rather LiMn^4+^Mn^3+^_1–*x*_M^3+^_*x*_O_4_ or LiMn^4+^_1+*x*_Mn^3+^_1–2*x*_M^2+^_*x*_O_4_ in the M(III) or M(II) electrodes, respectively. The evaluated charge-capacity results are from the oxidation of (1 – *x*) and (1 – 2*x*) Mn(III) to Mn(IV) for the electrodes with M(III) and M(II), respectively. Using this assumption, we evaluated a correction factor (CF) for each electrode and used it to calculate the calculated EW (CEW) from the measured Mn-based EW (Mn-EW). The Mn-EWs and CEWs are evaluated using eq 5, where *q* is the specific charge capacity of the electrode (in A·s/cm^2^), *M*_wt_ is the molecular weight of LiMn_2–*x*_M_*x*_O_4_ (in g/mol), CF is the CF (unitless), *e* is electron charge (1.6 × 10^–19^ A·s/e), *N* is Avogadro’s number (6.022 × 10^23^ e/mol), and *A* is 0.837 and a constant (the % lithium deintercalation, previously evaluated from the XRD data)^11^
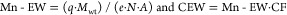
5

**Table 2:** Table of CEW and Mass Activity (MA) of the LiMn _2–*x*_M_*x*_O_4_ Electrodes at η = 350 mV

electrode	Mn-EW (μg/cm^2^)	CEW (μg/cm^2^)	MA (A/g)	electrode	Mn-EW (μg/cm^2^)	CEW (μg/cm^2^)	MA (A/g)
LiMn_2_O_4_	35.5	35.5	50.2	LiMn_1.9_Ni_0.1_O_4_	20.1	25.1	72.4
LiMn_1.9_Fe_0.1_O_4_	30.3	33.7	58.8	LiMn_1.7_Ni_0.3_O_4_	6.4	16	129.6
LiMn_1.7_Fe_0.3_O_4_	23.1	33.1	56.8	LiMn_1.5_Ni_0.5_O_4_	3.5		
LiMn_1.5_Fe_0.5_O_4_	17.5	35.0	41.1	LiMn_1.33_Ni_0.67_O_4_	3.1		
LiMn_1.33_Fe_0.67_O_4_				LiMn_1.9_Cu_0.1_O_4_	29.1	36.4	56.7
LiMn_1.9_Co_0.1_O_4_	26.7	29.6	96.2	LiMn_1.7_Cu_0.3_O_4_	16.8	36.5^a^	39.2
LiMn_1.7_Co_0.3_O_4_	17.1	27.5	141.4	LiMn_1.5_Cu_0.5_O_4_	8.8	36.5^a^	34.5
LiMn_1.5_Co_0.5_O_4_	12.9	25.9	154.4	LiMn_1.33_Cu_0.67_O_4_	4.3	38.7^a^	
LiMn_1.33_Co_0.67_O_4_	8 0.2	24.6	180.4				

a Without CuO.

The CF is given by 1/(1 – *x*) if M is in a +3 oxidation state in the lattice and calculated to be 1, 1.11, 1.43, 2, and 3 for the LiMn_2_O_4_, LiMn_1.9_M_0.1_O_4_, LiMn_1.7_M_0.3_O_4_, LiMn_1.5_M_0.5_O_4_, and LiMn_1.33_M_0.67_O_4_ compositions, respectively. However, if M is in a +2 oxidation state, the CF is given by 1/(1 – 2*x*), and it is 1, 1.25, and 2.5 for the LiMn_2_O_4_, LiMn_1.9_M_0.1_O_4_, and LiMn_1.7_M_0.3_O_4_ compositions, respectively, and no Mn(III) sites are left in the structure for *x* is 0.5 and above. Likely, CF is 1/(1 – *x*) for the iron and cobalt, between 1/(1 – *x*) and 1/(1 – 2*x*) for the nickel, and 1/(1 – 2*x*) for the copper (assuming all Cu is in +2 oxidation state) compounds. The CEWs are comparable to each other and are around 35 μg/cm^2^ for the F-LiMn_2–*x*_Fe_*x*_O_4_ electrodes (no effect of Fe(III) salt in the coating thickness) and almost the same as the F-LiMn_2_O_4_ electrode. Note also that 35 μg is impossible to measure using a high precision 4-digit laboratory balance. The CEW of the LiMn_2–*x*_Co_*x*_O_4_ electrodes is slightly less and changes from 30 to 25 μg/cm^2^ with increasing *x* from 0.1 to 0.67, respectively. It means that the electrodes become thinner with increasing Co(II) in the initial coating solution. If we assume cobalt is in a +3 oxidation state, the CF values are 1.11, 1.43, 2.00, and 3.00 in the LiMn_2–*x*_Co_*x*_O_4_, where x is 0.1, 0.3, 0.5, and 0.67, respectively, and consistent with the data tabulated in Table 2. However, the nickel electrodes behaved somewhat differently. The F-LiMn_2–*x*_Ni_*x*_O_4_ electrodes are even thinner, and the thickness decreases at a higher rate when the Ni(II) in the coating solution increases. This may be related to the amount of water that an initially coated LLC phase holds; it is known that the [Ni(H_2_O)_6_](NO_3_)_2_-surfactant LLC mesophase holds relatively more water.^43,44^ As a result, the initial LLC-LiMn_2–*x*_Ni_*x*_-P123 films are thinner than the LLC-LiMn_2–*x*_Co_*x*_-P123 films (see later). Moreover, the nickel in the F-LiMn_2–*x*_Ni_*x*_O_4_ electrodes is in a +2 oxidation state at low nickel concentrations, but some become Ni^3+^ with an increasing *x* in the structure. It is difficult to predict the Ni^3+^/Ni^2+^ ratio since both the thickness of the electrodes and the Ni^2+^/Ni^3+^ ratio decrease with increasing *x*. However, we still observe the Mn^3+^/Mn^4+^ redox peaks even in the F-LiMn_1.5_Ni_0.5_O_4_ and F-LiMn_1.33_Ni_0.67_O_4_ electrodes due to the Mn-rich surface. Therefore, the nickel electrodes above *x* of 0.5 form nickel-rich LiMn_2–*x*_Ni_*x*_O_4_ (where *x* > 0.5) on the FTO surface with a Mn-rich surface layer (LiMn_2–*x*_Ni_*x*_O_4_, where *x* < 0.5) on top of the electrodes. Note also that the above observations are also consistent with the literature results of the LiMn_1.5_Ni_0.5_O_4_ electrodes that display two redox peaks due to Ni^2+^/Ni^3+^ and Ni^3+^/Ni^4+^ couples in their CVs in addition to Mn^3+^/Mn^4+^ redox peaks.^45−47^ Accordingly, the CEW values of the F-LiMn_2–*x*_Ni_*x*_O_4_ electrodes are calculated using eq 5 and tabulated in Table 2 by assuming all the nickels are in +2 oxidation state in the F-LiMn_1.9_Ni_0.1_O_4_ and F-LiMn_1.7_Ni_0.3_O_4_ electrodes. Still, in the other compositions, it is difficult to determine the CEWs because both electrode thickness change and phase separation occur at the same time above 0.5 Ni samples.

However, in the LiMn_2–*x*_Cu_*x*_O_4_ samples, there is a clear phase separation, and the CuO phase forms at high Cu contents.^48^ If we assume all copper is in a +2 oxidation state, the CF will be 1/(1 – 2*x*). The CEW can be calculated assuming a homogeneous mixing of Cu^2+^ in the LiMn_2–*x*_Cu_*x*_O_4_ electrode up to almost 0.3 (about 0.27 in the spinel and 0.03 in the CuO phase). Both F-LiMn_2–*x*_Cu_*x*_O_4_ electrodes (where *x* is 0.1 with a CF value of 1.25 and 0.3 with a CF value of 2.17) have similar CEWs (36.375 and 36.525 μg/cm^2^, respectively). Since more than half of the active EW is still observed from the F-LiMn_1.5_Cu_0.5_O_4_ electrode, some of the Cu^2+^ must separate to form the CuO phase. Comparing the CEW values of the F-LiMn_1.73_Cu_0.27_O_4_ and F-LiMn_1.5_Cu_0.5_O_4_ electrodes, the F-LiMn_1.5_Cu_0.5_O_4_ electrode has a CEW of 36.52 μg/cm^2^ and CF of 4.15 (corresponding to a composition of LiMn_1.62_Cu_0.38_O_4_ and the remaining 0.12 Cu^2+^ is in the CuO phase). Similarly, F-LiMn_1.33_Cu_0.67_O_4_ has a CEW of 38.7 μg/cm^2^ with a CF of 8.53 and a composition of LiMn_1.56_Cu_0.44_O_4_ (with a 0.23 CuO phase). Therefore, the phase separation is almost zero in the LiMn_1.9_Cu_0.1_O_4_ composition, but gradually increases to 10, 24, and 34% of the Cu^2+^ with increasing *x* to 0.3, 0.5, and 0.67, respectively, in the F-LiMn_2–*x*_Cu_*x*_O_4_ electrodes. Accordingly, the actual EW is more than that reported here, but the CuO phase is not active in the OER. Note also that the CuO phase becomes visible in the XRD pattern of the LiMn_1.33_Cu_0.67_O_4_ sample.

The variation in the CEWs of the F-LiMn_2–*x*_M_*x*_O_4_ electrodes has an essential consequence in terms of LLC-LiMn_2–*x*_M_*x*_-P123 gel-film thickness that depends on the water content of the mesophase due to the second metal ion in the mixture. Some metal ions [such as Co(II) and Ni(II)] hold more water in the gel phase and produce thinner films. For example, the nitrate salts of Co(II), Ni(II), Mn(II), and Cu(II) have 6, 6, 4, and 3 water per metal ion, respectively, and it is even less in the gel phase. Note also that the FTIR spectra of salt-surfactant mesophases of these metal salts display the most intense water peaks in the case of Ni(II), compared to the other metal ions,^43,44^ and it is the least in the Cu(II) case. The nitrate asymmetric stretching modes, observed at around 1290–1500 cm^–1^, are also susceptible to the water amount and show that more water is in the nickel samples.^43,44^ Therefore, the water content of all of the LLC-LiMn_2–*x*_M_*x*_-P123 samples needs to be accurately determined to fully elucidate the origin of the variation in the electrode thickness, but this is kept outside the scope of this work to instead focus on the main topic of this work.

The current density of the same electrodes at 0.75 V (vs NHE or 1.575 V vs RHE, and corresponds to an overpotential of 350 mV) is extracted from the second CVs (recorded between 0 and 1 V in 1 M KOH electrolyte solution). Then, each current density is divided by the CEWs and tabulated in Table 2 as the MA. It is also important to note that the MAs are not measured quantities from a gram of F-LiMn_2–*x*_M_*x*_O_4_ electrodes. They are evaluated and tabulated to compare the electrodes with each other and similar electrodes in the literature. The estimated MAs of the LiMn_2–*x*_Fe_*x*_O_4_ electrodes are approximately 50 A/g at 350 mV overpotential. Thus, adding iron into the F-LiMn_2–*x*_Fe_*x*_O_4_ electrodes has almost no effect on the OER electrocatalysis. It is also important to note that these electrodes are not stable under an alkaline solution during the OER. However, adding only 5% Co into the F-LiMn_2_O_4_ electrode improves the MA to 81.1 A/g. Increasing cobalt content further increases the mass activities. The highest MA is found to be 126.8 A/g for the F-LiMn_1.33_Co_0.67_O_4_ electrode. The nickel electrodes (LiMn_2–*x*_Ni_*x*_O_4_) display a similar trend. The MA increases with increasing x in the LiMn_2–*x*_Ni_*x*_O_4_ electrodes, and it is 72.4 A/g in the F-LiMn_1.9_Ni_0.1_O_4_ electrode and increases to 129.6 A/g in the F-LiMn_1.7_Ni_0.3_O_4_ electrode. The F-LiMn_2–*x*_Cu_*x*_O_4_ electrodes show smaller MA values but are close to the F-LiMn_2_O_4_ and F-LiMn_2–*x*_Fe_*x*_O_4_ electrodes at a low *x* but further decrease with increasing Cu(II) in the electrodes as expected because copper is “impossible” to further oxidize to carry an efficient OER.

Since the F-LiMn_1.33_M_0.67_O_4_ electrodes are mechanistically more critical (see later), they are further tested at higher current densities (10 to 50 mA/cm^2^) to check their stabilities and shine some light on the OER mechanism. Previously, it has been shown that the Mn(VI)-DR is driven by the presence of 3Mn(VI) species side by side, where the middle Mn(VI) side is more electronegative (due to its number of oxygen neighbors; see later) and gets reduced to Mn(IV) by oxidizing the other two Mn(VI) sides to two [MnO_4_]^−^ ions. The [MnO_4_]^−^ ions dissociate into an electrolyte solution and cause electrode degradation.^11^ Releasing the permanganate ion is enhanced by the local H_3_O^+^ ions produced from the water oxidation at the electrode surface.

The F-LiMn_1.33_M_0.67_O_4_ electrodes are further investigated for their OER performance and stability in alkaline media. Notice that this specific composition (*x* = 0.67) is suggested as a unique one to prevent Mn(VI)-DR that requires 3Mn(VI) sides (lattice–O–Mn(==O)_2_–O–Mn(=O)_2_–O–Mn(=O)_2_–O–lattice, where Mn=O sides are active sides) connected on the electrode surface as the origin of the F-LiMn_2_O_4_ electrode degradation.^11^ Among the three Mn(VI), the middle Mn(VI) has two Mn=O sides (two oxo bonds) and two bridging oxygens (−O–Mn(=O)_2_–O−) bonded to the terminal Mn(VI) sides and is more electronegative compared to the terminal ones (lattice–O–Mn(=O)_2_–O–). This connectivity can be broken by the replacement of at least one of the terminal Mn(VI) sides with another highly electronegative metal ion, such as Co, Ni, and Cu. Iron can be introduced as Fe^3+^, but its effect is limited because of two reasons; (i) small electronegativity difference between Mn and Fe and (ii) Fe(V) that forms during OER also undergoes DR^39^ and degrades during OER.

Among these metals, unfortunately, the Cu^2+^ ion cannot be further oxidized to a higher oxidation state for effective water oxidation and incorporated into the LiMn_1–*x*_Cu_*x*_O_4_ lattice in +2 oxidation state; therefore, the performance of the copper incorporated electrodes is not as good as the electrodes with the other two metals (Co and Ni). Moreover, the nickel also undergoes a phase separation at high Ni/Mn mole ratios due to nickels being incorporated in a +2 oxidation state (as depicted from Ni 2p XPS spectrum). Incorporation of the M^2+^ ion is limited due to charge balance in the LiMn_2–*x*_M_*x*_O_4_ (maximum *x* can be 0.5, if M is in +2 oxidation state) lattice. Therefore, in the LiMn_1.33_Ni_0.67_O_4_, some nickels also undergo phase-separation as NiO and affect the electrochemical properties of the electrodes (see the Electrochemical Characterization of the LiMn_2–*x*_Cu_*x*_O_4_ Electrodes section). Homogeneous incorporation of 0.67 M^3+^ ion into the LiMn_1.33_M_0.67_O_4_ lattice brakes above Mn(VI)–Mn(VI)–Mn(VI) connectivity (forms during the OER on the electrode surface) to Mn(VI)–M(X)Mn(VI) (or Mn(VI)–Mn(VI)–M(X), where X is IV or V), hinders the Mn(VI)-DR, and stabilizes the electrode surface. Therefore, cobalt is the only metal that can be incorporated up to 0.67 as Co^3+^ in the LiMn_1.33_Co_0.67_O_4_ lattice and produce stable electrodes. However, even though nickel incorporation is limited to 0.5, the nickel-containing electrodes are still stable (because of Ni(OH)_2_ formation over the electrode surface) and highly active in the OER.

**Figure 10:**
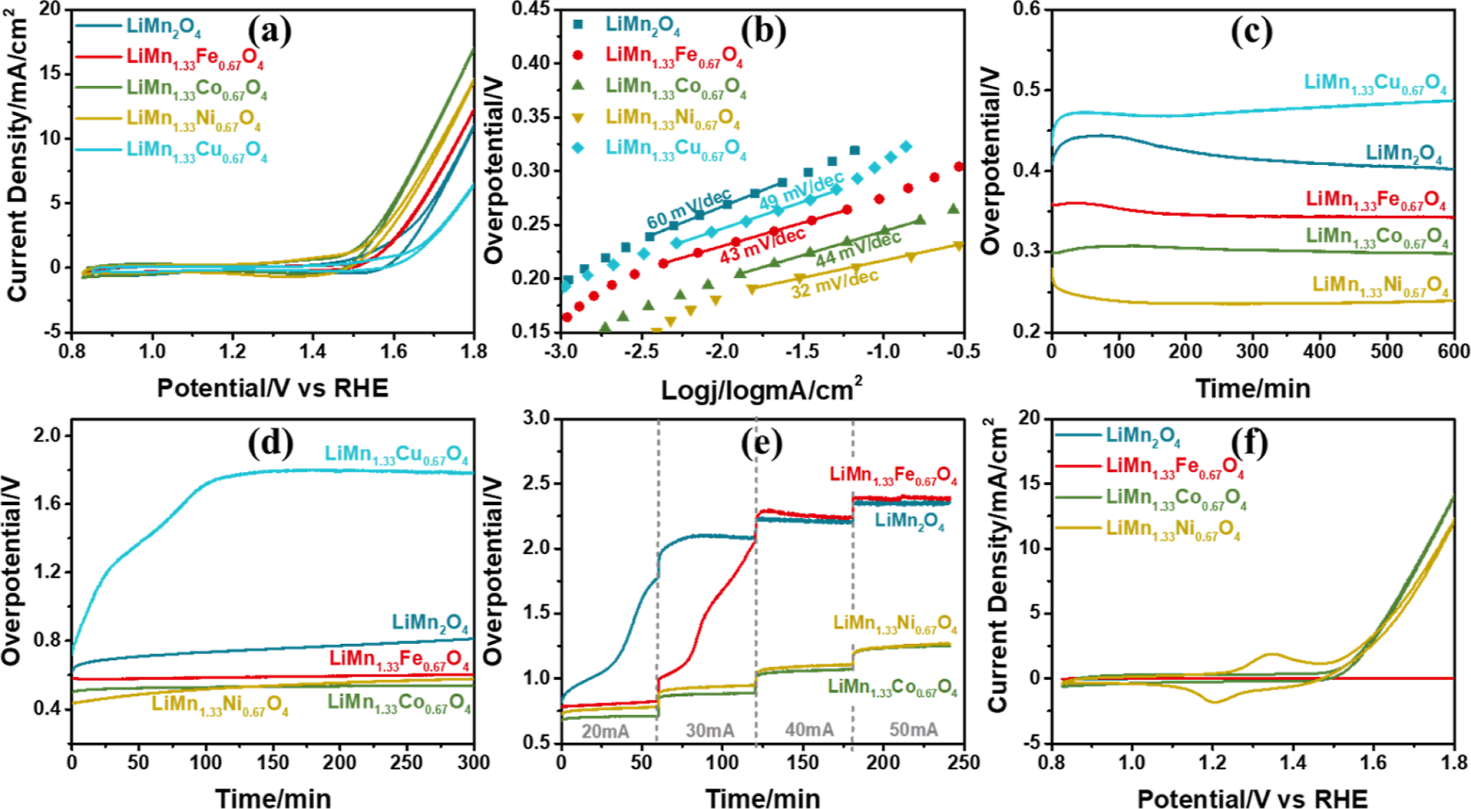
(a) CV curves of the F-LiMn_1.33_M_0.66_O_4_ electrodes in 1 M KOH electrolyte solution with a 50 mV/s scan rate and (b) Tafel slope analysis of the F-LiMn_1.33_M_0.67_O_4_ electrodes. CP curves of the F-LiMn_1.33_M_0.67_O_4_ electrodes at (c) 1 and (d) 10 mA/cm^2^ current densities. (e) m-CP results of the F-LiMn_1.33_M_0.67_O_4_ electrodes and (f) CV curves of the F-L-iMn_1.33_M_0.67_O_4_ electrodes after CP experiments.

For a detailed OER analysis, the F-LiMn_1.33_M_0.67_O_4_ electrodes have been used in 1 M KOH solution for 300 CVs, followed by m-CA and multistep CP (m-CP, from 1 to 50 mA/cm^2^) measurements. Finally, another set of CVs is recorded to check the electrode stability. The second CVs and CAs of the F-LiMn_1.33_M_0.67_O_4_ electrodes are shown in Figure 10a,b, respectively. The F-LiMn_2_O_4_ electrode has the highest Tafel slope (60 mV/dec) that decreases to 49 mV/dec in the F-LiMn_1.33_Cu_0.67_O_4_ electrode and further decreases with the incorporation of iron, cobalt, and nickel to 43, 44, and 32 mV/dec, respectively. Also, note that the F-LiMn_1.33_Cu_0.67_O_4_ electrode undergoes phase separation and produces a CuO phase. Therefore, it may not be meaningful to compare with the other electrodes. The presence of a more electronegative metal improves the OER kinetics of the Mn sites through synergistic electronic interactions of Mn with the neighboring metal ions.^21^ The lowest Tafel slope is observed from the F-LiMn_2–*x*_Ni_*x*_O_4_ electrodes because nickel is the most electronegative ion among these metals (Mn, Fe, Co, and Ni) that can be homogeneously incorporated into the spinel structure. Also, the Ni(OH)_2_ formation/deposition on the electrode surface may improve the OER performance of the nickel-based electrodes.^28^

The overpotential increases during CP measurements in the first 50 min and may be attributed to potassium-ion deintercalation from the F-LiMn_2_O_4_ electrode surface. Then, a slight decrease in overpotential is observed, likely due to cleaning the surface of Mn species through Mn(VI)-DR and making the electrode surface more active for the OER. The CP curve of the F-LiMn_1.33_Cu_0.67_O_4_ electrode at 1 mA/cm^2^ current density shows the highest overpotential value and quickly riches to a bare FTO value at 10 mA/cm^2^ current density (Figure 10c,d) and is attributed to electrode decomposition. Therefore, the F-LiMn_1.33_Cu_0.67_O_4_ electrode has not been further investigated.

The other F-LiMn_1.33_M_0.67_O_4_ electrodes are also used in long-term CP experiments at 1 mA/cm^2^ current density (Figure 10c). The overpotential decreases by incorporating 33% Fe, Co, or Ni to 352, 297, or 239 mV, respectively. Again, the nickel sample has the lowest overpotential in addition to the lowest Tafel slope. These improvements have already been attributed to the second metal ions’ synergistic electronic effect (electronegativity). The spinel LiMn_2–*x*_Ni_*x*_O_4_ structure keeps nickel at low nickel concentrations (*x* = 0.5 and lower). Only the F-LiMn_1.33_Ni_0.67_O_4_ electrode produces Ni(OH)_2_ species on the electrode surface with CV cycling.

The F-LiMn_1.33_M_0.67_O_4_ electrodes are further used in the CP experiments at 10 mA/cm^2^ current density; see Figure 10d. One would expect an overpotential increase of as much as a Tafel slope at 10 mA/cm^2^ to 390, 341, and 271 mV for the F-LiMn_1.33_Fe_0.67_O_4_, F-LiMn_1.33_Co_0.67_O_4_, and F-LiMn_1.33_Ni_0.67_O_4_ electrodes, respectively, but the observed overpotentials are much higher and 604, 540, and 578 mV, respectively; the higher overpotentials are due to high electrode resistance that needs to be reduced by choosing more conducting substrates (see later). The same electrodes are further used for the CP experiments at 20 to 50 mA/cm^2^ current densities (Figure 10e) to check their stability at higher current densities.

The F-LiMn_2_O_4_ electrode at 20 mA/cm^2^ current density undergoes a Mn(VI)-DR. In 60 min, the overpotential reaches the bare FTO value, which is attributed to electrode decomposition. The F-LiMn_1.33_Fe_0.67_O_4_ electrode resists DR up to 30 mA/cm^2^ current density; see Figure 10e. As previously discussed, iron also undergoes degradation at high potentials and forms soluble [FeO_4_]^2–^ species. The F-LiMn_1.33_Co_0.67_O_4_ and F-LiMn_1.33_Ni_0.67_O_4_ electrodes can be used under harsh conditions; they are stable within 20 to 50 mA/cm^2^ current density. It is expected that the F-LiMn_1.33_Ni_0.67_O_4_ electrode would perform better in the OER than the F-LiMn_1.33_Co_0.67_O_4_ electrode. However, at higher current densities, the electrode resistance becomes more important and affects the overpotential values. Therefore, it is necessary to use substrates with low resistance to compare the overpotentials of the electrodes at high current densities.

Finally, the CVs of the used electrodes are recorded once more between 0 and 1 V (vs NHE) to check the electrode stability after long CP experiments; see Figure 10f. Notice that F-LiMn_2_O_4_ and F-LiMn_1.33_Fe_0.67_O_4_ electrodes do not show any faradaic peaks in the voltammograms, showing a typical bare FTO response. The F-LiMn_1.33_Co_0.67_O_4_ electrode has almost the same voltammogram as the first CV of the unused electrode, indicating: (i) homogeneous incorporation of cobalt into the spinel structure, (ii) stabilizing the electrode, (iii) coherently working with Mn in the OER catalysis, and (iv) synergistically improving the OER performance of Mn sites on the electrode surface.

**Figure 11:**
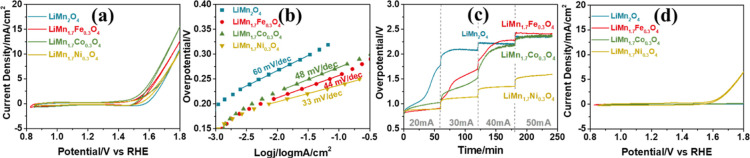
(a) CV curves of the F-LiMn_1.7_M_0.3_O_4_ electrodes (M is Mn, Fe, Co, and Ni) in 1 M KOH electrolyte solution with a 50 mV/s scan rate, (b) Tafel slope analysis of the F-LiMn_1.7_M_0.3_O_4_ electrodes, (c) m-CP plots of the F-LiMn_1.7_M_0.3_O_4_ electrodes, (d) CV curves of the same electrodes after CP experiments.

In the F-LiMn_1.33_Ni_0.67_O_4_ case, even though the electrode is stable and efficient, a complete incorporation of nickel into the spinel structure is still under debate because the redox peaks due to Ni(OH)_2_/NiOOH couple are observed; see Figure 10f. Moreover, NiOOH formation on the electrode surface may also improve the water oxidation performance. Due to Ni(OH)_2_ formation on the electrode surface at high nickel concentrations, another composition, namely, F-LiMn_1.7_M_0.3_O_4_, was also investigated to compare the OER performance of the F-LiMn_1.7_M_0.3_O_4_ electrodes (M is Fe, Co, Ni, and Cu) that preserve the spinel structure without any phase separation in electrodes. Therefore, the F-LiMn_1.7_M_0.3_O_4_ electrodes are also tested by CV cycling (0 to 1 V), CA, and CP (at 1, 10, 20, 30, 40, and 50 mA/cm^2^ current densities) measurements in 1 M KOH electrolyte solution; see Figure 11a. All electrodes have no redox peak(s) in the 0 to 0.7 V potential window. Both F-LiMn_1.7_Fe_0.3_O_4_ and F-LiMn_1.7_Co_0.3_O_4_ electrodes provide a high current density and can be attributed to improved electrocatalytic OER performance. However, initially, the F-LiMn_1.7_Ni_0.3_O_4_ electrode works very similarly to the F-LiMn_2_O_4_ electrode. This is attributed to the less reactive Mn oxide species accumulating on the electrode surface. Thus, the F-LiMn_1.7_Ni_0.3_O_4_ electrode needs to be activated by further CV cycling or CP experiments.

Then, the F-LiMn_1.7_M_0.3_O_4_ electrodes are also used for CA experiments to extract their Tafel slopes. Figure 11b shows the Tafel plots of the F-LiMn_2_O_4_, F-LiMn_1.7_Fe_0.3_O_4_, F-LiMn_1.7_Co_0.3_O_4_, and F-LiMn_1.7_Ni_0.3_O_4_ electrodes. The Tafel slopes are 60, 44, 50, and 33 mV/dec for the same electrodes, respectively. These values are similar to those of F-LiMn_1.33_M_0.67_O_4_ electrodes, except the F-LiMn_1.33_Co_0.67_O_4_ electrode, which has a slightly higher Tafel slope. Then, the same electrodes are further used for long-term CP experiments at 1 and 10 mA/cm^2^ current densities; see Figure S32a,b, respectively. Overpotentials of the F-LiMn_1.7_M_0.3_O_4_ electrodes are lower than those of the F-LiMn_2_O_4_ electrode but slightly higher than those of the F-LiMn_1.33_M_0.67_O_4_ electrodes. Nyquist plots of the F-LiMn_1.7_M_0.3_O_4_ electrodes are shown in Figure S33. Different potential biases are applied in the range of the OER. Especially at the bias of 1.5 V (vs RHE), a distinct difference of F-LiMn_2_O_4_ can be seen, which resulted in a repressed semicircle with a much larger charge transfer resistance (*R*_ct_). This can be easily correlated to the Tafel curves provided in Figure 11b. Further applied bias resulted in almost similar Nyquist curves, which might be due to the mesoporous nature of the electrodes. Nevertheless, all of the electrodes have roughly an *R*_ct_ value in the range of 5–15 Ω, indicating their exceptional kinetics due to the mesoporosity. At the bias of 1.6 V (vs RHE), the F-LiMn_1.7_Ni_0.3_O_4_ electrode has the highest *R*_ct_ value, despite having the smallest Tafel slope and high stability. We believe that the reason for this is the formation of bubbles due to the OER, which is especially higher in the F-LiMn_1.7_Ni_0.3_O_4_ electrode due to the aforementioned properties. All in all, although it is possible to discern some differences at the onset of the OER reaction, it is not possible to make a clear deduction in terms of performance beyond 1.6 V vs RHE.

The F-LiMn_1.7_M_0.3_O_4_ electrodes are also tested at high current densities to investigate their degradation behaviors, see Figure 11c. The m-CPs of the F-LiMn_1.7_Fe_0.3_O_4_ and F-LiMn_1.7_Co_0.3_O_4_ electrodes are similar, and their degradation occurs between 20 and 50 mA/cm^2^ current densities. This is expected for the iron case since it is also unstable, even at much higher compositions. However, decreasing cobalt content in the electrode (from 33 to 15%) makes the electrode unstable; the F-LiMn_1.7_Co_0.3_O_4_ electrode undergoes a gradual degradation starting from 20 mA/cm^2^ current density. The F-LiMn_1.7_Ni_0.3_O_4_ electrode remains stable during the CP experiments at all current densities; see Figure 11c. The CVs (after complete CP tests) also correlate with the above observations. Only the F-LiMn_1.7_Ni_0.3_O_4_ electrode is stable and active in the OER region without showing any Ni(OH)_2_ formation on the electrode surface; see Figure 11d.

In summary, the F-LiMn_1.33_Co_0.67_O_4_ and F-LiMn_1.7_Ni_0.3_O_4_ electrodes have good OER performance and are highly stable. The F-LiMn_1.33_Ni_0.67_O_4_ electrode also outperforms by altering the surface composition (making a Ni(OH)_2_-rich surface). At higher current densities, the overpotentials remain constant in long CP experiments. Therefore, they can be considered robust electrodes under harsh electrocatalytic OER conditions. Overpotentials are higher for these thin film electrodes at high current densities because of a high resistance of the FTO substrate. A typical resistance in a F-LiM_2–*x*_M_*x*_O_4_ electrode varies from 11 to 18 Ω that causes an IR drop of 110 to 180 mV at a 10 mA/cm^2^ and 550 to 900 mV at 50 mA/cm^2^ current density, respectively. Compensating the IR drop will bring the overpotentials to a reasonable value. A more conductive substrate, such as graphite or stable metal sponge substrates, may be used to overcome resistance issues and high overpotentials.

**Figure 12:**
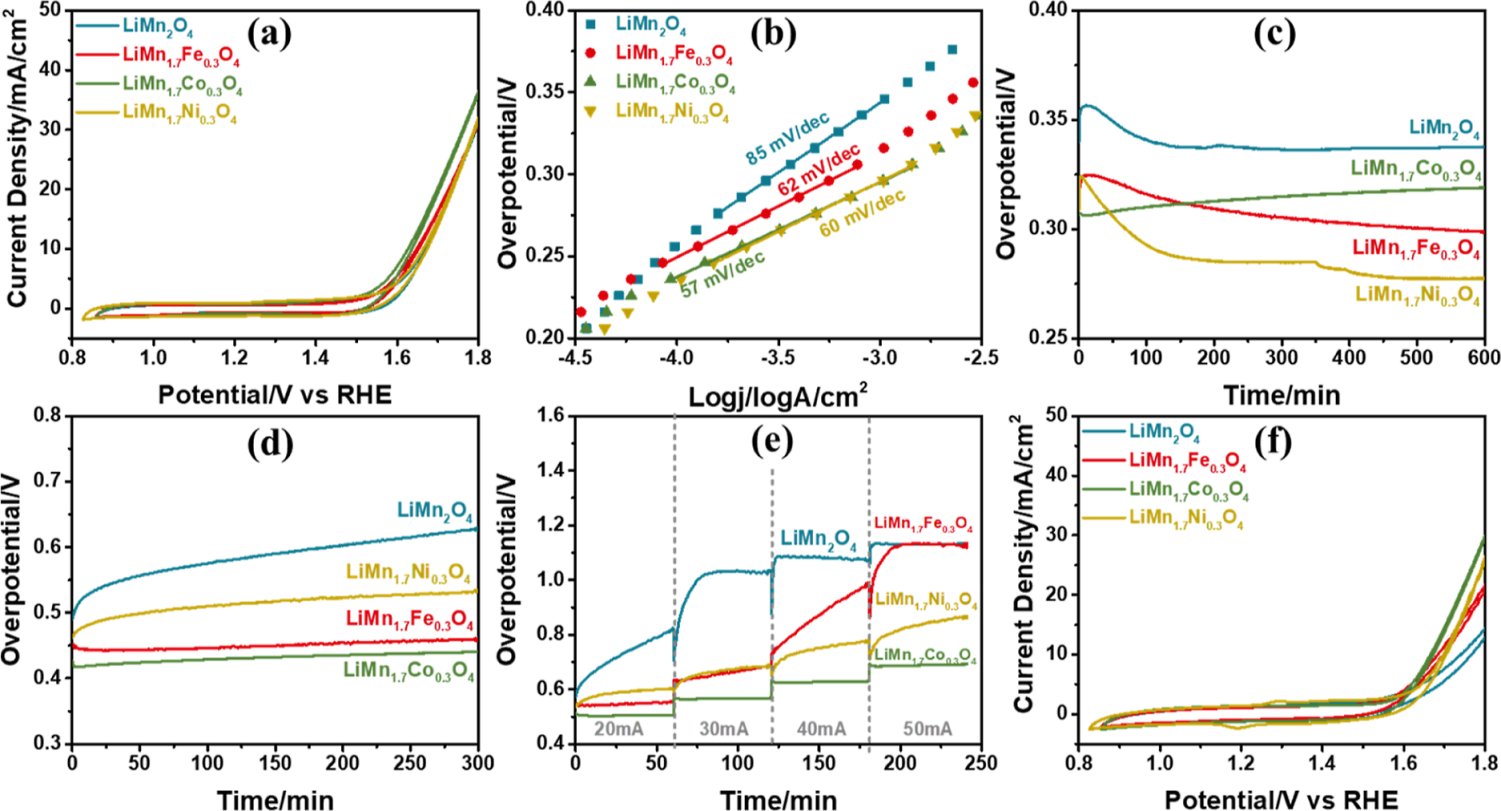
G-LiMn_1.7_M_0.3_O_4_ (M = Fe, Co, and Ni) electrodes: (a) CV curves, (b) CA curves [overpotential vs log(*j*)], (c) CP curves at 1 mA/cm^2^ current density electrodes, (d) CP curves at 10 mA/cm^2^ current density electrodes, (e) m-CP plots, and (f) CV curves of the same electrodes after CP experiments.

To further elucidate the above issues, another set of electrodes is also fabricated using 5-times diluted solutions of the LiMn_1.7_M_0.3_O_4_ and LiMn_2_O_4_ compositions by dip coating on graphite rods (1 cm^2^), followed by calcination at 300 °C to obtain the G-LiMn_1.7_M_0.3_O_4_ (G stands for graphite rod) electrodes. Note that 5× dilution ensures film thickness similar to spin-coated films without dilution. These electrodes are also used in 1 M KOH to collect their CVs, CAs, and CPs (Figure 12). The CVs show a much higher current density at 1 V (vs NHE) with a steeper rise in the OER potentials than the FTO-coated electrodes, typical for a better conducting electrode. Tafel slopes are evaluated from the CA data and found out that the G-LiMn_2_O_4_ electrode has a higher Tafel slope (85 mV/dec) than the FTO-LiMn_2_O_4_ electrode; the other electrodes have a Tafel slope of ∼60 mV/dec (specifically, 62, 57, and 60 mV/dec for the G-LiMn_1.7_M_0.3_O_4_ electrodes, where M is Fe, Co, and Ni, respectively), but slightly higher compared to the F-LiMn_1.7_M_0.3_O_4_ electrodes. Even though the Tafel slopes are higher for the G-LiMn_1.7_M_0.3_O_4_ electrodes, their overpotential values are much lower at high current densities, as shown in the CPs in Figure 12e. The CP measurements show that the G-LiMn_2_O_4_ and G-LiMn_1.7_Fe_0.3_O_4_ electrodes also degrade quickly at high current densities, but the G-LiMn_1.7_Co_0.3_O_4_ electrode is the most robust and displays relatively low overpotentials at high current densities. A more conductive graphite substrate (around 2–5 Ω) positively affects the overpotentials; the substrate’s resistance is the primary factor in the overpotentials at high current densities and is more critical than Tafel slopes. It is also important to note that for an industrial level application, much higher current densities are required during OER;^49−51^ therefore, the series resistant of the electrochemical cell becomes more important. Table S3 compares our results with similar compounds in the recent literature.^52−59^ While our Tafel slopes are similar or lower, the overpotential values at 10 mA/cm^2^ are comparable. However, it is only reasonable to compare the overpotentials if these measurements use identical amounts of material.

## Conclusions

Mesoporous LiMn_2–*x*_M_*x*_O_4_ (where M is Fe, Co, Ni, and Cu and *x* is 0, 0.1, 0.3, 0.5, and 0.67) thin films and electrodes (on FTOs, F-LiMn_2–*x*_M_*x*_O_4_ and graphite rods, G-LiMn_2–*x*_M_*x*_O_4_) can be fabricated by employing the MASA method that uses clear ethanol solutions of the ingredient metal salts and surfactants. The coated solution immediately loses its volatile solvent (ethanol) to form a LLC mesophase. The water content of the mesophase, which is highly dependent on the second metal ion, determines the film thickness. Calcination of the mesophase gives mesoporous thin films/electrodes. The *meso*-LiMn_2–*x*_Fe_*x*_O_4_ film surface is partly covered with FeCO_3_ nanoparticles. However, Co is homogeneously distributed in the spinel LiMn_2–*x*_Co_*x*_O_4_ lattice. Similarly, Ni is homogeneous in LiMn_2–*x*_Ni_*x*_O_4_ at low Ni %, but its surface becomes Mn-rich over 25%. Copper also forms LiMn_2–*x*_Cu_*x*_O_4_ films/electrodes at low Cu %, but the CuO phase forms at higher copper percentages.

These electrodes display two redox peaks at 0.9 and 1.1 V (vs NHE) due to lithium–ion deintercalation via oxidation of Mn(III) sides to Mn(IV) and intercalation via reduction of the same sides in the reverse process in two steps in all spinel LiMn_2–*x*_M_*x*_O_4_ electrodes. The redox peaks gradually lose their peak currents with increasing M in the spinel structure and almost vanish in the F-LiMn_1.33_M_0.67_O_4_ electrodes, indicating that the Mn(III) sites are replaced with the M(III)/M(II) ions in the spinel structure. The redox peaks of the M(III) sides must be at more positive potentials. Introducing a small amount of M (in all metals, Fe, Co, Ni, and Cu) improves the OER performance and enhances the stability of the electrodes. Still, the F-LiMn_2–*x*_Fe_*x*_O_4_ electrodes undergo degradation at all compositions through Mn(VI)- and Fe(V)-DR or oxidation of iron sides to Fe(VI) as soluble [FeO_4_]^2–^ species. The other electrodes also degrade at low M concentrations, but the F-LiMn_1.33_Co_0.67_O_4_ and F-LiMn_1.7_Ni_0.3_O_4_ electrodes are highly stable even at much higher current densities during the OER. The F-LiMn_1.33_Ni_0.67_O_4_ electrode is also stable and displays excellent performance in the OER, but with the usage at more positive potentials, Ni(OH)_2_ formation is observed on the electrode surface. The synergistic electronic effect plays a vital role in the stability of the LiMn_2–*x*_M_*x*_O_4_ electrodes if M is homogeneously distributed in the structure. The Mn(VI)-DR requires [lattice–O–Mn(VI)(=O)_2_–O–Mn(VI)(=O)_2_–O–Mn(VI)(=O)_2_–O–lattice] sides on the electrode surface for an effective oxidation/reduction reaction to produce 2Mn(VII) (as soluble [MnO_4_]^−^ ions) and Mn(IV) (as insoluble MnO_2_). The Mn(VI) in the middle of the above surface structure is more electronegative than the terminal ones. Therefore, there is an electron transfer from each terminal Mn(VI) side to the middle Mn(VI) side to reduce it to Mn(IV). The terminal ones get oxidized to Mn(VII) and released from the electrode surface with the help of H_3_O^+^ ions, which are produced during OER. Enhanced permanganate ion release (at higher potentials, where OER occurs more extensively) supports the above proposal. The presence of a more electronegative metal (namely, Co and Ni) prevents the Mn(VI)-DR. It stabilizes the active surface species because Co and Ni cannot be oxidized to +5 or higher oxidation states, which may be needed for the self-oxidation of the Mn(VI) sides (Mn(VI)-DR). Moreover, the thickness of the electrodes can be easily controlled by spin rate or dilution of the initial coating solutions but has almost no effect on the performance of the OER performance. These electrodes also display low Tafel slopes (fast OER) and low overpotentials at high current densities when they are fabricated over a less resistive substrate. Therefore, it is reasonable to conclude that it is not the overpotential and Tafel slopes that need to be worried about for OER electrocatalysis; it is instead the electrode resistance to be reduced for practical applications in efficient OER. Because the overpotential due to OER kinetic (by increasing the current density from 100 to 1000 mA/cm^2^ is only as much as a Tafel slope, typically 60 mV) is negligible compared to an IR drop at high current densities (by increasing 100 to 1000 mA/cm^2^, IR drop is IR, typically 2700 mV for a 3 Ω carbon rod). One needs to overcome for practical applications. Research motivation should focus more on the stability of the electrode materials at high current densities and less resistive substrates for commercial application.

## Supporting Information Available

Schematic representations, XRD patterns, XPS spectra, SEM images, CVs, CP curves, Nyquist plots, and tables of compositions and mol ratios (PDF)
