# Supplementary material for: Electronic Synergistic Effects on the Stability and Oxygen Evolution Reaction Efficiency of the Mesoporous LiMn2–xMxO4 (M = Mn, Fe, Co, Ni, and Cu) Electrodes
Source: Inorg Chem. 2024 Nov 7;63(46):22239–57. doi: 10.1021/acs.inorgchem.4c03885 (PMC11577315; doi:10.1021/acs.inorgchem.4c03885)
Supplement: Supplementary file 1 — ic4c03885_si_001.pdf [file ic4c03885_si_001.pdf]

# Supporting Information (SI)

## Electronic Synergistic Effects on the Stability and Oxygen Evolution Reaction Efficiency of the Mesoporous $LiMn_{2-x}M_xO_4$ (M = Mn, Fe, Co, Ni, and Cu) Electrodes

*Irmak Karakaya Durukan*[1] *and Ömer Dag**[1,2]

[1]Department of Chemistry, Bilkent University, 06800, Ankara, Turkey.

[2]UNAM — National Nanotechnology Research Center and Institute of Materials Science and Nanotechnology, Bilkent University, 06800, Ankara, Turkey.

**Corresponding author email:** dag@fen.bilkent.edu.tr

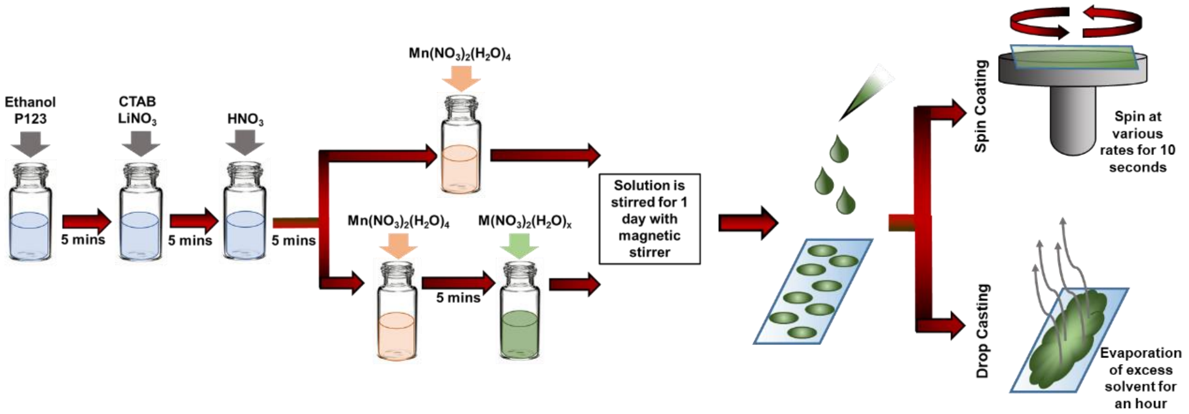


**Scheme S1.** Schematic representation of the preparation of the LLC mesophases by spin coating and drop-cast coating methods.

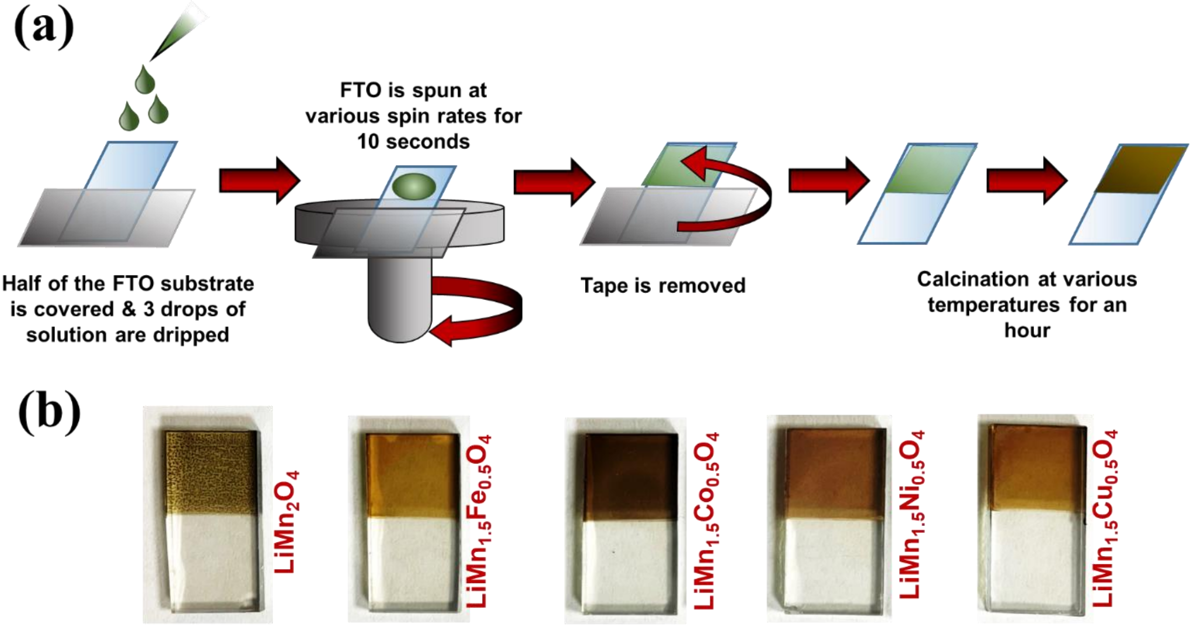


**Scheme S2.** (a) Schematic representation of electrode fabrication and (b) photographs of the electrodes (numbers are percent Mn and M in $LiMn_{2-x}M_xO_4$).

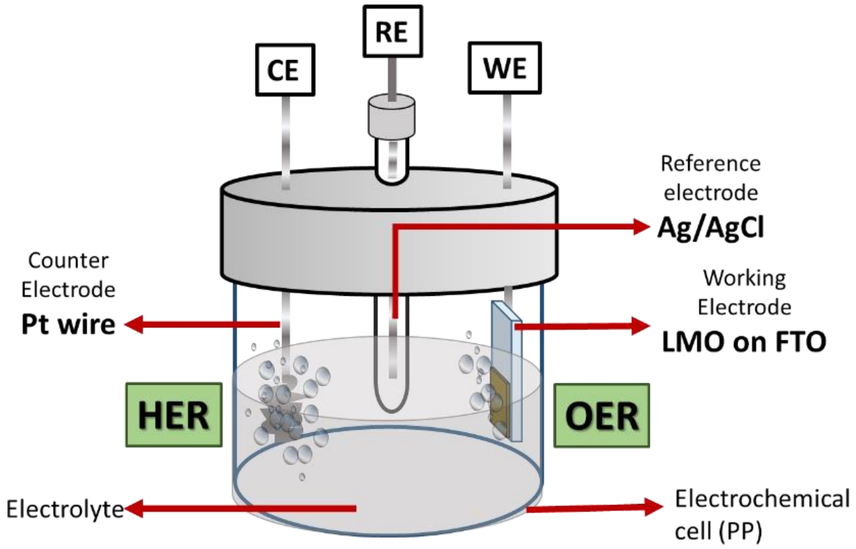


**Scheme S3.** Schematic illustration of electrochemical cell and components.

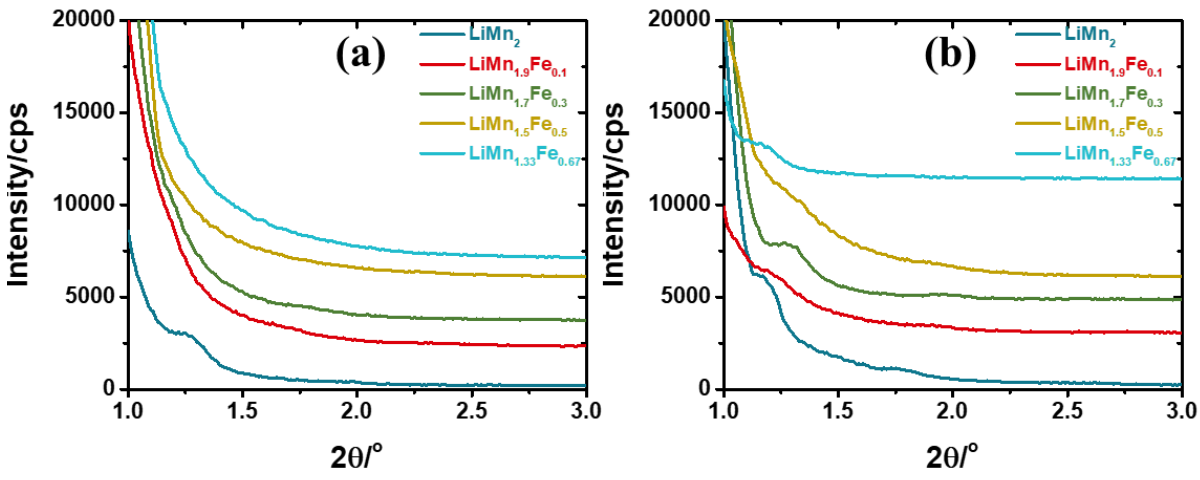


**Figure S1.** Small-angle XRD patterns of (a) fresh thin gel-mesophases and (b) 1 hour aged thick gel-mesophases of the LLC-$LiMn_{2-x}Fe_x$ samples.

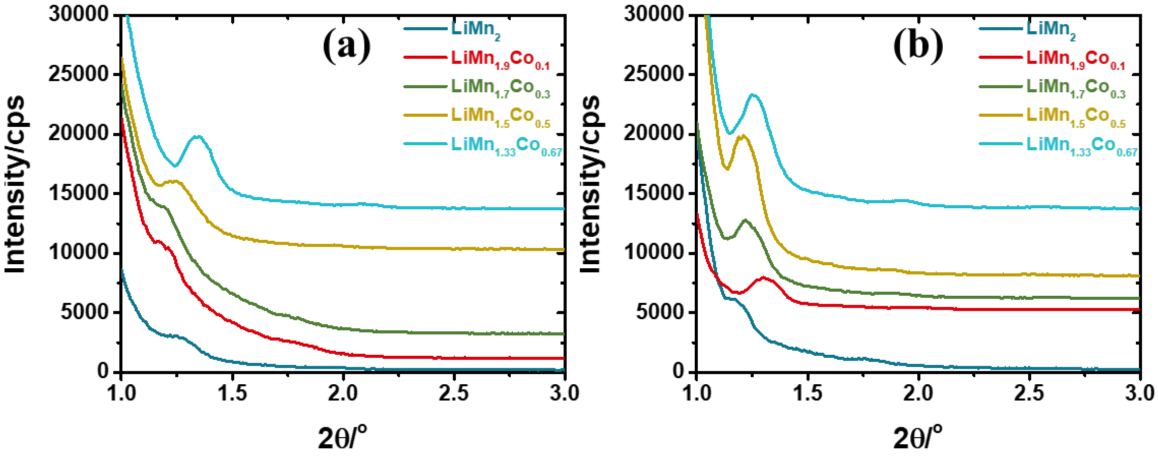


**Figure S2.** Small-angle XRD patterns of (a) fresh thin gel-mesophases and (b) 1 hour aged thick gel-mesophases of the LLC-$LiMn_{2-x}Co_x$ samples.

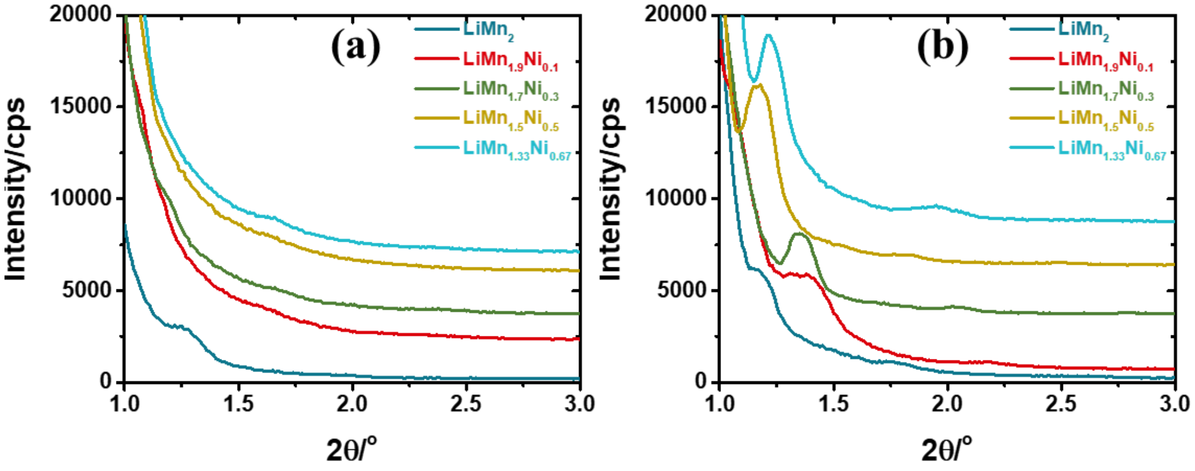


**Figure S3.** Small-angle XRD patterns of (a) fresh thin gel-mesophases and (b) 1 hour aged thick gel-mesophases of the LLC-$LiMn_{2-x}Ni_x$ samples.

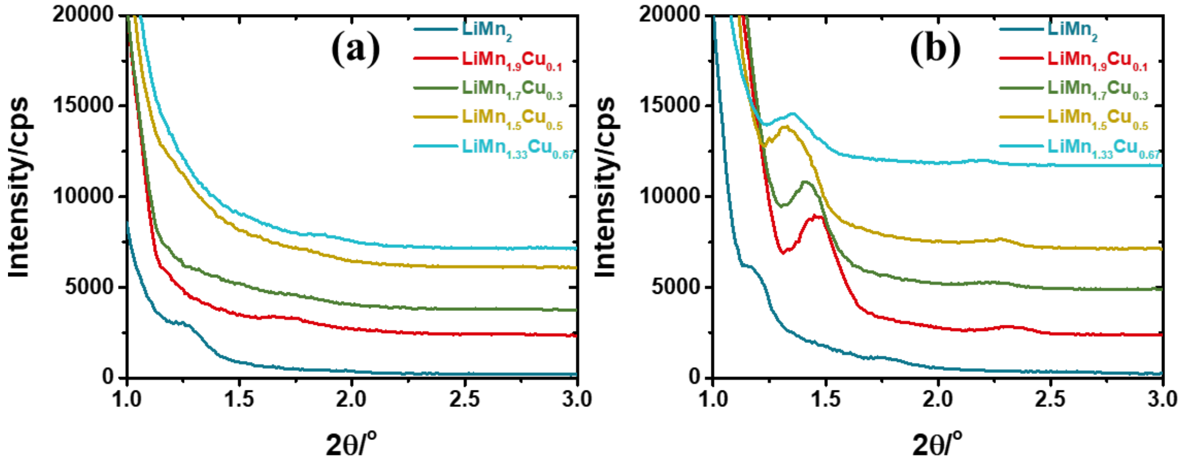


**Figure S4.** Small-angle XRD patterns of (a) fresh thin gel-mesophases and (b) 1 hour aged thick gel-mesophases of the LLC-$LiMn_{2-x}Cu_x$ samples.

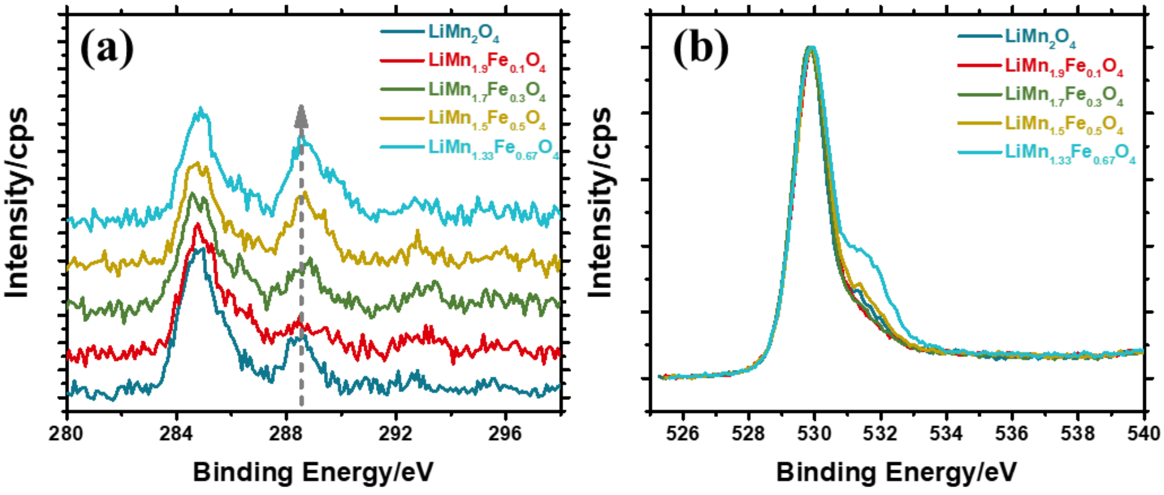


**Figure S5.** XPS spectra of *meso*-$LiMn_{2-x}Fe_xO_4$ films in the (a) C 1s, (b) O 1s regions.

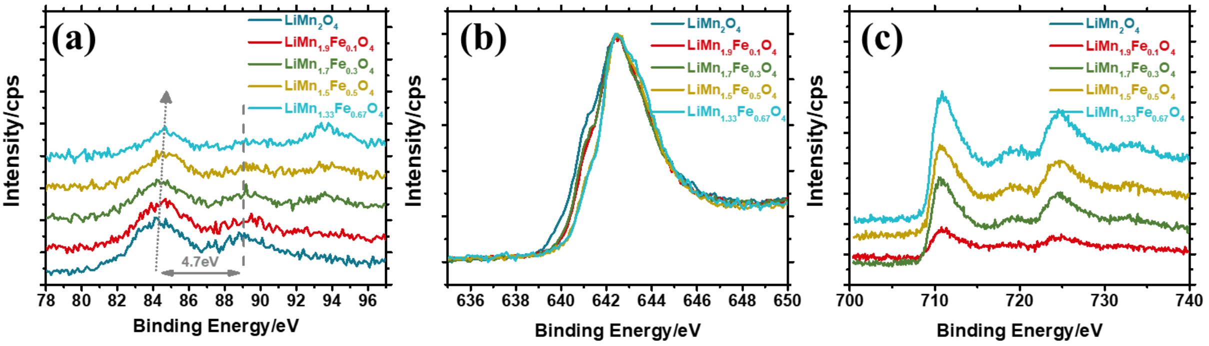


**Figure S6.** XPS spectra of *meso*-$LiMn_{2-x}Fe_xO_4$ films in the (a) Mn 3s, (b) Mn 2p ($^2P_{3/2}$), and (c) Fe 2p regions.

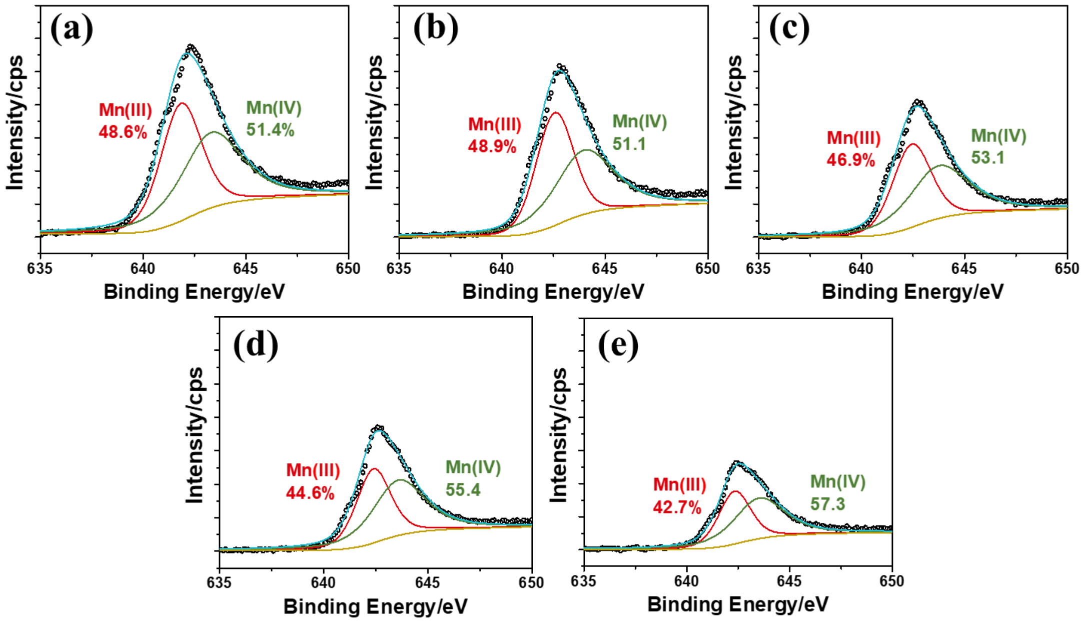


**Figure S7.** Mn 2p ($^2P_{3/2}$) XPS spectra of the $LiMn_{2-x}Fe_xO_4$ electrodes, where x is (a) 0, (b) 0.1, (c) 0.3, (d) 0.5, and (e) 0.67.

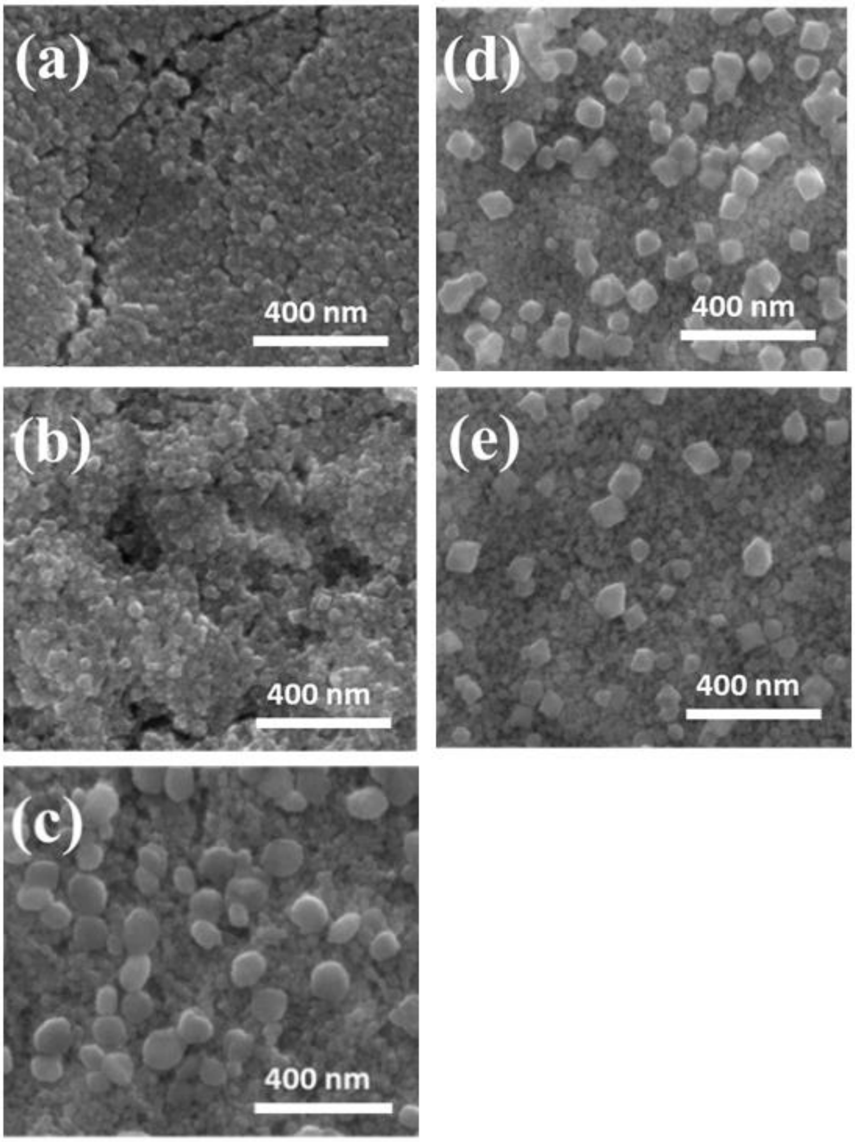


**Figure S8.** SEM images: top views of the FTO coated $LiMn_{2-x}Fe_xO_4$ samples, where x is (a) 0, (b) 0.10, (c) 0.30, (d) 0.50, and (e) 0.67.

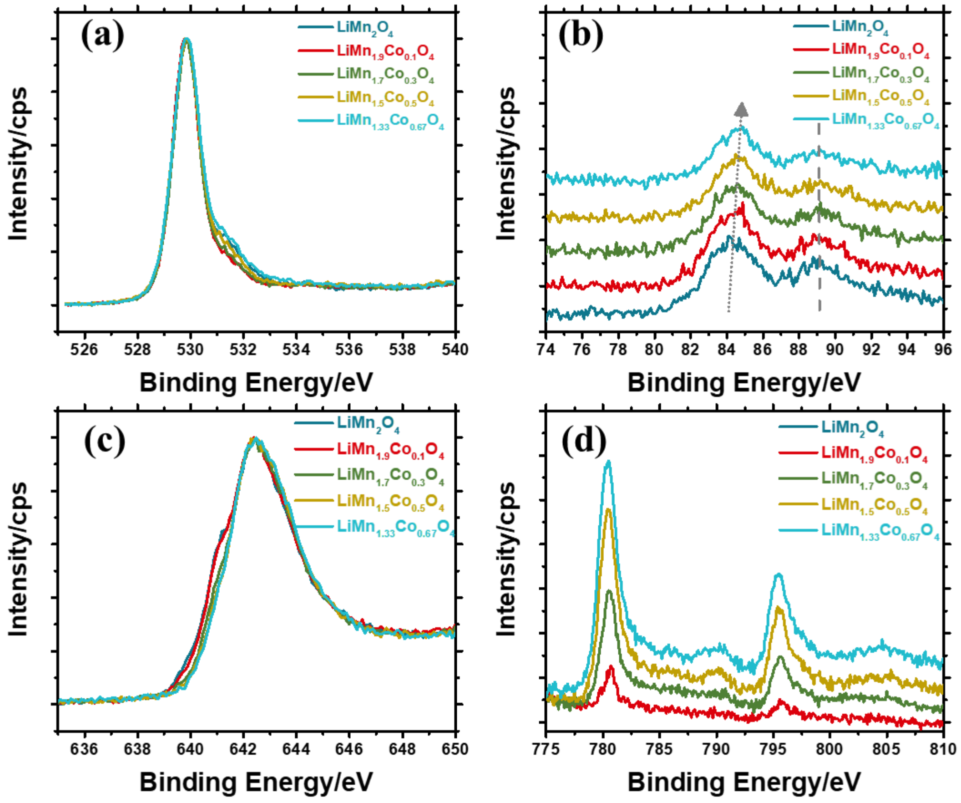


**Figure S9.** XPS spectra of *meso*-$LiMn_{2-x}Co_xO_4$ films in the (a) O 1s, (b) Mn 3s, (c) Mn 2p ($^2P_{3/2}$), and (d) Co 2p regions.

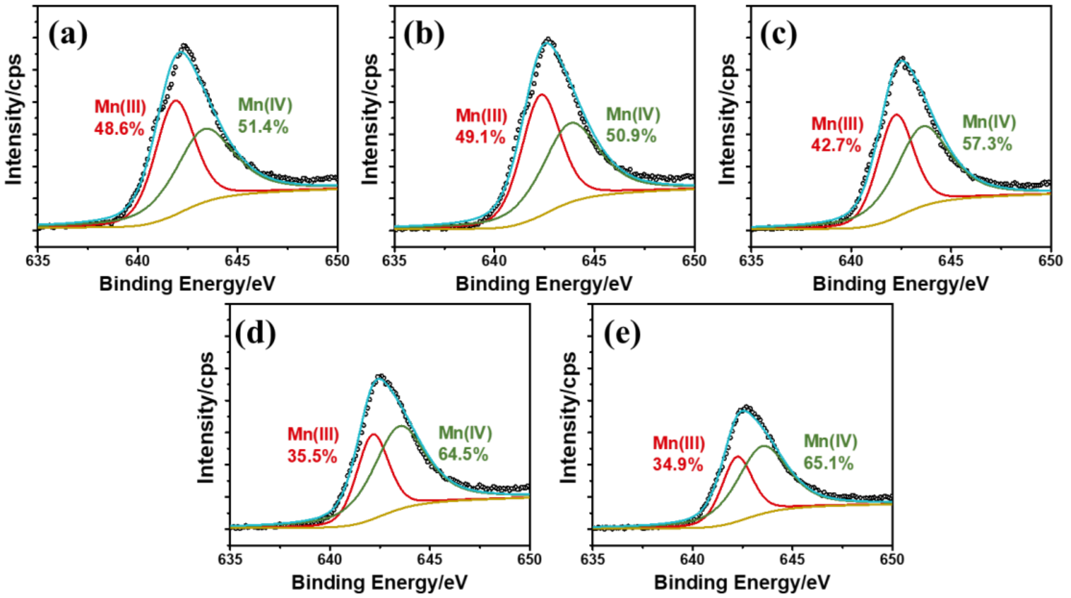


**Figure S10.** Mn 2p ($^2P_{3/2}$) XPS spectra of the $LiMn_{2-x}Co_xO_4$ electrodes, where x is (a) 0, (b) 0.1, (c) 0.3, (d) 0.5, and (e) 0.67.

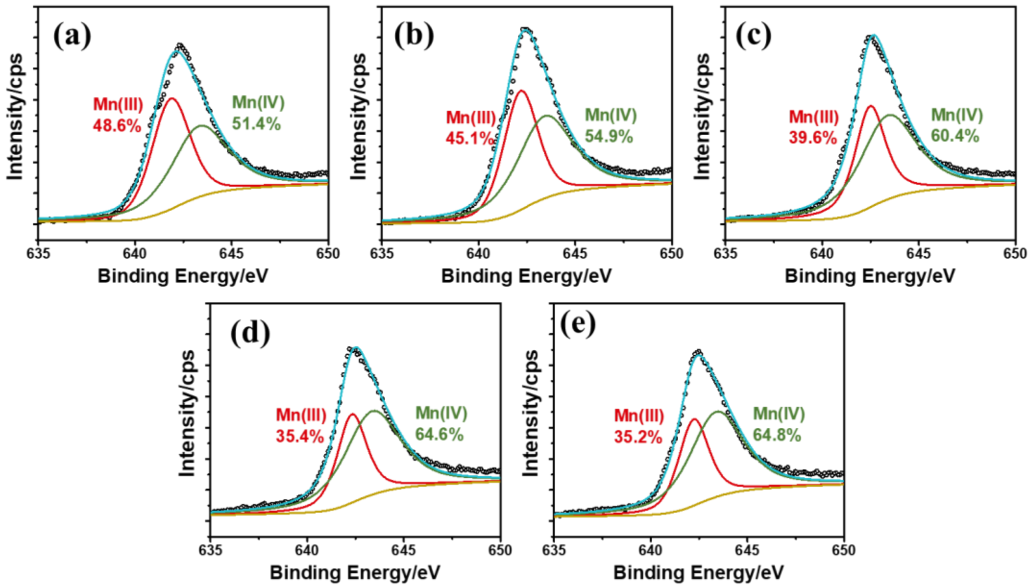


**Figure S11.** Mn 2p ($^2P_{3/2}$) XPS spectra of the $LiMn_{2-x}Ni_xO_4$ electrodes, where x is (a) 0, (b) 0.1, (c) 0.3, (d) 0.5, and (e) 0.67.

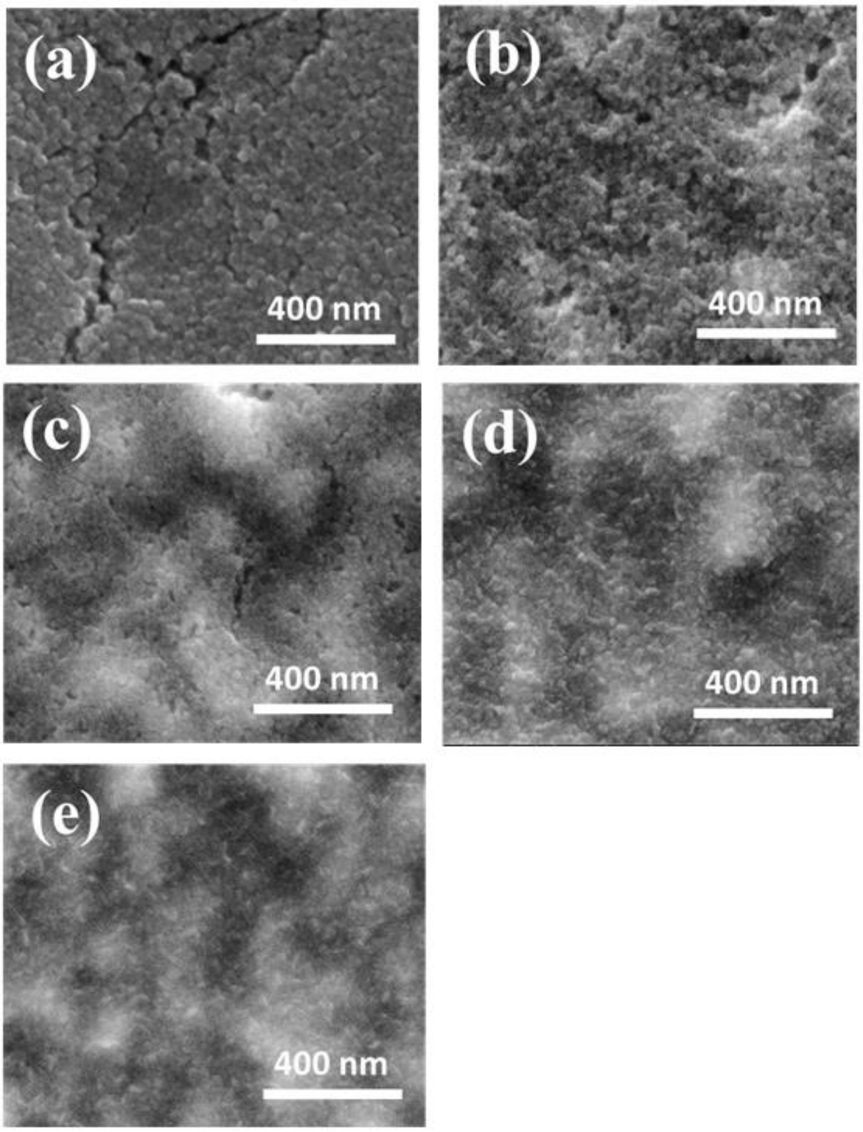


**Figure S12.** Top-view SEM images of the F-$LiMn_{2-x}Ni_xO_4$ films, where x is (a) 0, (b) 0.10, (c) 0.30, (d) 0.50, and (e) 0.67.

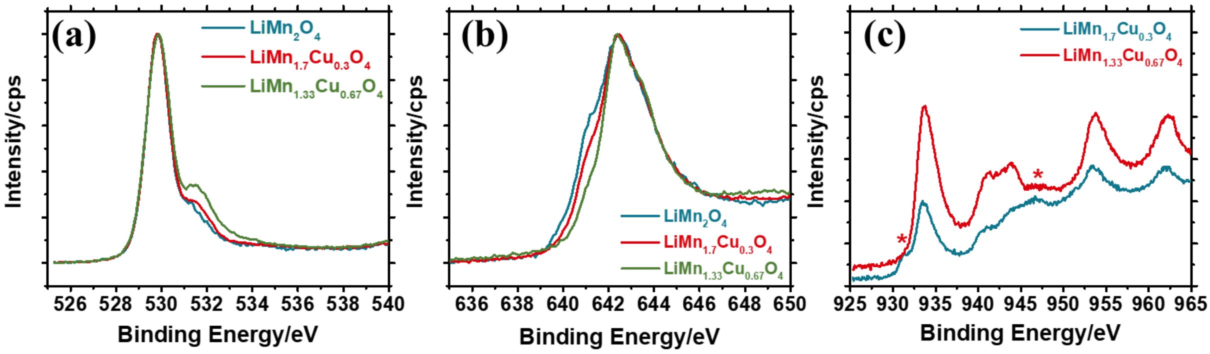


**Figure S13.** XPS spectra of the F-$LiMn_{2-x}Cu_xO_4$ films in the (a) O 1s, (b) Mn 2p ($^2P_{3/2}$), and (c) Cu 2p regions.

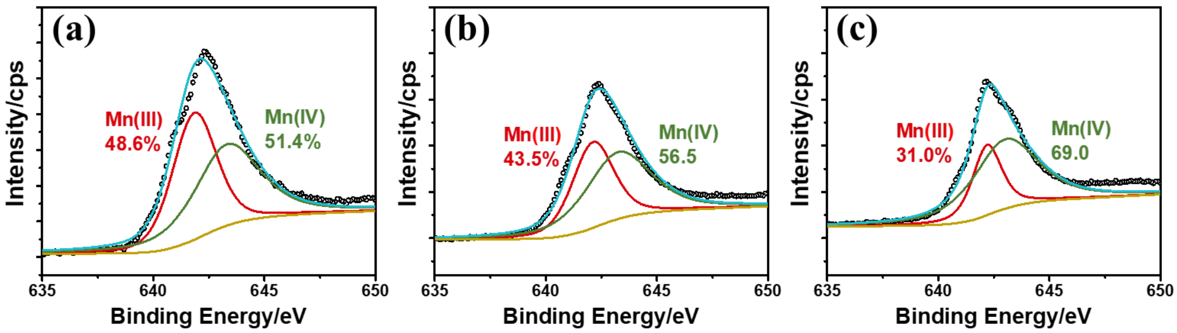


**Figure S14.** Mn 2p ($^2P_{3/2}$) XPS spectra of the $LiMn_{2-x}Cu_xO_4$ electrodes where, x is (a) 0, (b) 0.3, and (c) 0.67.

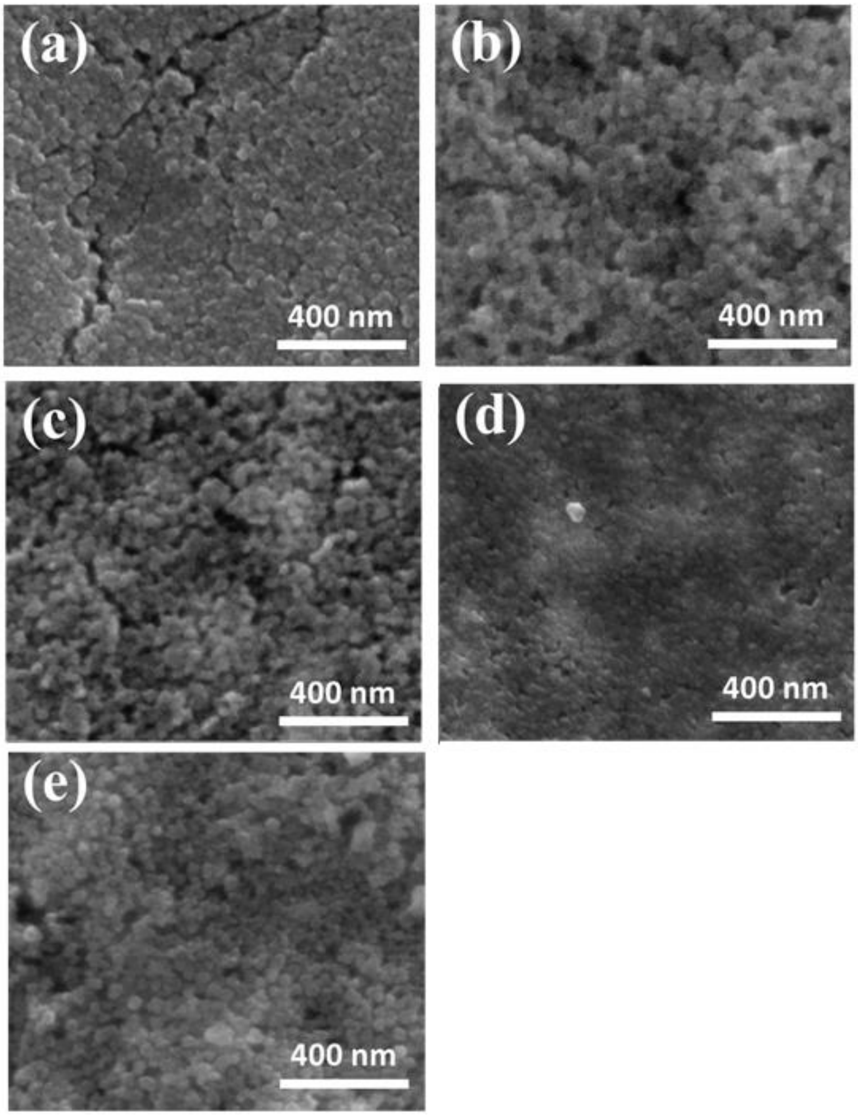


**Figure S15.** Top-view SEM images of the F-$LiMn_{2-x}Cu_xO_4$ films, where x is (a) 0, (b) 0.10, (c) 0.30, (d) 0.50, and (e) 0.67.

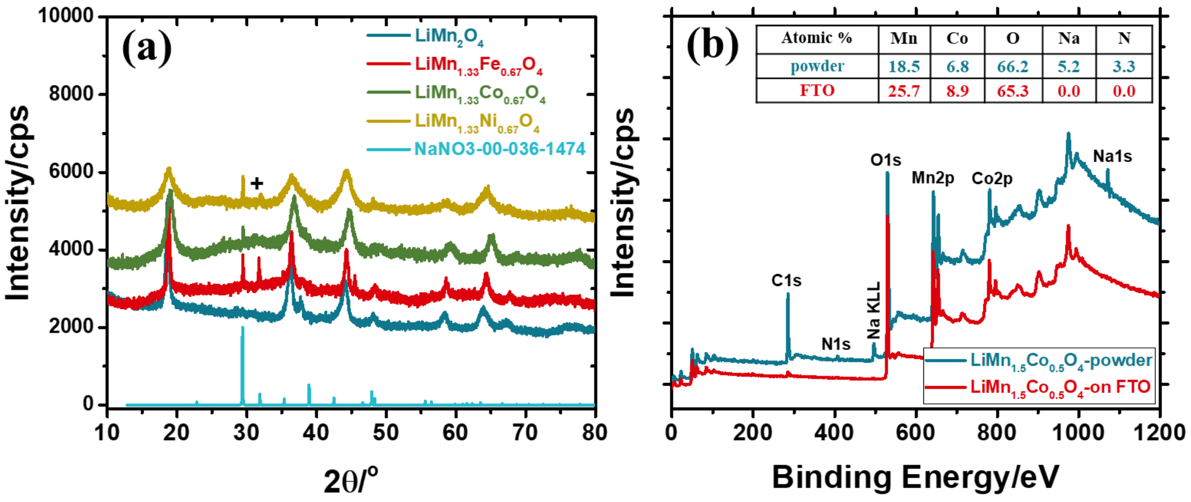


**Figure S16.** (a) XRD patterns of the $LiMn_{1.33}Fe_{0.67}O_4$, $LiMn_{1.33}Co_{0.67}O_4$, and $LiMn_{1.33}Ni_{0.67}O_4$ powders. (b) XPS survey spectra of the $LiMn_{1.5}Co_{0.5}O_4$ samples fabricated on glass and FTO surfaces.

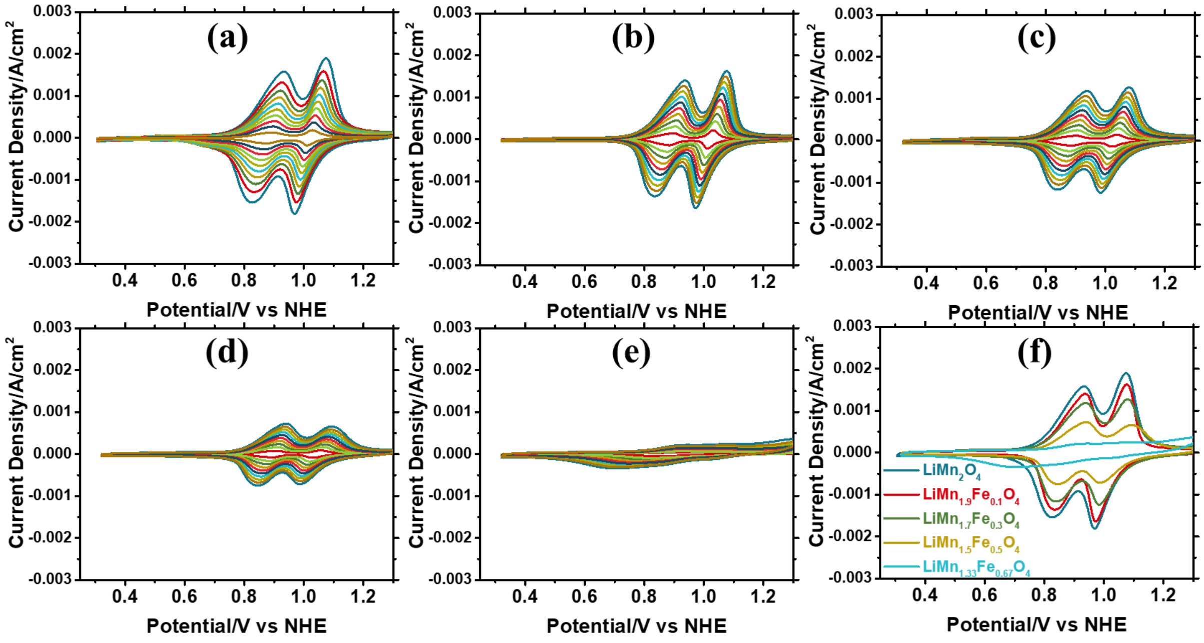


**Figure S17.** The scan rate dependent (2 to 20 mV/s with a 2 mV/s increments) CV curves of the F-$LiMn_{2-x}Fe_xO_4$ electrodes in 1 M $LiNO_3$ electrolyte solutions, where x is (a) 0, (b) 0.1, (c) 0.3, (d) 0.5, and (e) 0.67 and (f) CV curves of all compositions at 20 mV/s scan rate.

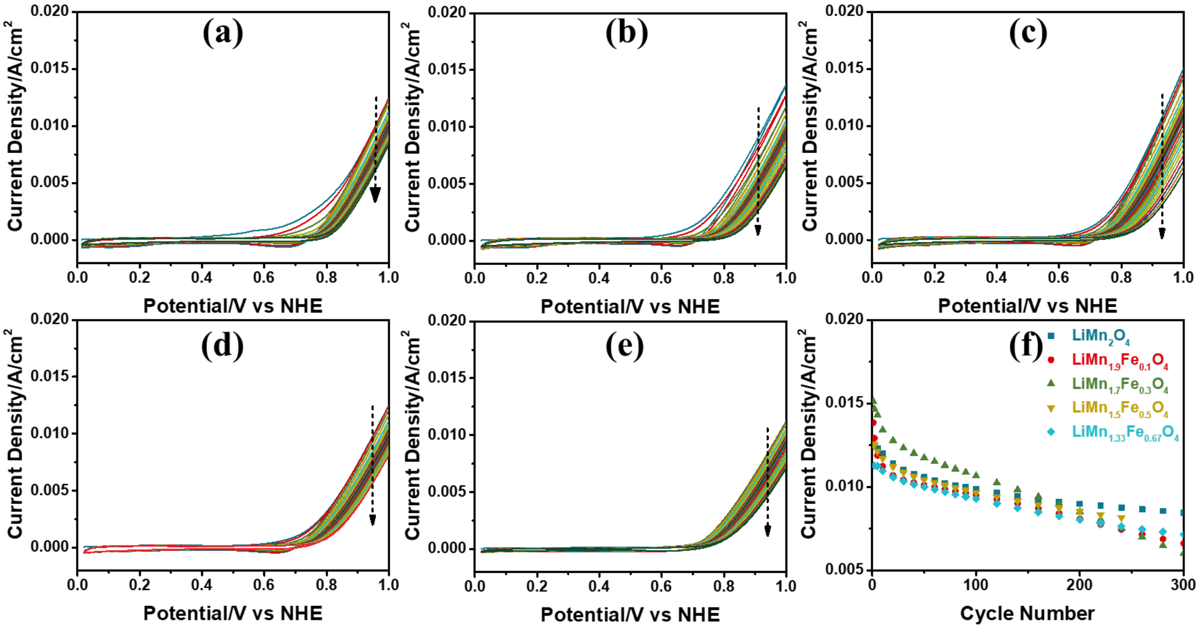


**Figure S18.** 300 CV curves of the F-$LiMn_{2-x}Fe_xO_4$ electrodes in 1 M KOH solution with sweep rate of 50 mV/s, where x is (a) 0, (b) 0.1, (c) 0.3, (d) 0.5, (e) 0.67, and (f) CV cycle number vs current density (at 1 V) plot.

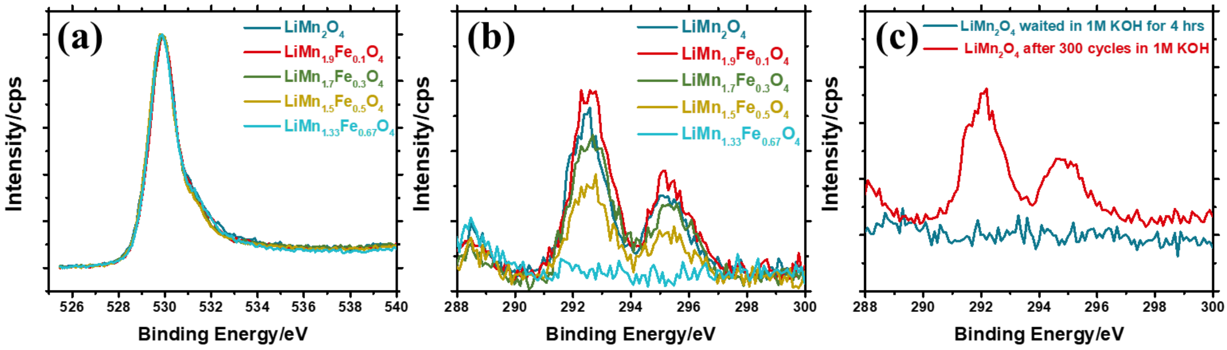


**Figure S19.** XPS spectra of the used in 300 CVs F-$LiMn_{2-x}Fe_xO_4$ electrodes in the (a) O1s, (b) K 2p regions, and, (c) XPS K 2p spectra of the F-$LiMn_{2-x}Fe_xO_4$ electrodes used in 300 CVs and kept in 1 M KOH for 4 hours.

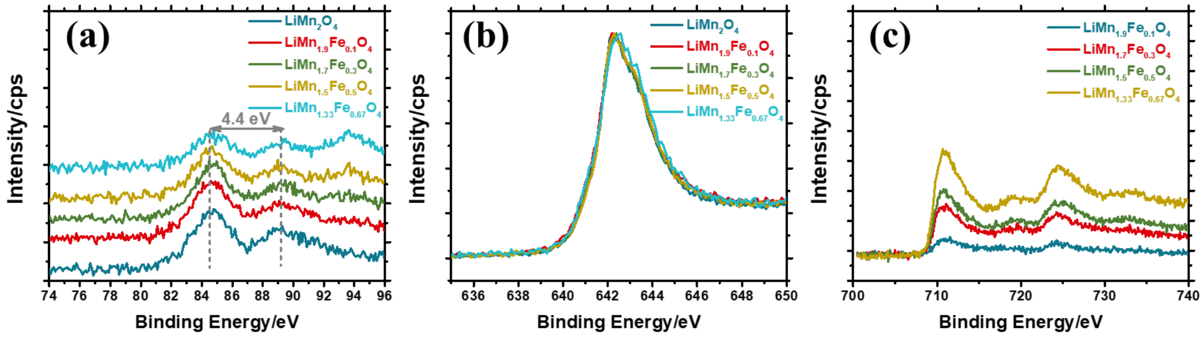


**Figure S20.** XPS spectra of the used in 300 CVs F-$LiMn_{2-x}Fe_xO_4$ electrodes in the (a) Mn 3s, (b) Mn2p ($^2P_{3/2}$), and (c) Fe 2p regions.

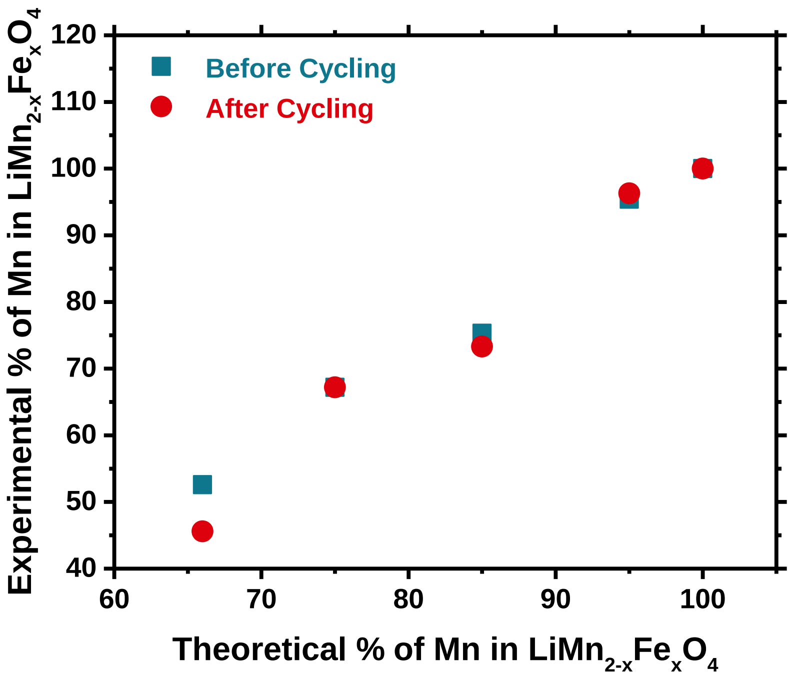


**Figure S21.** The XPS survey analysis results of the F-$LiMn_{2-x}Fe_xO_4$ electrodes, before (blue squares) and after 300 CVs (red dots) in 1 M KOH electrolyte solution.

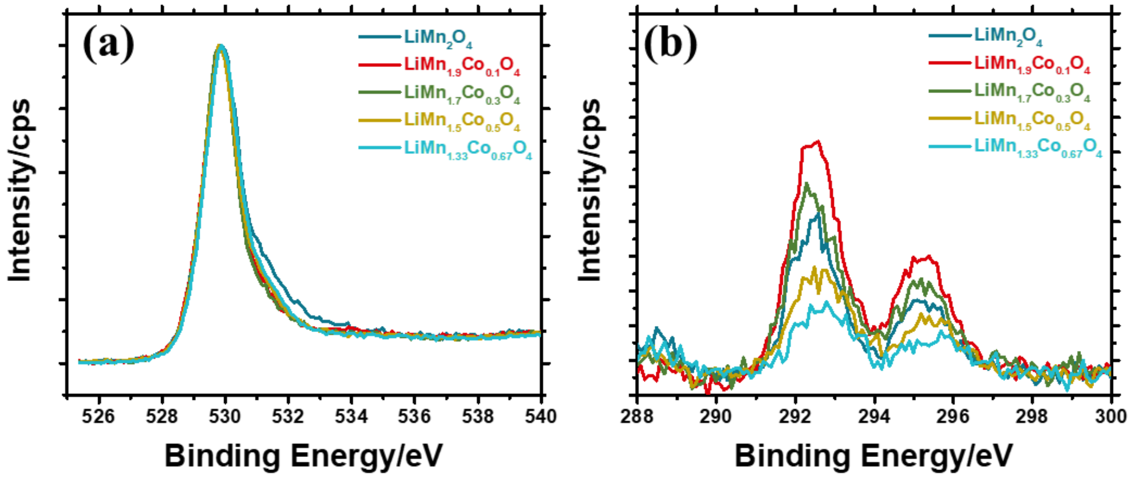


**Figure S22.** XPS spectra of the F-$LiMn_{2-x}Co_xO_4$ electrodes after 300 CVs in the (a) O 1s and (b) K 2p regions.

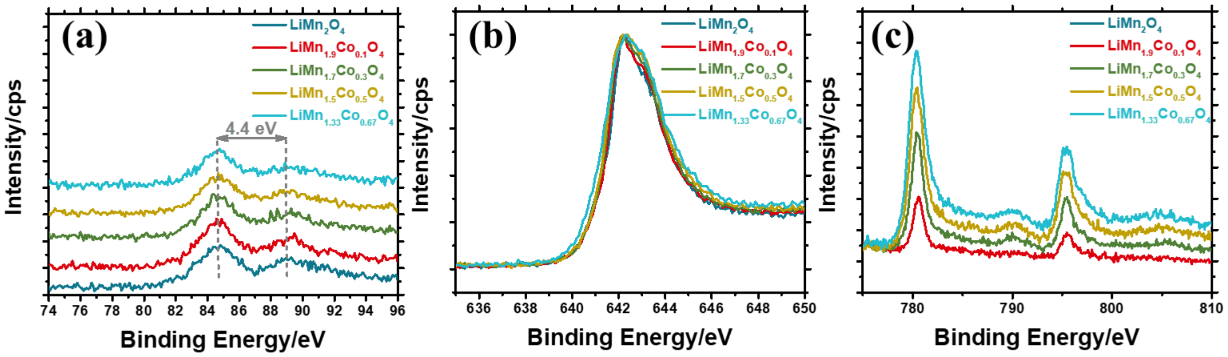


**Figure S23.1** XPS spectra of the $LiMn_{2-x}Co_xO_4$ electrodes after 300 cycles: (a) Mn 3s, (b)Mn 2p ($^2P_{3/2}$), and (c) Co 2p regions.

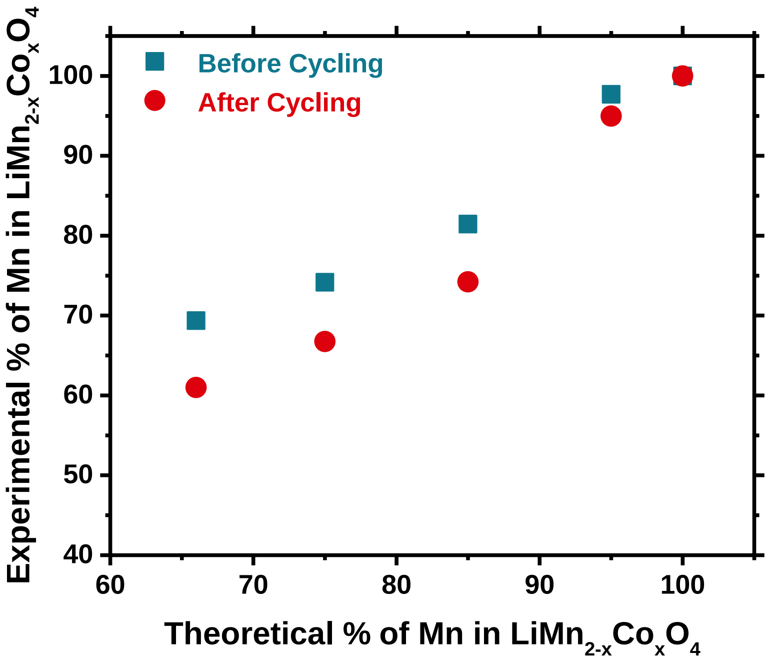


**Figure S24.** The XPS survey analysis results of the F-$LiMn_{2-x}Co_xO_4$ electrodes, before (blue squares) and after 300 CVs (red dots) in 1 M KOH electrolyte solution.

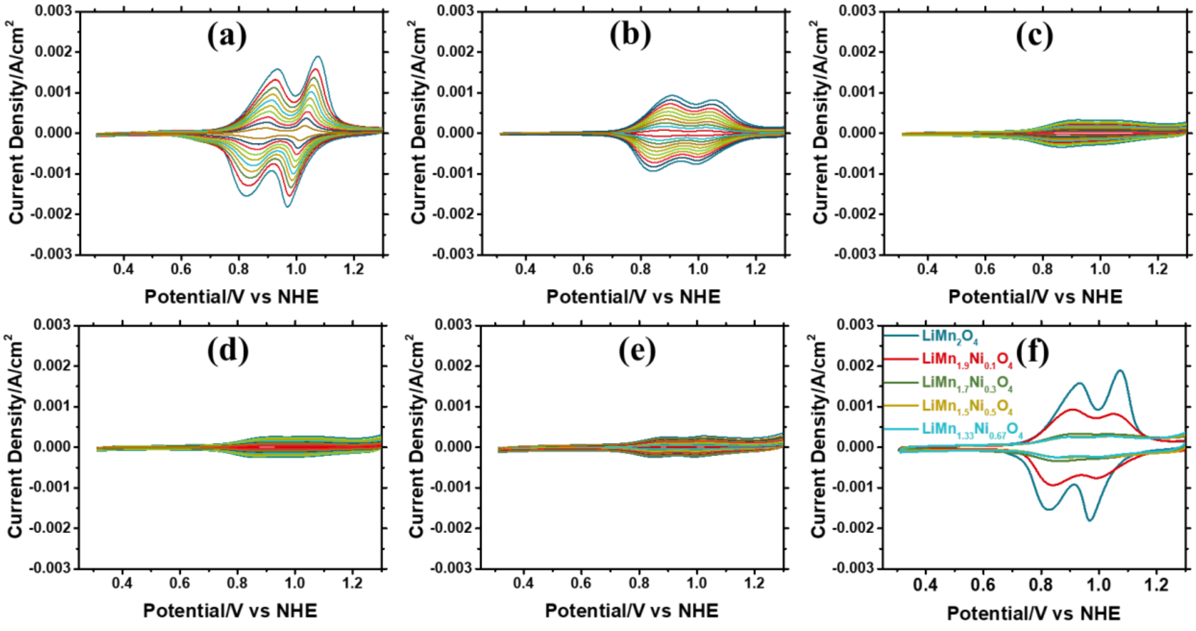


**Figure S25**. The scan rate dependent (2 to 20 mV/s with a 2 mV/s increments) CV curves of the F-$LiMn_{2-x}Ni_xO_4$ electrodes in 1 M $LiNO_3$ electrolyte solutions, where x is (a) 0, (b) 0.1, (c) 0.3, (d) 0.5, and (e) 0.67 and (f) CV curves of all compositions at 20 mV/s scan rate.

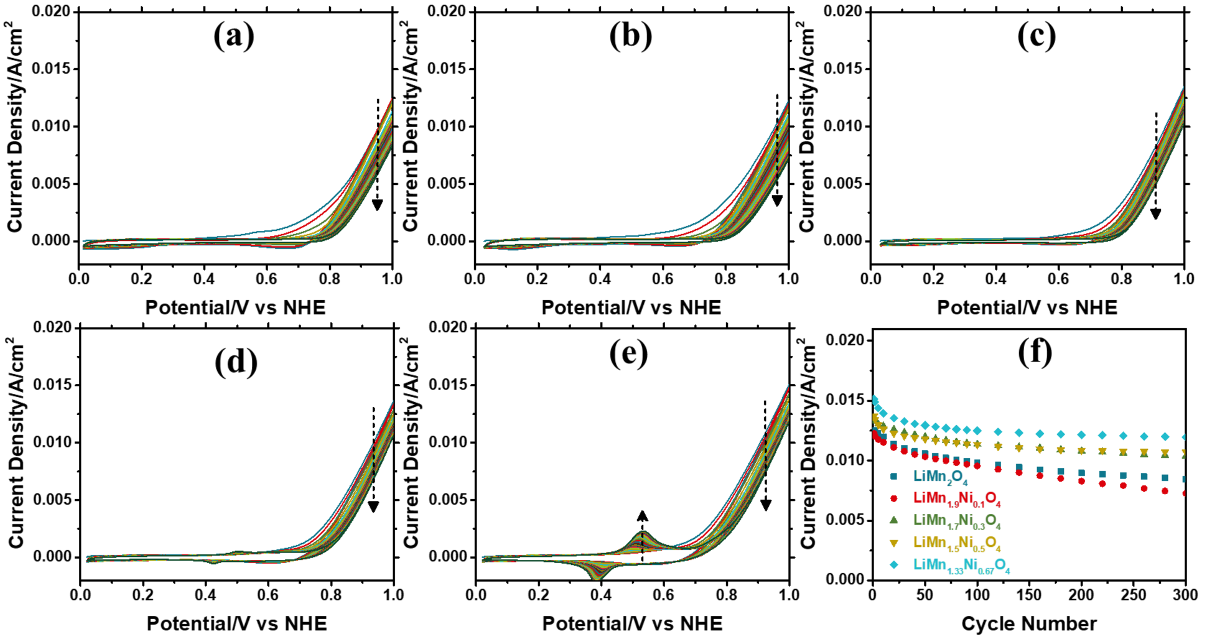


**Figure S26.** 300 CV curves of the F-$LiMn_{2-x}Ni_xO_4$ electrodes in 1 M KOH solution with sweep rate of 50 mV/s, where x is (a) 0, (b) 0.1, (c) 0.3, (d) 0.5, (e) 0.67, and (f) CV cycle number vs current density (at 1 V) plot.

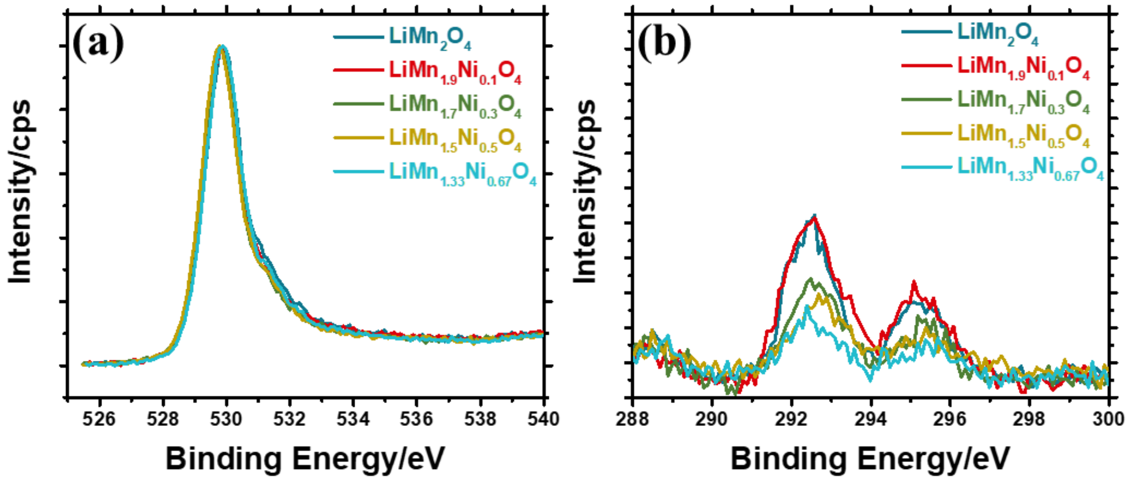


**Figure S27.** The XPS spectra of the F-$LiMn_{2-x}Ni_xO_4$ electrodes, after 300 CV cycles in the (a) O 1s and (b) K 2p regions.

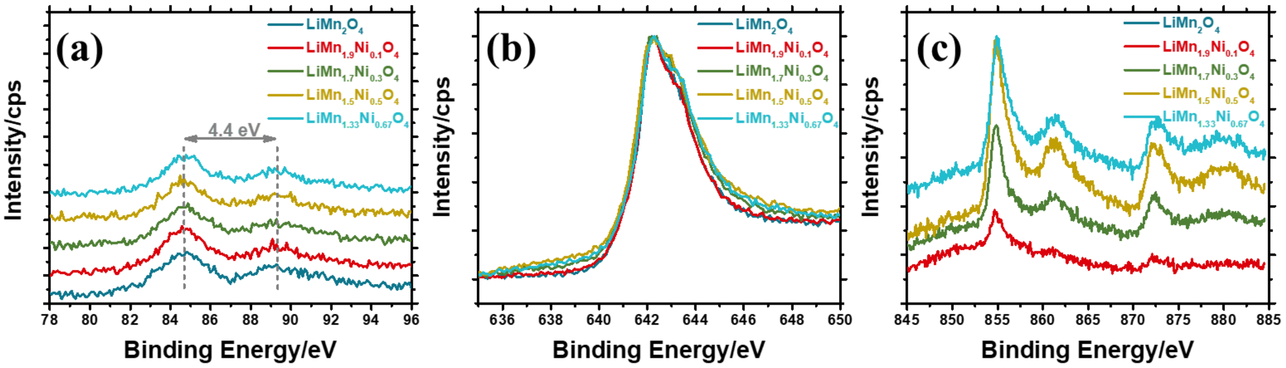


**Figure S28.** The XPS spectra of the F-$LiMn_{2-x}Ni_xO_4$ electrodes, after 300 CV cycles in the (a) Mn 3s, (b) Mn 2p ($^2P_{3/2}$), and (c) Ni 2p regions.

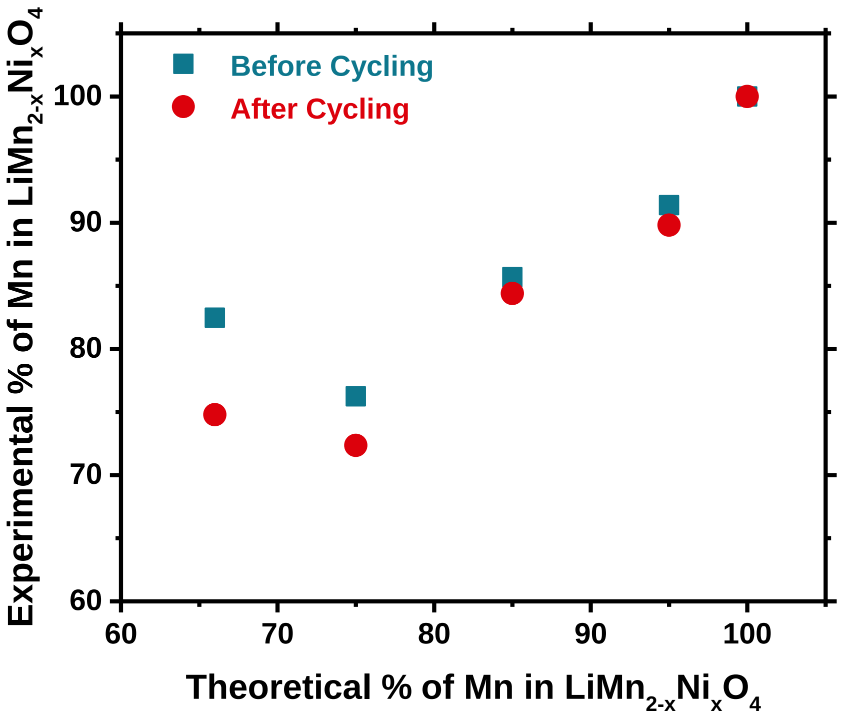


**Figure S29.** The XPS survey analysis results of the F-$LiMn_{2-x}Ni_xO_4$ electrodes, before (blue squares) and after 300 CVs (red dots) in 1 M KOH electrolyte solution.

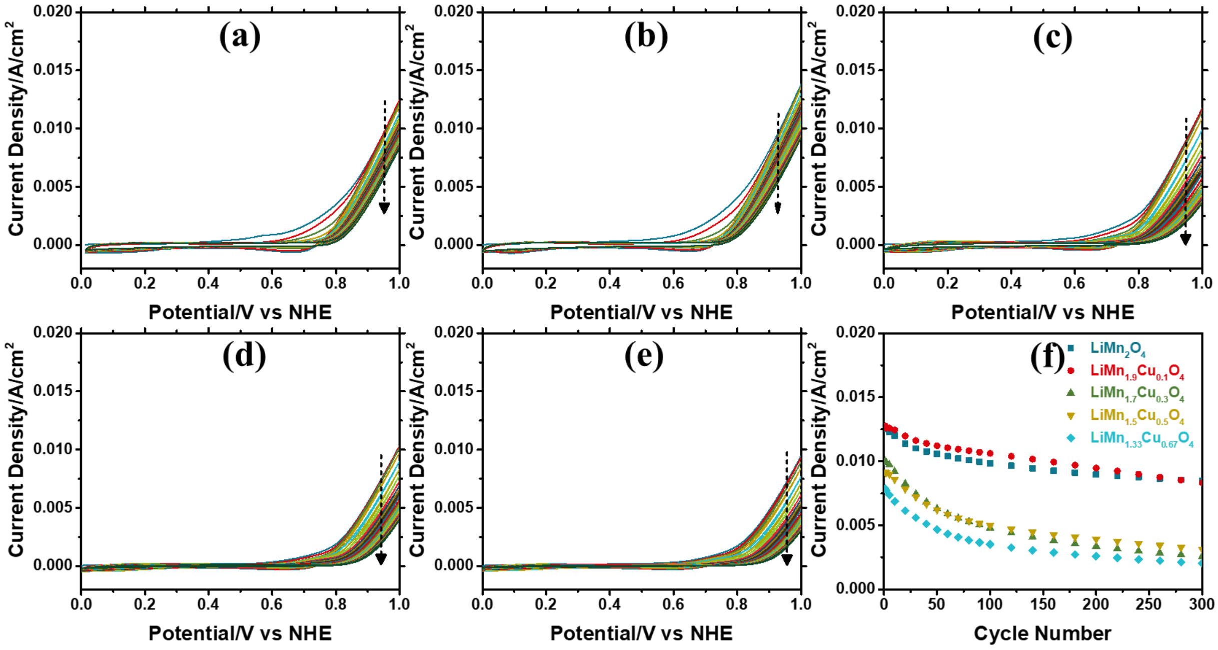


**Figure S30.** 300 CV curves of the F-$LiMn_{2-x}Cu_xO_4$ electrodes in 1 M KOH solution with sweep rate of 50 mV/s, where x is (a) 0, (b) 0.1, (c) 0.3, (d) 0.5, (e) 0.67, and (f) CV cycle number vs current density (at 1 V) plot.

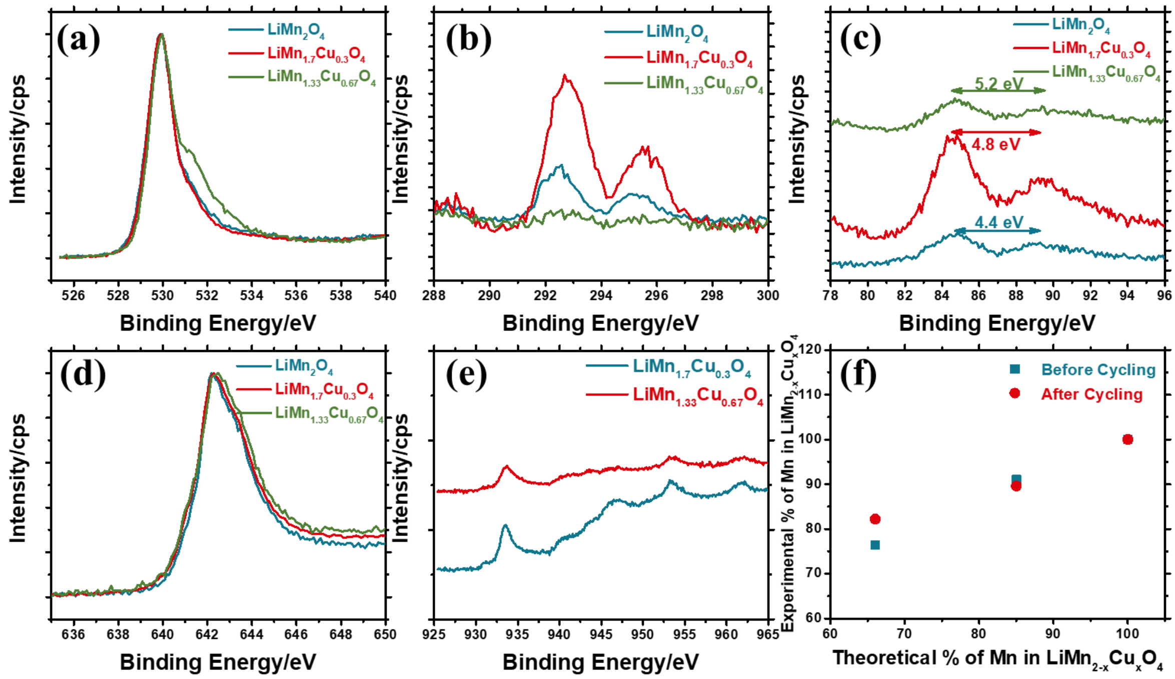


**Figure S31.** The XPS spectra of the F-$LiMn_{2-x}Cu_xO_4$ electrodes after 300 CVs in the (a) O 1s, (b) K 2p, (c) Mn 3s, (d) Mn 2p ($^2P_{3/2}$), and (e) Cu 2p regions. (f) the XPS survey analysis results of the $LiMn_{2-x}Cu_xO_4$ electrodes before and after CV cycling in 1 M KOH solution (blue squares: before, red dots: after cycling).

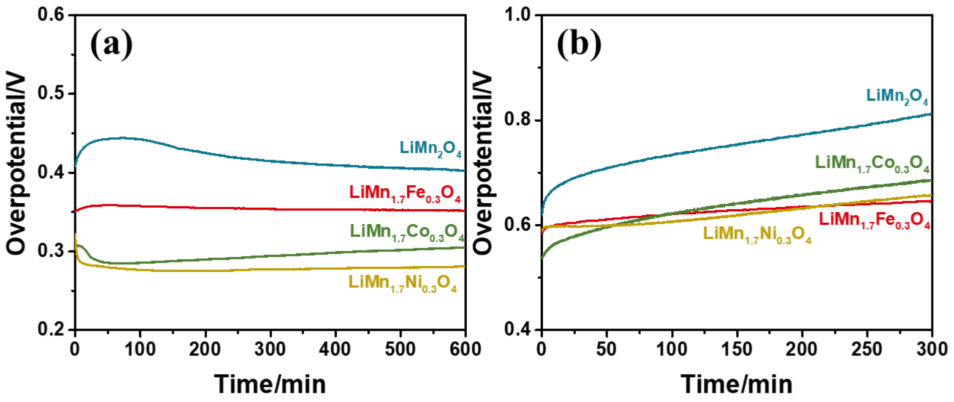


**Figure S32.** The CP curves of the F-$LiMn_{1.7}M_{0.3}O_4$ electrodes in 1M KOH at (c) 1 and (d) 10 $mA/cm^2$ current densities.

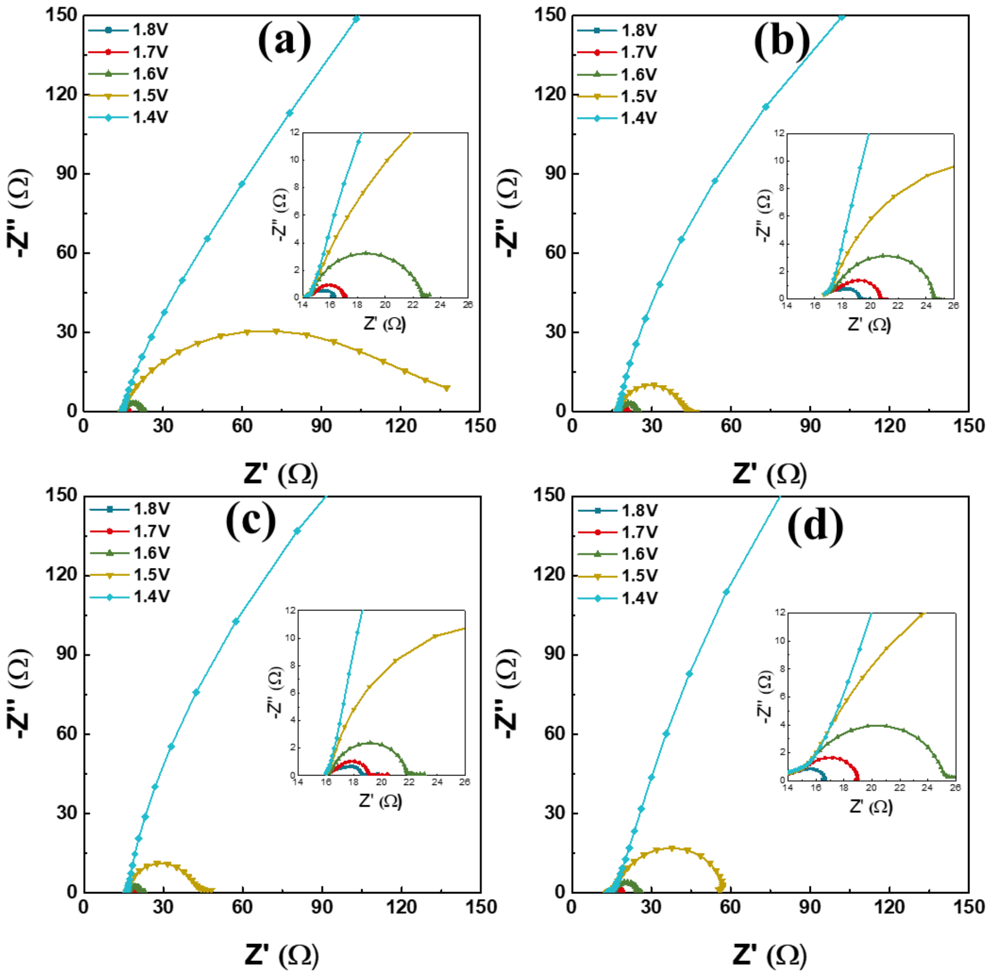


**Figure S33.** Nyquist plots of the F-$LiMn_{1.7}M_{0.3}O_4$ electrodes at several potentials (vs. RHE) in 1 M KOH electrolyte, where M is (a) Mn, (b) Fe, (c) Co, and (d) Ni.

**Table S1.** Compositions of the mother liquors for electrode preparations (all chemical quantities are in grams).

| Electrode | $LiNO_3$ | $[Mn(OH_2)_4](NO_3)_2$ | CTAB | P123 | $HNO_3$ | EtOH |
|---|---|---|---|---|---|---|
| $LiMn_2O_4$ | 0.173 | 1.255 | 0.045 | 0.725 | 0.5 | 5 |

| Electrodes | $LiNO_3$ | $[Mn(OH_2)_4](NO_3)_2$ | $[Fe(OH_2)]_9(NO_3)_3$ | CTAB | P123 | $HNO_3$ | EtOH |
|---|---|---|---|---|---|---|---|
| $LiMn_{1.9}Fe_{0.1}O_4$ | 0.173 | 1.192 | 0.101 | 0.045 | 0.725 | 0.5 | 5 |
| $LiMn_{1.7}Fe_{0.3}O_4$ | 0.173 | 1.066 | 0.303 | 0.045 | 0.725 | 0.5 | 5 |
| $LiMn_{1.5}Fe_{0.5}O_4$ | 0.173 | 0.941 | 0.505 | 0.045 | 0.725 | 0.5 | 5 |
| $LiMn_{1.33}Fe_{0.67}O_4$ | 0.173 | 0.836 | 0.671 | 0.045 | 0.725 | 0.5 | 5 |

| Electrodes | $LiNO_3$ | $[Mn(OH_2)_4](NO_3)_2$ | $[Co(OH_2)]_6(NO_3)_2$ | CTAB | P123 | $HNO_3$ | EtOH |
|---|---|---|---|---|---|---|---|
| $LiMn_{1.9}Co_{0.1}O_4$ | 0.173 | 1.192 | 0.073 | 0.045 | 0.725 | 0.5 | 5 |
| $LiMn_{1.7}Co_{0.3}O_4$ | 0.173 | 1.066 | 0.218 | 0.045 | 0.725 | 0.5 | 5 |
| $LiMn_{1.5}Co_{0.5}O_4$ | 0.173 | 0.941 | 0.364 | 0.045 | 0.725 | 0.5 | 5 |
| $LiMn_{1.33}Co_{0.67}O_4$ | 0.173 | 0.836 | 0.483 | 0.045 | 0.725 | 0.5 | 5 |

| Electrodes | $LiNO_3$ | $[Mn(OH_2)_4](NO_3)_2$ | $[Ni(OH_2)]_6(NO_3)_2$ | CTAB | P123 | $HNO_3$ | EtOH |
|---|---|---|---|---|---|---|---|
| $LiMn_{1.9}Ni_{0.1}O_4$ | 0.173 | 1.192 | 0.073 | 0.045 | 0.725 | 0.5 | 5 |
| $LiMn_{1.7}Ni_{0.3}O_4$ | 0.173 | 1.066 | 0.218 | 0.045 | 0.725 | 0.5 | 5 |
| $LiMn_{1.5}Ni_{0.5}O_4$ | 0.173 | 0.941 | 0.364 | 0.045 | 0.725 | 0.5 | 5 |
| $LiMn_{1.33}Ni_{0.67}O_4$ | 0.173 | 0.836 | 0.483 | 0.045 | 0.725 | 0.5 | 5 |

| Electrodes | $LiNO_3$ | $[Mn(OH_2)_4](NO_3)_2$ | $[Cu(OH_2)]_3(NO_3)_2$ | CTAB | P123 | $HNO_3$ | EtOH |
|---|---|---|---|---|---|---|---|
| $LiMn_{1.9}Cu_{0.1}O_4$ | 0.173 | 1.192 | 0.604 | 0.045 | 0.725 | 0.5 | 5 |
| $LiMn_{1.7}Cu_{0.3}O_4$ | 0.173 | 1.066 | 0.181 | 0.045 | 0.725 | 0.5 | 5 |
| $LiMn_{1.5}Cu_{0.5}O_4$ | 0.173 | 0.941 | 0.302 | 0.045 | 0.725 | 0.5 | 5 |
| $LiMn_{1.33}Cu_{0.67}O_4$ | 0.173 | 0.836 | 0.401 | 0.045 | 0.725 | 0.5 | 5 |

**Table S2.** Mol ratios of chemicals in the solutions and compositions in the calcined products.

| Mol ratios of the chemicals in the solutions Li-Mn-M-CTAB-P123 | Compounds |
|---|---|
| 20Li-40Mn-1CTAB-1P123 | $LiMn_2O_4$ |
| 20Li-38Mn-2M-1CTAB-1P123 | $LiMn_{1.9}M_{0.1}O_4$ |
| 20Li-34Mn-6M-1CTAB-1P123 | $LiMn_{1.7}M_{0.3}O_4$ |
| 20Li-30Mn-10M-1CTAB-1P123 | $LiMn_{1.5}M_{0.5}O_4$ |
| 20Li-26.6Mn-13.3M-1CTAB-1P123 | $LiMn_{1.33}M_{0.67}O_4$ |

**Table S3.** Our Tafel slopes and overpotentials at various current densities, compared to similar compounds in recent literatures.

| Materials | Tafel Slope (mV/dec) | $\eta_{10\ mA/cm2}$ (mV) | $\eta_{20\ mA/cm2}$ (mV) | $\eta_{50\ mA/cm2}$ (mV) | References |
|---|---|---|---|---|---|
| $FeMn_2O_4$/GC | 105 | 360 | - | - | 52 |
| $FeMn_2O_4$/NF | 28 | - | 252 | - | 53 |
| $FeMn_2O_4$ | 100 | 430 | - | - | 54 |
| $CoMn_2O_4$/NF | 105 | 367 | - | - | 55 |
| $CoMn_2O_4$/FTO | 26 | 300 | - | - | 56 |
| $CoMn_2O^4$/CNF | 82 | 337 | - | - | 57 |
| $NiMn_2O_4$/NF | 218 | 250 | - | - | 58 |
| $NiMn_2O_4$/CNS | 89 | 199 | - | - | 59 |
| F-$LiMn_{1.7}Fe_{0.7}O_4$ | 44 | 645 | 901 | - | This work |
| G-$LiMn_{1.7}Fe_{0.7}O_4$ | 66 | 459 | 555 | - | This work |
| F-$LiMn_{1.7}Co_{0.7}O_4$ | 48 | 686 | 1027 | - | This work |
| G-$LiMn_{1.7}Co_{0.7}O_4$ | 57 | 440 | 506 | 692 | This work |
| F-$LiMn_{1.7}Ni_{0.7}O_4$ | 33 | 656 | 899 | 1596 | This work |
| G-$LiMn_{1.7}Ni_{0.7}O_4$ | 60 | 535 | 604 | 866 | This work |
